# Subcycle videography of Landau–Zener–Majorana transitions in graphene

**DOI:** 10.1038/s41586-026-11214-y

**Published:** 2026-10-28

**Authors:** V. Eggers, G. Inzani, M. Meierhofer, L. Münster, J. Helml, R. Wallauer, S. Zajusch, S. Ito, L. Machtl, H. Yin, C. Kumpf, F. C. Bocquet, C. Bao, J. Güdde, F. S. Tautz, R. Huber, U. Höfer

**Affiliations:** 1 Department of Physics, Regensburg Center for Ultrafast Nanoscopy (RUN) and Halle-Berlin-Regensburg Cluster of Excellence CCE, University of Regensburg, Regensburg, Germany; 2 Department of Physics, Philipps-Universität Marburg, Marburg, Germany; 3 Peter Grünberg Institut (PGI-3), Forschungszentrum Jülich, Jülich, Germany; 4 Jülich Aachen Research Alliance (JARA), Fundamentals of Future Information Technology, Jülich, Germany; 5 Experimentalphysik IV A, RWTH Aachen University, Aachen, Germany

**Keywords:** Quantum mechanics, Electronic properties and materials, Two-dimensional materials, Ultrafast photonics

## Abstract

Strong light fields have unlocked entirely new possibilities to tailor coherent electron trajectories, engineer band structures and shape emergent phases of matter all-optically^1–9^. Unravelling the underlying quantum mechanisms requires a visualization of the lightwave-driven electron motion^10,11^ directly in the band structure. Although photoelectron momentum microscopy has imaged photoexcited electrons averaged over many cycles of light^12–19^, subcycle band-structure videography has been limited to small electron momenta^8,20^. Yet, lightwave-driven elementary processes in quantum materials often occur throughout momentum space. Here we introduce attosecond-precision, subcycle band-structure videography covering the entire first Brillouin zone and visualize one of the most fundamental but elusive strong-field processes: non-adiabatic Landau–Zener–Majorana tunnelling. The interplay of strong-field-driven acceleration within the Dirac-like band structure of graphene and periodic Landau–Zener–Majorana interband tunnelling manifests in a coherent subcycle displacement and distortion of the carrier distribution at the Brillouin zone edge. The extremely non-thermal electron distributions also allow us to disentangle competing scattering processes and assess their impact on coherent electronic control. Our panoramic view of field-driven electron motion enables a new quality of microscopic understanding of strong-field control of quantum materials.

## Abstract (web summary)

An experimental concept enabling attosecond-precision, subcycle band-structure videography covering the entire first Brillouin zone is demonstrated and used to visualize the lightwave-driven electron motion of Landau–Zener–Majorana transitions in graphene.

## Main

According to the adiabatic theorem, a time-dependent, two-level quantum system remains in its ground state during a slow evolution^21^. By contrast, driving the system faster than its intrinsic timescale through an avoided energy crossing can create a finite occupation of the excited state by means of non-adiabatic transitions called Landau–Zener–Majorana (LZM) tunnelling^22–24^. This mechanism is central for strong-field light–matter interaction^7,10,25,26^. Yet, a direct observation of this foundational quantum effect in momentum space is still missing. The unique properties of graphene^27^ form the ideal test bed for observing LZM transitions. Its quasirelativistic electron dispersion and the robustness under irradiation with intense light have made this material a prime test ground for lightwave electronics^26,28^, including the demonstration of the first lightwave electronic logic gates^7^ and Floquet–Bloch states^18,19^. This has opened revolutionary prospects for quantum control, quantum sensing and quantum computation applications^10,11,29^.

Understanding and shaping the opportunities offered by lightwave control of electrons requires a direct observation of how LZM transitions dictate strong-field light–matter interaction. Attosecond spectroscopy has reached sufficient temporal resolution to access these dynamics^30,31^, and time- and angle-resolved photoemission spectroscopy (trARPES) in combination with momentum microscopy^32^ has visualized electron dynamics directly in the band structure of quantum materials or in the orbitals of molecular layers^12–19,33^. trARPES with subcycle temporal resolution^8,20^ has combined these approaches but offered only limited 1D momentum space access. To track coherent strong-field phenomena and scattering-induced momentum redistribution, observing carrier dynamics across the entire momentum space is imperative.

Here we expand subcycle band-structure videography to the entire first Brillouin zone (BZ). The new platform overcomes previous restrictions of momentum microscopy and subcycle ARPES, increasing the momentum area accessible with subcycle resolution by more than two orders of magnitude and the available field strengths by one order of magnitude. Exploiting few-cycle mid-infrared (MIR) field transients with large peak amplitudes, we drive coherent electron dynamics at the BZ edge of graphene. This approach reveals the simultaneous displacement and distortion of the light-driven electron distribution, uncovering the interplay between coherent intraband and interband dynamics underlying LZM transitions in graphene. Moreover, tracking the non-thermal subcycle distributions in 2D momentum space reveals how different scattering processes affect lightwave-driven dynamics.

## Subcycle lightwave currents

**Fig. 1:**
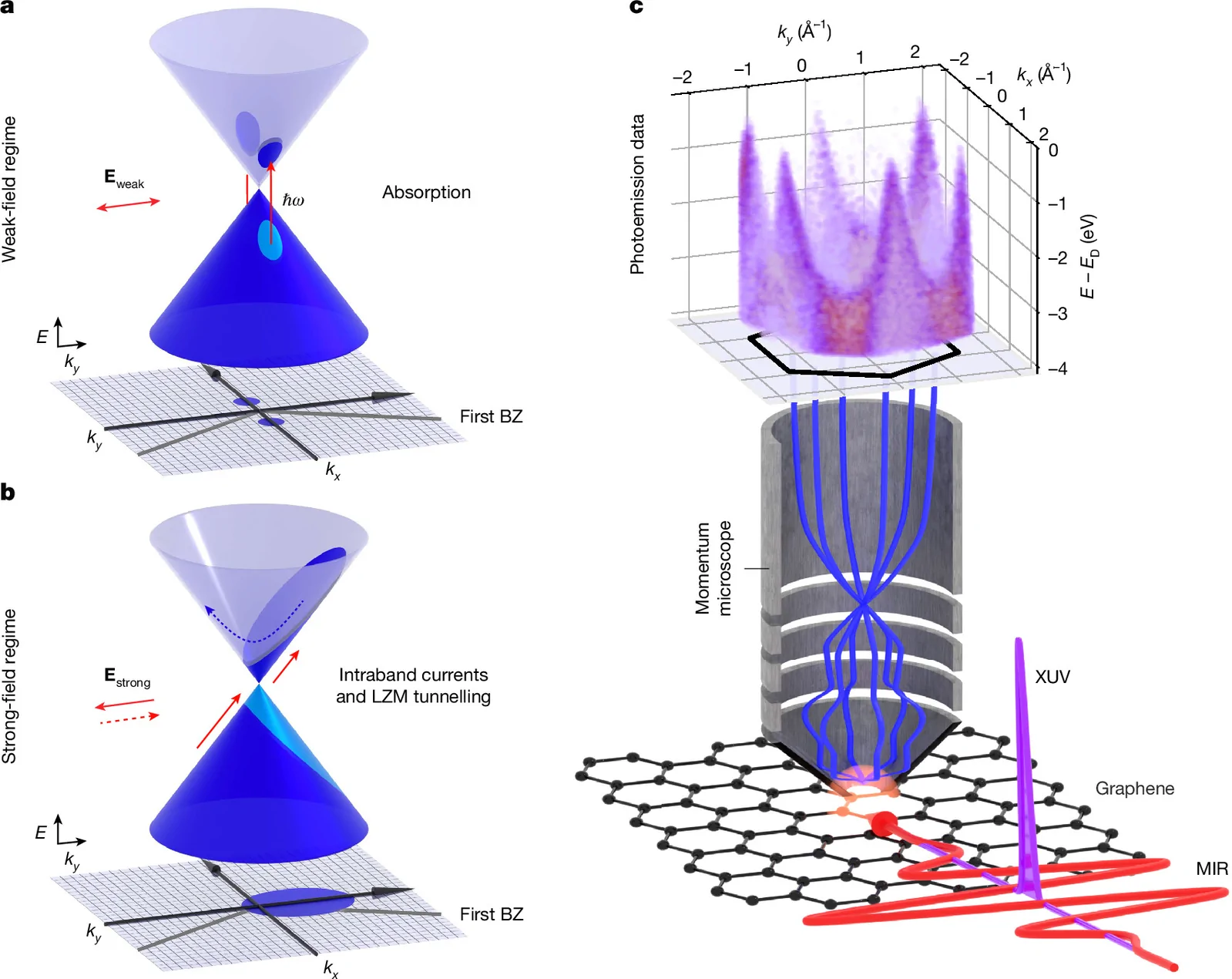
LZM transitions in 2D momentum space. **a**, Illustration of the fundamental electronic processes governing light–matter interaction in undoped graphene at the K point in the weak-field regime. Here electrons can be promoted from the valence band to the conduction band by absorption. In graphene, the optical selection rules favour transitions in the momentum direction perpendicular to the polarization of the electric field. **b**, In the strong-field regime, electrons can undergo ballistic acceleration by an electric field (dashed blue arrow). Simultaneously, the field can drive interband LZM transitions on subcycle timescales, allowing electrons to tunnel from the valence band to the conduction band (solid red arrows). This results in an elongated electron distribution in the previously unoccupied conduction band, with the weight of the electron population in the direction parallel to the field. **c**, In the experimental scheme shown in the bottom half, an *s*-polarized, phase-stable MIR pulse (red) drives a monolayer of graphene, and a *p*-polarized XUV probe pulse (purple) at variable time delay, *t*, provides snapshots of the induced dynamics with subcycle temporal resolution. The photoemitted electrons (blue) enter a photoemission momentum microscope. This enables the simultaneous detection of both parallel momenta, *k*_*x*_ and *k*_*y*_, and kinetic energy for every individual electron. The upper half shows a femtosecond snapshot of the 2D band structure of a monolayer of graphene, recorded with the momentum microscope. The field of view extends over the entire first BZ, allowing tracking of the electronic occupation to slightly beyond the six K points.

When low-intensity, short-wavelength light interacts with electrons in a solid, it can induce perturbative interband quantum jumps in momentum regions in which the occupied and unoccupied bands are separated by an integer multiple of the photon energy (Fig. 1a). In graphene, the non-trivial pseudospin texture influences the dipole moments between the lower and upper halves of the Dirac cone, leading to a distribution of excited carriers only along the direction orthogonal to the light field^34,35^. Conversely, a strong low-frequency field transient can adiabatically accelerate carriers within their bands on subcycle timescales, deflecting the Fermi surface in momentum space according to Bloch’s acceleration theorem (Fig. 1b). During their excursion, electrons experience an energy gap, which, in graphene, varies linearly near the K point to form an avoided crossing. Thus, field-driven acceleration may drive prototypical LZM transitions, which leave a markedly different occupation in momentum space compared with Fig. 1a. If the driving field is periodic, the phase accumulated between consecutive non-adiabatic events—the Stückelberg phase—results in so-called Landau–Zener–Stückelberg–Majorana (LZSM) interference^36,37^.

To access this regime, we drive electrons in epitaxially grown monolayer graphene on SiC (ref. ^38^) with intense phase-stable MIR pulses (central frequency, 30 THz). After a delay time, *t*, extreme ultraviolet (XUV) pulses (central photon energy, 21.7 eV) photoemit the electrons with subcycle resolution. Their energies and momenta are detected with a time-of-flight photoemission momentum microscope (Fig. 1c and Methods). In equilibrium, the photoemission signal maps the occupied band structure. The high XUV photon energy and the large acceptance angle of the momentum microscope capture the electronic structural dynamics throughout the entire first BZ, including its six corners, in a single measurement with sub-10-fs resolution.

**Fig. 2:**
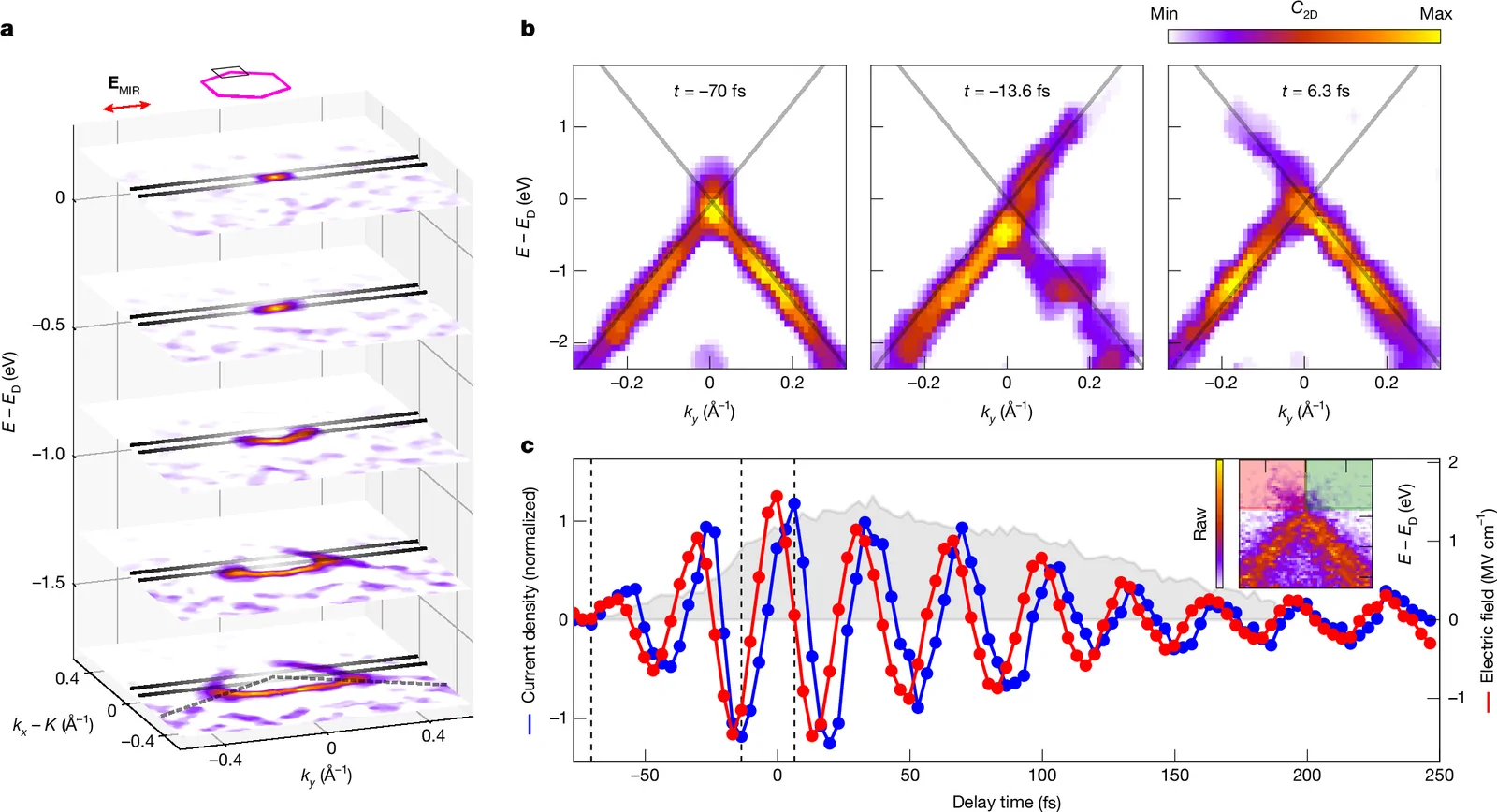
Lightwave-driven currents in graphene. **a**, Zoomed-in curvature-filtered (Methods) cuts through the band structure at a selected K point. The red arrow (top) indicates the MIR polarization. The dashed grey line (bottom) marks the BZ border. The solid black lines represent the integration range in momentum space used in the three panels in **b**. **b**, Streaking-compensated and curvature-filtered view into the band structure of graphene at the Dirac point before the arrival of the MIR pulse (*t* = −70 fs) and for selected delay times during the interaction with the MIR pulse, *t* = −13.6 fs and *t* = 6.3 fs (raw data, Extended Data Fig. 2b). The last two snapshots show how the electron distribution is accelerated along the Dirac cone away from the equilibrium Fermi level. **c**, Current extracted from the occupation imbalance in the photoemission maps (blue points) and MIR waveform reconstructed from the momentum streaking (red points). Dashed black lines mark the temporal positions shown in **b**. The lightwave-driven current is extracted by tracking the imbalance in the occupation for positive and negative momenta (red and green shaded areas) in the raw photoemission spectra (inset) and normalized on the total amount of carriers in both regions before the pulse arrives. The grey shaded area indicates the normalized population increase in the conduction band extracted from a similar measurement.

Figure 2a shows several momentum distribution maps in the vicinity of a K point at selected energies. The in-plane component of the *p*-polarized XUV radiation driving the photoemission process is set parallel to the *k*_*x*_-axis. Owing to the matrix elements in the photoemission process, the intensity exhibits the characteristic horseshoe-like distribution for energies far below the Dirac point^39^. The influence of the dark corridor at *k*_*y*_ = 0 and positive *k*_*x*_ − *K* values is suppressed for energies close to the Dirac point, *E*_D_, and the photoemission intensity forms a disc. To ensure that the relevant dynamics occur along a direction in which the two branches of the Dirac cone have equal photoemission intensities, we apply an *s*-polarized MIR field along the *k*_*y*_-axis. For large negative delays (*t* = −70 fs), a slice of the photoelectron intensity for *k*_*x*_ − *K* = 0 (Fig. 2b) shows two linear bands with a slope of *ħv*_F_ (with the Fermi velocity *v*_F_ = 10.7 Å fs^−1^) and a Fermi level located 200 meV above the Dirac point. With increasing *t*, we observe an apparent shift of the overall band structure owing to momentum streaking^20^ of photoemitted electrons in vacuo by the MIR field. This effect can be used to retrieve the MIR waveform at the sample surface (Extended Data Fig. 2a and Methods). Compensating for this shift isolates the subcycle electron dynamics within the band structure.

A corresponding femtosecond snapshot of the band structure for *t* = −13.6 fs shows a highly non-thermal lightwave-driven electron distribution (Fig. 2b). In the upper half of the Dirac cone (*E* − *E*_D_ ≥ 0), the *k*_*y*_ > 0 branch is occupied up to about 1 eV, whereas the *k*_*y*_ < 0 branch is completely unoccupied. Half a cycle of the MIR carrier wave later (*t* = 6.3 fs), the field direction is inverted and the electron distribution is mirrored. The extreme carrier redistribution on subcycle timescales clearly proves the non-perturbative nature of the light–matter interaction. This scenario is expected for the small Keldysh parameter near the Dirac point (*γ* = 0.075; Methods). Still, it is notable that the few-fs timescales accessible with our experiment allow us to see lightwave-driven intraband acceleration despite the extremely fast scattering processes in graphene^33,35,40–45^. Asymmetric electron distributions in momentum space correspond to the excitation of an intraband current. By tracking the imbalance in the band occupation for positive and negative *k*_*y*_ momenta (Fig. 2c, inset and Methods), we extract the lightwave-driven intraband current density and directly compare it with the MIR driving waveform (Fig. 2c). Although the current density shows a similar envelope and oscillation frequency as the MIR transient, there is a non-trivial phase shift between the two waveforms.

From Bloch’s acceleration theorem^20,46^, we would expect that, in the absence of decoherence, the momentum accumulated by electrons follows the integral over the MIR field, resulting in a phase shift of π/2 between the current and the field. A Fourier analysis (Methods) allows us to retrieve this phase delay with attosecond precision. Notably, the average delay of 4.9 ± 0.2 fs is much smaller than the value associated with a fully coherent evolution (8.3 fs). Simulations based on the semiconductor Bloch equations (SBEs)^6^ in the relaxation time approximation (Extended Data Fig. 3 and Methods) reproduce this phase shift for a mean scattering time of 9.0 ± 0.7 fs. As non-adiabatic LZM transitions rely on the coherent and unperturbed field-driven evolution of charge carriers, this sets the time and length scales relevant for lightwave electronics. Despite the short scattering time, electrons in graphene can travel almost 100 Å owing to their large velocity of *v*_F_ = 10.7 Å fs^−1^.

## Interband coupling in electron dynamics

Most notably, the current is accompanied by an ultrafast increase of the total conduction-band population within the strongest MIR half-cycles (Fig. 2c, grey shaded area). Perturbative interband transitions are too weak to become effective on subcycle timescales and, in equilibrium, straightforward vertical interband transitions are prohibited in the doped sample by Pauli blocking. Hence, the observed electronic structural dynamics attest to an interplay between strong-field-driven intraband and interband currents. Next, we unveil the microscopic mechanisms by imaging the full 2D momentum space. With this capability, unavailable in previous subcycle photoemission experiments^8,20^, we can directly target the coherent light–matter interaction regime and the subsequent relaxation dynamics.

**Fig. 3:**
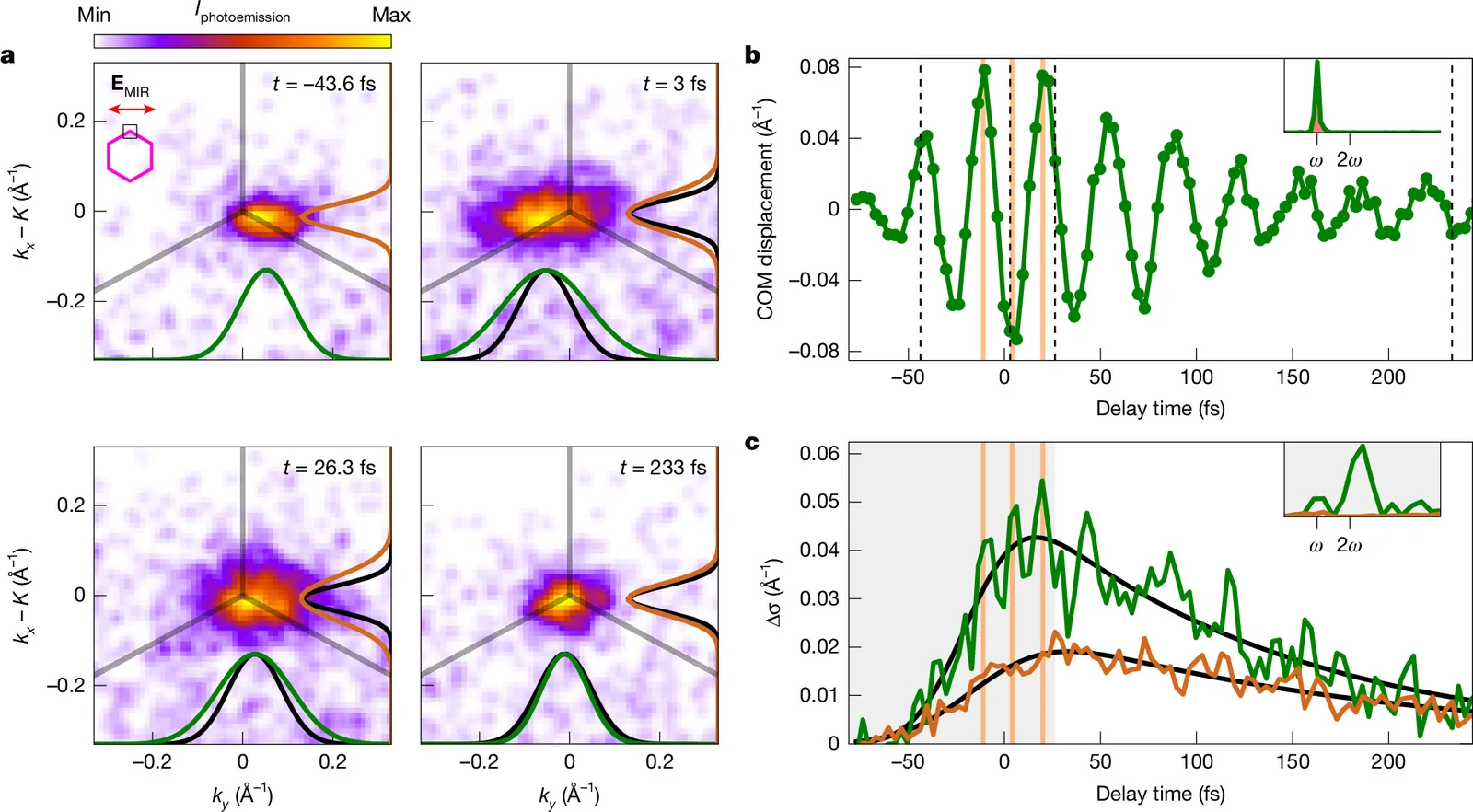
Subcycle electron dynamics in 2D momentum space. **a**, Streaking-compensated 2D carrier distribution in the conduction band for selected delay times. The inset indicates the position of the evaluated window in momentum space and the arrow shows the MIR polarization. The grey lines mark the BZ borders. The Gaussian fits illustrate the width of the projection of the carrier distribution on the *k*_*x*_-axis (brown) and the *k*_*y*_-axis (green). The fits of the first panel are shown in black in the later snapshots as a reference. Under the influence of the field, the carrier distribution is displaced along *k*_*y*_ (*t* = −43.6 fs). Owing to LZM transitions, the carrier distribution is elongated along *k*_*y*_, forming an ellipse (*t* = 3 fs). For later delays (*t* = 26.3 fs), scattering leads to a broadening also along *k*_*x*_. For vanishing fields, the electron distribution relaxes back to its original shape (*t* = 233 fs). **b**, Displacement of the COM of the electron distribution. The inset shows the Fourier transform of the oscillating electron distribution (green line) and the driving field (red shaded area). Dashed black lines mark the temporal positions of the momentum maps in **a**. **c**, Evolution of the change in the size of the electron distribution, Δσ, characterized by the width of the Gaussians used to fit the carrier distribution along the *k*_*x*_-axis (brown) and the *k*_*y*_-axis (green). The Fourier transform in the inset reveals oscillations in the width of the distribution along the *k*_*y*_-axis, with pronounced components at the second harmonic of the driving frequency during the initial half-cycles of the driving field (grey shaded area). The vertical orange lines in **b** and **c** are guides to the eye to relate the oscillation in the width of the electron distribution to the motion of its COM.

Before the interaction with the MIR transient, the equilibrium carrier distribution in the conduction band (*E* − *E*_D_ ≥ 0) of n-doped graphene can be approximated by a disc centred about each K point (Fig. 2a). If the electrons were solely subject to an accelerating electric field, they would, in accordance with Bloch’s acceleration theorem^46^, collectively move along the *k*_*y*_ direction and we would merely expect a time-dependent centre-of-mass (COM) displacement of this disc. Although the streaking-compensated 2D momentum maps (Fig. 3a) indeed show pronounced oscillations of the COM displacement with the frequency of the strong driving field and a maximum amplitude of 0.078 Å^−1^ (Fig. 3b), they also reveal a further notable change of their shape (Fig. 3a,c). The observed deviations from the initial shape of the distribution are on the same order as the COM displacement.

Already after the first MIR field cycles (Fig. 3a, *t* = 3 fs), the Fermi surface is strongly elongated from 0.054 Å^−1^ to 0.096 Å^−1^ in the direction parallel to the field, whereas the change in the orthogonal direction is only 0.014 Å^−1^ (Fig. 3a, Gaussian fits). The Fermi surface is no longer circular but takes on an elliptical shape. The time-dependent change in the *k*_*y*_ width (Fig. 3c, green line) directly correlates with the field strength. Furthermore, this width oscillates at twice the MIR carrier frequency while the COM displacement tracks the electric field similarly to the current plotted in Fig. 2c. Because the maximum broadening coincides with the peak COM displacement, we can rule out motion blur as the origin of the elongation in the *k*_*y*_ direction. Notably, the electron distribution encompasses the Dirac point at all delay times, even for the largest displacements.

All of these intriguing dynamics can be microscopically explained by a full quantum-mechanical solution of the SBEs^6^ (Methods), which account for intraband and interband transitions as well as LZM-type interband coupling, on equal footing. A systematic switch-off analysis pinpoints how specific aspects of coherent strong-field light–matter interaction manifest in the observed dynamics (Extended Data Fig. 4a). The periodic COM shift of the electron distribution is clearly associated with field-driven intraband motion of charge carriers. Yet, the distribution retains its circular shape and separates from the K point for large displacement if interband dynamics are switched off. Once interband coupling is included, the theory shows the same elongation of the carrier distribution as the experiment (Extended Data Fig. 4b). The theory also qualitatively reproduces the increase in carrier density in the conduction band (Extended Data Fig. 5), confirming that interband transitions at the K point act like a well that feeds electrons into the upper part of the Dirac cone on subcycle timescales.

These transitions originate from strong-field interband coupling, whereas conventional perturbative interband photoexcitation is suppressed by orders of magnitude under our experimental conditions (Extended Data Fig. 6). Owing to pseudospin selection rules^34,35^, photoexcitation would occur in momentum regions with |*k*_*x*_ − *K*| > 0, located perpendicular to the polarization of the incident light (Extended Data Fig. 6a). By contrast, the coupled intraband and interband dynamics of the LZM scenario leads to the observed unidirectional elongation of the electron distribution parallel to the electric field (Extended Data Fig. 6b). Simulations with varying field strengths confirm that this feature is characteristic of the strong-field regime (Extended Data Fig. 7).

The region in which LZM-type tunnelling can occur is determined by the field strength, *E*_0_, and the energy gap, which increases linearly with |*k*_*x*_ − *K*|. For *E*_0_ = 1.57 MV cm^−1^, the transition region is confined to momenta |*k*_*x*_ − *K*| ≤ 0.08 Å^−1^ (Fig. 3a, *t* = 3 fs), corresponding to a Keldysh parameter of *γ* ≤ 0.63. Accounting for scattering and limited momentum resolution, this value is in good agreement with the original LZM formula ${P}_{{\rm{L}}{\rm{Z}}{\rm{M}}}=\exp \left(-2{\rm{\pi }}\frac{\hbar {v}_{{\rm{F}}}}{2|e|{E}_{0}}{|{k}_{x}-K|}^{2}\right)$, which predicts diabatic transitions in a momentum range |*k*_*x*_ − *K*| ≤ 0.04 Å^−1^ (probability 1/e^2^ width; Methods). This confirms that the linearly dispersing Dirac cone of graphene is a perfect realization of the original model Hamiltonian^23^. And even Landau, Zener and Majorana’s assumption that the energy levels intersect at a constant speed holds true, as the strong driving fields propel the electrons past the intersection point in less than 1 fs—much faster than the MIR oscillation period (Methods).

The interplay between intraband motion and LZM tunnelling also explains the modulations of the width observed at twice the driving frequency (Fig. 3c). Because the region close to the K point features the lowest tunnelling barrier, electron injection into the upper part of the Dirac cone becomes most efficient in the few-fs time window between a field extremum and the nearest peak displacement of the pre-existing population, which occurs twice per optical cycle. Scattering shortens this time window, shifting the optimal injection time. This situation should result in a step-like increase of the population in the conduction band, with a phase determined by the interplay of Pauli blocking, the evolution of the field amplitude and scattering (Methods). However, a step-like increase can only be reproduced by simulations using extremely strong dephasing that destroys the quantum memory between successive LZM transitions (Extended Data Fig. 9). For realistic dephasing rates, the step-like character transforms into pronounced 2*ω* oscillations. Their presence in the experimental width of the electron distribution suggests interference of electrons owing to their accumulated phase between individual tunnelling events, emphasizing the role of quantum coherence in light-field-driven devices.

## Ultrafast momentum-resolved relaxation

**Fig. 4:**
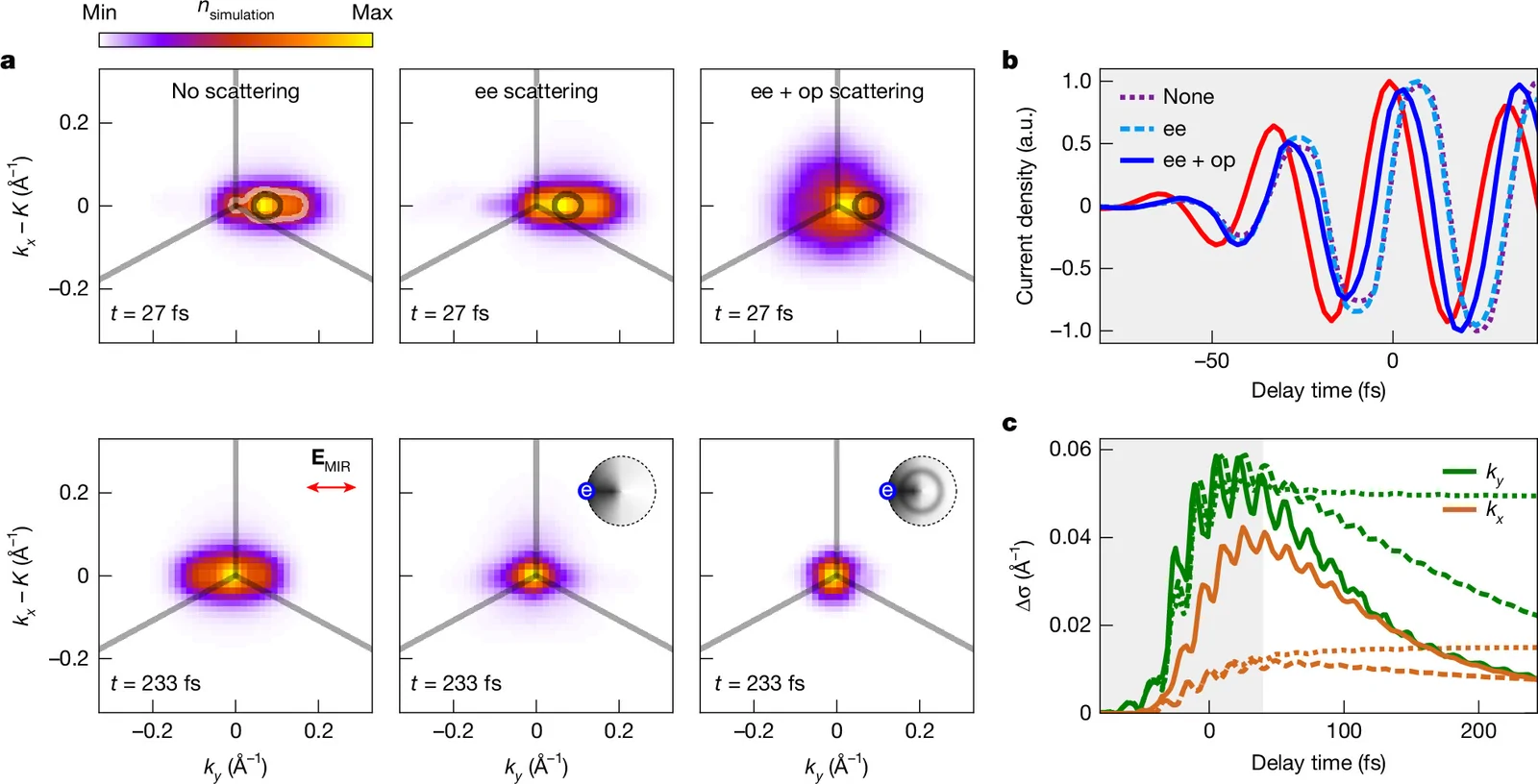
Ultrafast scattering channels in momentum space. **a**, Simulated 2D carrier density, *n*, in the conduction band for selected delay times with different scattering contributions. The leftmost panels show how the carrier distribution behaves for intermediate and late delay times *t*, based on the SBEs without scattering. The light grey line marks the half-maximum contour, highlighting the shape of the carrier distribution. For comparison, the dark grey line shows the corresponding contour when also excluding interband coupling, emphasizing its role in reproducing the experimental observations. It is included in the other panels for reference. The middle and right panels show the carrier distribution including different scattering channels: electron–electron (ee) scattering and electron–electron combined with electron–optical phonon (ee + op) scattering, respectively. The cartoons in the insets illustrate the regions in momentum space accessible for an electron (small, blue sphere) through scattering (dark regions: accessible; bright regions: inaccessible). **b**, Time-dependent evolution of the extracted current for different scattering mechanisms (dotted purple line: no scattering; dotted light blue line: ee scattering; solid blue line: ee + op scattering). The driving field is shown as a red line. Electron–electron scattering affects the phase delay only slightly, whereas including electron–optical phonon scattering affects it markedly. **c**, Temporal evolution of the change in the width of the electron distribution, Δσ, along *k*_*x*_ (brown) and *k*_*y*_ (green) for different levels of theory (line style as in **b**; dotted lines: no scattering and ee scattering; solid lines: ee + op scattering). Without scattering (dotted curves), the distribution is broadened strongly only in the direction of the field. By including scattering with optical phonons (solid curves), it is possible to reach a qualitative agreement with the experiment. a.u., arbitrary units.

Although LZM dynamics dominate at early delay times, scattering limits the time frame for fully coherent light–matter interaction. At *t* = 26.3 fs (Fig. 3a), the initially anisotropic electron distribution broadens also along the *k*_*x*_ direction and approaches a circular shape with a radius of about 0.065 Å^−1^, before it contracts to a more equilibrated and isotropic distribution (*t* = 233 fs). This ultrafast change of the elliptical distribution (Fig. 3c) reflects the interplay between field-driven acceleration, LZM transitions, scattering and carrier relaxation. Our SBE simulations (Extended Data Fig. 10 and Methods) predict distinctive momentum fingerprints for electron–electron and electron–optical phonon scattering. 2D mapping thus allows us to identify their signatures (Fig. 4a). Neglecting all forms of scattering results in a broadening (up to 0.053 Å^−1^) and displacement (about 0.107 Å^−1^) of the carrier density along the *k*_*y*_ direction and the distribution remains elliptical after the MIR pulse (*t* = 233 fs), retaining the momentum hallmark of LZM transitions. Moreover, the phase delay of the microscopic current (Fig. 4b) is approximately π/2, as expected for undamped intraband motion. Introducing electron–electron scattering, the initially strongly broadened and displaced momentum distribution (*t* = 27 fs) partially thermalizes (*t* = 233 fs) and shrinks to a circular shape with a radius of about 0.038 Å^−1^. Still, the peculiar angular dependence of electron–electron scattering^41,47^ retains a large ellipticity on shorter timescales, and the phase delay is only marginally affected. This is caused by the reduced phase space for scattering imposed by the pseudospin texture of graphene.

Simulations including scattering with Γ point longitudinal (LO) and transverse (TO) optical phonons reproduce the experimentally observed dynamics very well. For *t* = 27 fs, the electron distribution shows a substantial broadening in both the *k*_*y*_ (about 0.051 Å^−1^) and the *k*_*x*_ (about 0.041 Å^−1^) directions, associated with an isotropic redistribution of the charge carriers. This leads to a nearly circular distribution whose COM is shifted by only 0.008 Å^−1^ at *t* = 27 fs, much less than in the previous cases. This results from a large change in the phase delay (Fig. 4b), approaching a value of π/4. After the pulse (*t* = 233 fs), the distribution relaxes to a fully circular shape with a radius of approximately 0.031 Å^−1^. Both the phase delay and the time-dependent broadening of the electron distribution (Fig. 4c) are in good qualitative agreement with the experiment. We can, thus, identify the key limiting factor for coherent control dictated by the fastest momentum redistribution channel, which, in graphene, is electron–optical phonon scattering (Fig. 4b, solid blue line).

## Conclusion

Our experiment opens the momentum horizon of subcycle photoemission over the entire first BZ of basically any quantum material. This universal tool enabled us to directly observe LZM transitions in the band structure of a solid. Attosecond-precision videography of graphene unravels how interband LZM tunnelling couples to field-driven intraband acceleration along the Dirac cone and how scattering influences and limits a fully coherent evolution. This platform may soon address other key questions of condensed matter physics and ultrafast information technologies, from optical band-structure engineering and phase transitions to light-field-driven sculpting of molecular orbitals.

## Methods

### Experimental setup

**Extended Data Fig. 1:**
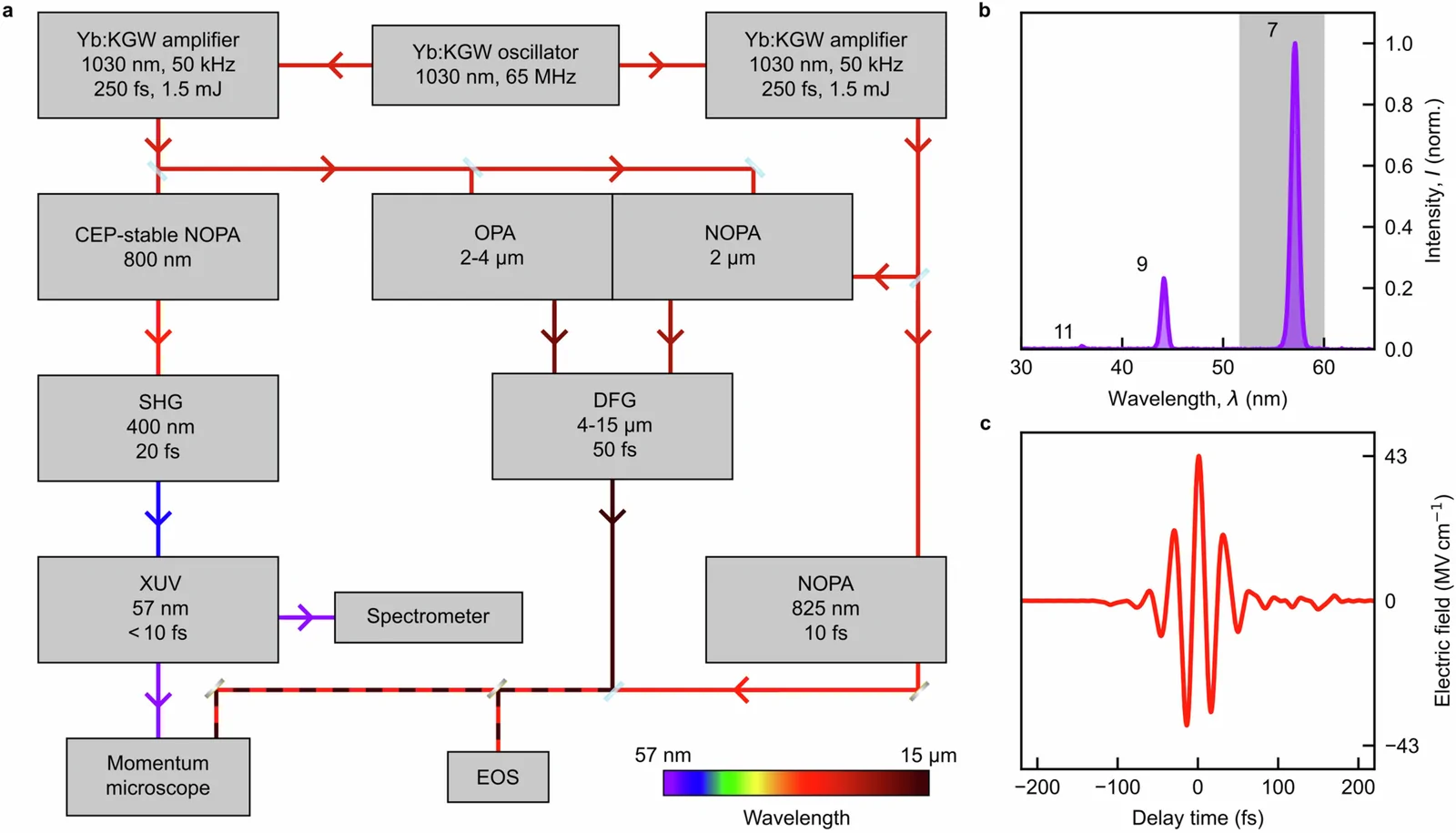
Experimental setup. **a**, A Yb:KGW oscillator seeds two Yb:KGW amplifiers, each providing a pulse energy of 1.5 mJ at a repetition rate of 50 kHz. Half of the output of one amplifier is upconverted to 400-nm pulses with a pulse duration of 20 fs, which are then used to generate sub-10-fs XUV pulses by high-harmonic generation in argon (left side). In parallel, widely tunable, intense, few-cycle and CEP-stable MIR pulses are generated by difference frequency generation (DFG) of the outputs of a dual-branch OPA, which is supplied with the remaining half of the output of the first amplifiers (middle). The output of the second amplifier is used to almost exclusively pump the last amplification stage in the dual-branch OPA. Furthermore, a NOPA generates 10-fs-long NIR pulses (right side), which can be used to detect the MIR waveforms (EOS). Finally, all beams are guided into the UHV chamber of the momentum microscope, in which they are spatially overlapped. **b**, Generated XUV spectrum. The intense seventh harmonic order centred around 57 nm is selected by multilayer mirrors (grey shaded area) and is used as a probe pulse in the subcycle momentum microscopy experiments. **c**, Typical MIR waveform measured by EOS.

The subcycle momentum microscopy setup (Extended Data Fig. 1a) is based on two ytterbium-doped potassium gadolinium tungstate (Yb:KGW) amplifiers (CARBIDE, Light Conversion Inc.). Each amplifier generates near-infrared light pulses with a duration of 250 fs, a centre wavelength of 1,030 nm and a pulse energy of 1.5 mJ, at a repetition rate of 50 kHz. In the probe arm, the second harmonic of the output of a four-stage noncollinear optical-parametric amplifier (NOPA; ORPHEUS-OPCPA, Light Conversion Inc.)^48^ yields carrier-envelope phase (CEP)-stabilized pulses centred at 400 nm with a pulse duration of 20 fs. These pulses are focused into a high-pressure argon gas jet to generate high-order harmonics (Extended Data Fig. 1b) in a vacuum beamline^49^. The seventh harmonic at a photon energy of 21.7 eV is spectrally selected by two multilayer mirrors (Extended Data Fig. 1b, grey shaded area). They feature a bandwidth of 0.9 nm around 57 nm and thus support a Fourier-limited pulse duration of less than 10 fs. The XUV pulses enter the experimental ultrahigh-vacuum (UHV) chamber, the momentum microscope^32^, through a 200-nm-thin aluminium foil, which also filters out the ultraviolet driving pulses.

Because the count rate in a time-of-flight photoemission momentum microscope is limited to only about one electron per laser shot, a high repetition rate is key for keeping measurement times at a reasonable amount while ensuring good statistics. In our experiment, this requirement entails the challenge of pushing the generation of the atomically strong, few-cycle phase-stable MIR pump pulses to new limits for a tabletop setup. Here the generated phase-stable MIR pulses feature, to the best of our knowledge, record-high average powers of up to 1 W and electrical peak field strengths of more than 200 MV cm^−1^. These field transients are obtained by difference frequency generation in a gallium selenide crystal between the outputs of two parallel (N)OPAs (ORPHEUS-MIR, Light Conversion Inc.)^50^. They feature pulse durations down to sub-50 fs and can be tuned between 4 µm and 15 µm. A small portion of the laser fundamental is picked off to pump a home-built single-stage NOPA. It is optimized to generate 10-fs pulses centred around 825 nm, which are used for electro-optic sampling (EOS) of the MIR waveforms. Extended Data Fig. 1c shows a typical MIR transient detected by EOS. A pair of wire-grid polarizers allows us to precisely control the polarization direction and power of the MIR pulses, which are then coupled into the UHV chamber through a diamond window.

The intense MIR pump (*s*-polarized) and the XUV probe (*p*-polarized) are spatially overlapped in the vacuum chamber with a custom-built off-axis parabolic mirror featuring a small aperture for XUV transmission and are focused onto monolayer graphene with an angle of incidence of 70°. A piezo-controlled delay stage in the excitation beam path allows us to set the delay between the MIR and XUV pulses with a temporal precision of 230 as. The emitted photoelectrons are detected by a time-of-flight momentum microscope (lens system: GST mbH; detector: HEX40, RoentDek)^51^. Multihit detection ensures linearity of the signal up to a repetition rate of 20 kHz. The combination of the 21.7-eV probe pulses with the large acceptance angle of the momentum microscope allows access to momentum regions up to ±2.14 Å^−1^, as compared with ±0.16 Å^−1^ in previous subcycle ARPES experiments^8,20^. The energy resolution is limited by the broad spectrum of the probe pulse. The bandwidth of the XUV pulse is tunable, setting a limit for the energy resolution of 180 meV. The momentum resolution is better than 0.01 Å^−1^. The custom-designed UHV chamber is shielded by a μ-metal liner and maintains a base pressure of 2 × 10^−10^ mbar. The samples were measured at room temperature.

Inducing field-driven dynamics in the monolayer requires a field that is parallel to the plane of the monolayer. To maximize the corresponding field component, the MIR pulse is *s*-polarized. The polarization of the XUV pulse is set to be perpendicular to the MIR pulse to prevent crosstalk between their fields that could introduce coherent artefacts effects in the observed dynamics. Finally, the rotation of the sample is chosen such that the MIR-induced momentum streaking in vacuum is minimized at one of the K points. This requires one K point to be at *k*_∥_ = 0, with *k*_∥_ being the momentum direction parallel to the electric field of the MIR pulse. This alignment also minimizes matrix-element-induced asymmetries along *k*_∥_ = 0 in the observed energy and momentum range. Although an asymmetry along the *k*_*x*_ direction might persist, it is rather small at the observed energies. This is because the dark corridor only begins to set in at relatively high excitation energies, which do not occur along the *k*_*x*_ direction in the experiment. This may also explain the slightly larger broadening observed in the simulations in which the matrix element was neglected.

### Graphene samples

For sample preparation, an n-doped 6H-SiC(0001) wafer was used as a substrate for growing an epitaxial monolayer graphene with 0° rotational alignment relative to the substrate lattice (0°-EMLG). The wafer was initially degassed in UHV at 880 °C for 30 min. The temperature was subsequently increased to 1,050 °C and maintained for 30 min under an external flux of Si atoms, resulting in a Si-rich (√3 × √3)R30° surface reconstruction. The sample was then further annealed at 880 °C for an extra 30 min, again under Si flux, resulting in the Si-rich (3 × 3) surface reconstruction. With this procedure, the oxide layer is removed from the SiC surface and atomic cleanness is achieved. In the next step, 0°-EMLG was obtained by annealing the sample at 1,400 °C for 30 min in borazine (B_3_N_3_H_6_) atmosphere with a partial pressure of 1.5 × 10^−6 ^mbar. The borazine molecules act as surfactants on the heated SiC surface. They inhibit the formation of the conventional 30°-rotated graphene layer but instead force the homogeneous graphene layer in an unconventional 0° orientation with respect to the substrate lattice. For more details on the preparation of the 0°-EMLG, see refs. ^38,52^^,^^53^. Following growth, the surface morphology and crystalline structure were characterized using low-energy electron microscopy and low-energy electron diffraction. The average width of terraces on the SiC surface is approximately 160 nm and the surface is fully covered by 0°-EMLG. Subsequently, the sample was shortly exposed to air, transported in static vacuum (0.1 mbar) to the experimental chambers and degassed at 350 °C for 3 h before the measurements. The photoemission experiments reveal that the 0°-EMLG sample is n-doped, with the Fermi level approximately 200 meV above the Dirac point.

### Electric field waveform reconstruction and streaking compensation

The acceleration of photoemitted electrons in vacuum allows for an in situ reconstruction of electric field waveforms^20^. In addition to acceleration within the sample, the *s*-polarized field also accelerates electrons after they have left the material. This causes a momentum change of the entire band structure parallel to the surface of the sample, Δ*k*_∥_, that follows the vector potential of the field. The electric field waveform can thus be calculated by:

$${{\bf{E}}}_{\parallel }(t)=-\frac{\hbar }{e}\frac{{\rm{d}}}{{\rm{d}}t}\Delta {k}_{\parallel }(t).$$

To extract Δ*k*_∥_, the extracted photoemission data are shifted stepwise along *k*_∥_ and compared with a reference spectrum without a field. Within a fixed momentum window centred on the Dirac point, spanning +160 meV to −1.5 eV relative to the Fermi level (a range that, at equilibrium, encompasses predominantly the valence band), the sum of absolute pixel-wise differences between the measured data and the reference is computed at each iteration. The iteration with the smallest overall error gives the value for Δ*k*_∥_. Repeating this procedure for every delay point, *t*, allows a reconstruction of the applied field **E**_∥_(*t*). To refine the retrieved waveform, a second method for extracting Δ*k*_∥_(*t*) is used. Tracking the change in the number of electrons, integrated over all detected energies (+1 eV to −7 eV), with positive or negative momentum along *k*_∥_, yields a unitless quantity directly proportional to Δ*k*_∥_(*t*), which is not subject to discretization caused by the binning of the data along *k*_∥_. The waveform that can be extracted in this way is then scaled by the peak electric field from the first method. The resulting waveform is shown in Fig. 2c.

The extracted Δ*k*_∥_ is also used to compensate for the streaking in the photoemission spectra. As well as the trivial shift along *k*_∥_, a shift in energy, Δ*ε*, is connected to momentum streaking. The energy of every electron in vacuum is given by:

$$\varepsilon ({\bf{k}})=\frac{{\hbar }^{2}}{2m}{{\bf{k}}}^{2}.$$

The change in energy caused by Δ*k*_∥_ can therefore be calculated with:

$$\Delta \varepsilon ({k}_{\parallel })=\varepsilon ({\bf{k}}+\Delta {\bf{k}})-\varepsilon ({\bf{k}})=\frac{{\hbar }^{2}}{m}{k}_{\parallel }\Delta {k}_{\parallel }+\frac{{\hbar }^{2}}{2m}\Delta {k}_{\parallel }^{2}$$

**Extended Data Fig. 2:**
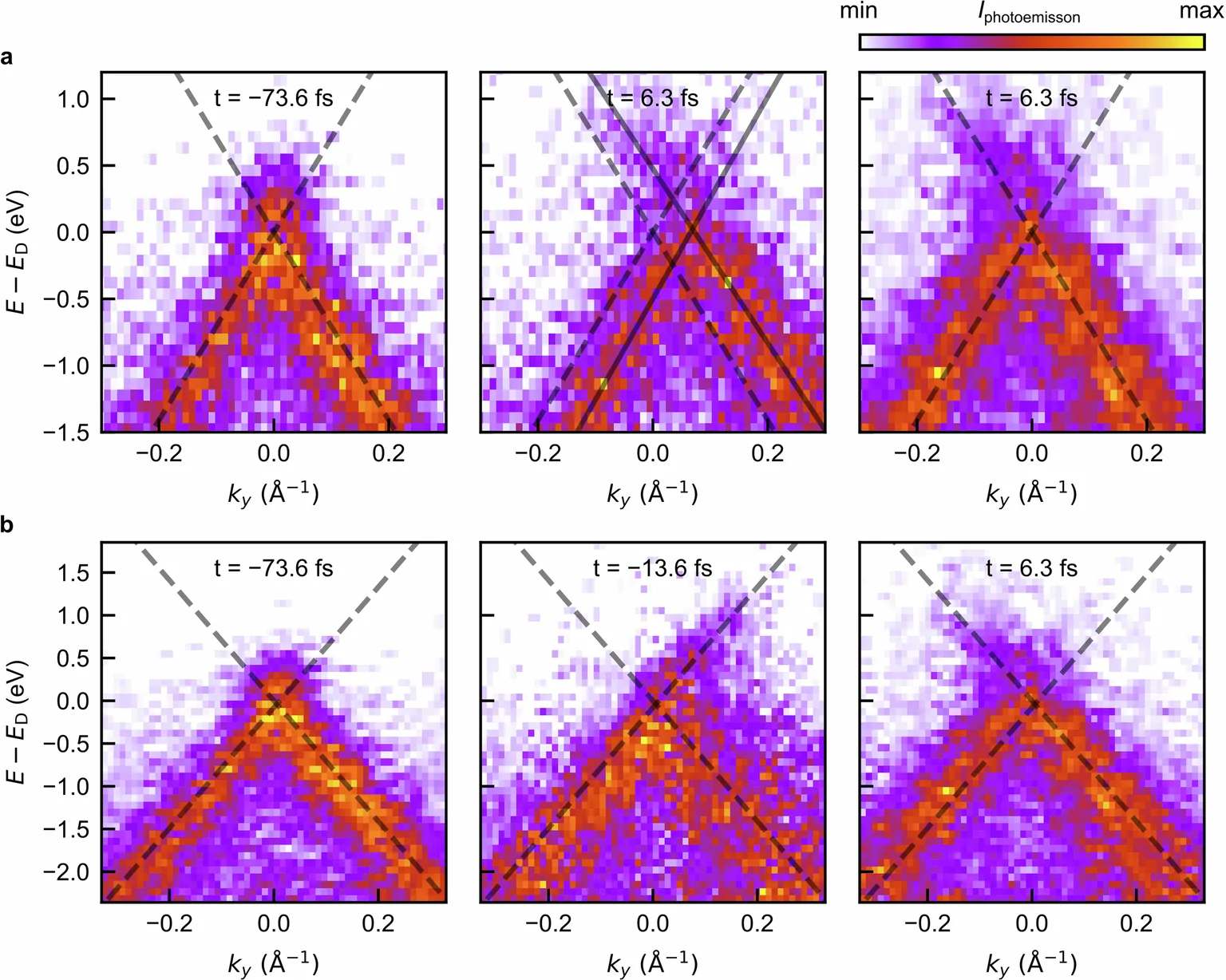
Electric field extraction and streaking compensation. **a**, Photoemission spectra in different streaking conditions. The first two panels show a band-structure snapshot taken in equilibrium (left panel) and a snapshot with strong streaking along *k*_*y*_ by the *s*-polarized MIR field (middle panel). The electric field is reconstructed by matching the streaked band structures to the equilibrium case. Once the electric field is known, the streaking effects can be compensated (right panel). The dashed black lines are a guide to the eye representing the equilibrium band structure. Applying the calculated shifts along energy and momentum, according to the extracted field from the middle panel, to the dashed black line yields the solid black line. **b**, Unfiltered version of the data shown in Fig. 2, showing the raw photoemission data after streaking compensation.

**Extended Data Fig. 3:**
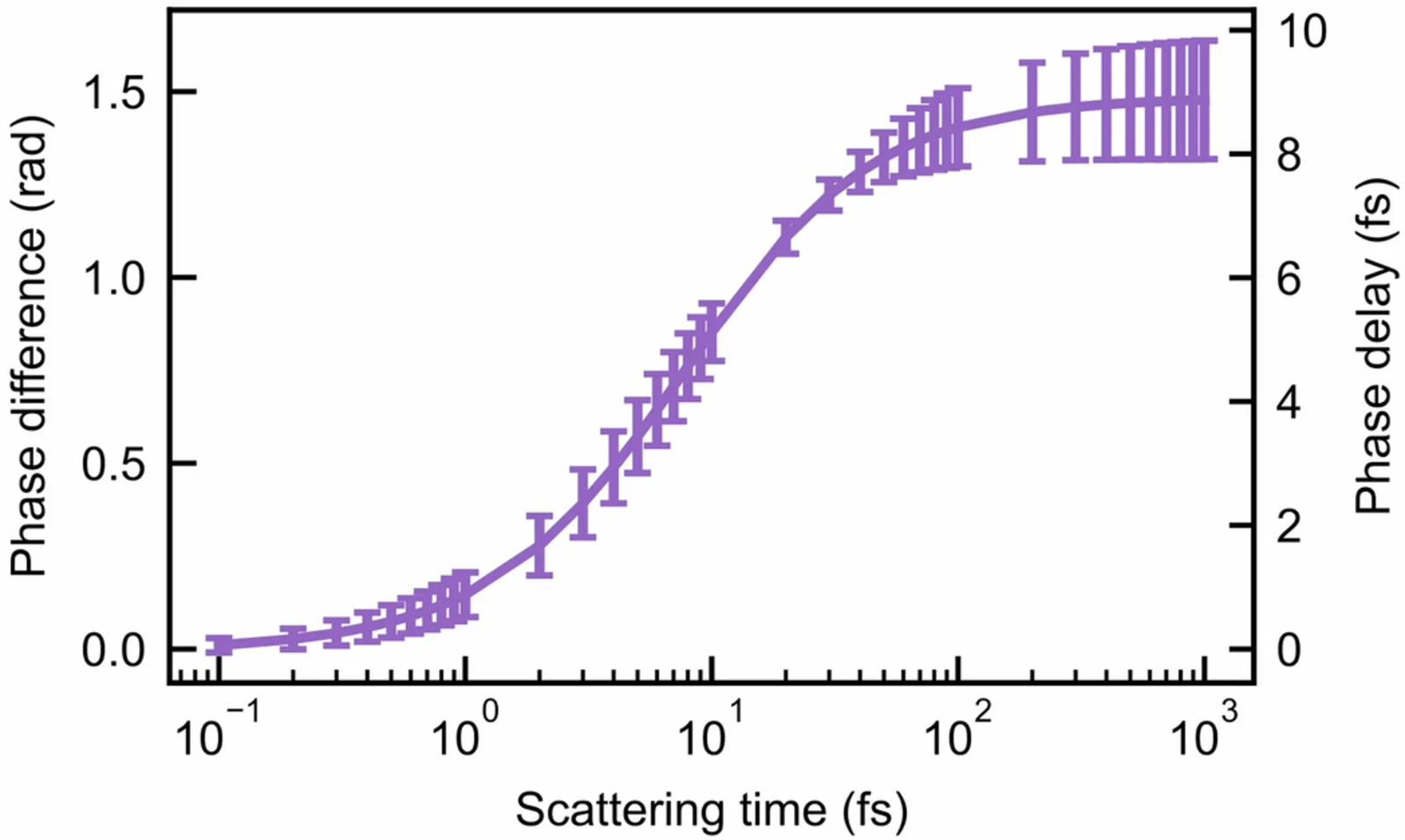
Phase delay in the relaxation time approximation. Simulations based on the SBEs in relaxation time approximation reveal how the phase delay between an applied waveform (central frequency, 30 THz) and the lightwave-driven current scales with the scattering time. For very small scattering times, the phase difference converges to zero, whereas for longer scattering times, it converges to π/2. The strongest change in the phase delay can be observed for scattering times on the order of a half-cycle of the driving waveform.

Extended Data Fig. 2a shows band-structure snapshots taken without an applied field (left panel) and with a strong field applied before streaking compensation (middle panel) and after streaking compensation (right panel). The streaking-induced changes of energy and momentum were compensated for in all photoemission spectra before further analysis of the occupation of the band structure.

### Keldysh parameter in graphene

A parameter that is frequently used to distinguish between the weak-field and strong-field regimes of light–matter interaction is the Keldysh parameter

$$\gamma =\frac{\omega \sqrt{{m}_{{\rm{e}}+{\rm{h}}}^{\ast }\Delta }}{|e|{E}_{0}}.$$

It was introduced by Leonid Keldysh in 1965 as the ratio between the carrier frequency of light *ω* and the tunnelling rate of an electron with mass ${m}_{{\rm{e}}+{\rm{h}}}^{\ast }$ through a barrier Δ, to show that tunnelling and multiphoton ionization in strong electromagnetic fields follow essentially the same physical laws^54^. This ratio scales linearly with frequency *ω* and the inverse of the electric field strength *E*_0_. Tunnelling dominates for *γ* ≪ 1, which holds for sufficiently strong fields and/or low frequencies. Equivalently, it can be expressed as $\gamma =\sqrt{\Delta /{2U}_{{\rm{P}}}}$ by comparing the barrier *Δ* with the mean kinetic energy of a free electron in an oscillating electric field of amplitude *E*_0_, the ponderomotive potential ${2U}_{{\rm{P}}}={e}^{2}{E}_{0}^{2}/4{m}_{e+h}^{\ast }{\omega }^{2}$. Although similar considerations can be made for quasirelativistic motion of electrons in gapless graphene, it is more realistic for the present experiment to take the full Dirac cone with its distribution of barriers and effective masses into account (Extended Data Fig. 7a). In that case, we obtain a *k*_*x*_-dependent Keldysh parameter^26^

$$\gamma =\frac{\hbar |{k}_{x}|\omega }{|e|{E}_{0}}=\frac{\omega \Delta }{2{v}_{{\rm{F}}}|e|{E}_{0}}$$

because both the gap, Δ = 2*ħv*_F_|*k*_*x*_|, and the effective mass at the gap, ${m}_{e+h}^{\ast }=\hbar |{k}_{x}|/2{v}_{{\rm{F}}}$, depend on the distance from the Dirac point. By identifying Δ with the MIR photon energy of 124 meV, we get *γ* = 0.075 for the peak electric fields of 1.57 MV cm^−1^. If we could switch off intraband acceleration, the relevant transition energy from the valence band to the unperturbed conduction band would increase to two times the doping energy of 200 meV, that is, to Δ = 400 meV. Even then, *γ* = 0.24 would comfortably place our measurements in the strong-field regime.

### Current extraction and Fourier analysis of the phase delay

The microscopic intraband current density for each time instant, *j*(*t*), is directly extracted from the individual photoemission measurements, as in Fig. 2c. After compensation of momentum streaking, the photoemission intensity is integrated in two momentum regions with *k*_*y*_ < 0 and *k*_*y*_ > 0, respectively, and normalized by the total number of carriers in the two momentum regions at equilibrium. Subtracting the time-dependent number of counts with positive and negative *k*_*y*_ components, we obtain a dimensionless value proportional to the microscopic current. To reveal the maximum amplitude of the current density, this procedure is performed for all carriers above the Dirac point (*E* − *E*_D_ ≥ 0) within the momentum region |*k*_*x*_ − *K*| < 0.15 Å^−1^. The result is then calibrated to physical values by multiplying with the Fermi velocity and carrier density in the conduction band of graphene with a Fermi level located 200 meV above the Dirac point, which can be computed from the density of states^27^. This yields a maximum current density of *j*(*t*) = 38.2 A cm^−1^. This value is very high, as it is even larger than in previous studies on topological insulators^8,20^, in which the scattering times were two orders of magnitude longer. Given the higher Fermi velocity and field amplitude, a higher current could be expected in the presented experiment. Yet, in a Drude-like regime, this would not be sufficient to compensate for the much higher scattering rate. This confirms that, in ballistic regimes, the current does not follow the Drude model. The same procedure is applied to the results of the simulations based on the SBEs.

To extract the relative timing between the microscopic current in the Dirac cone, *j*(*t*), and the electric field waveform, *E*(*t*), we apply a phase delay analysis. We compute the product between the Fourier transform of the field waveform, $\mathop{E}\limits^{ \sim }(\omega )=F\{E(t)\}$, and the complex conjugate of the Fourier transform of the current, $\mathop{j}\limits^{ \sim }(\omega )=F\{j(t)\}$:

$$P(\omega )=\mathop{E}\limits^{ \sim }(\omega )\times {\mathop{j}\limits^{ \sim }}^{\dagger }(\omega ).$$

The squared modulus of *P*(*ω*) peaks at the frequency of the pump field, whereas its phase corresponds to the timing difference (or phase delay) between the two oscillating quantities. We thus define the average phase difference as:

$$\phi =\frac{\sum _{\omega }|P(\omega )|\times \varphi [P(\omega )]}{\sum _{\omega }|P(\omega )|},$$

in which *φ*[*P*(*ω*)] is the complex phase of *P*(*ω*) and summations only consider frequencies at which |*P*(*ω*)| is larger than 50% of its peak value. To compute the phase delay, *τ*_*i*_, we need to divide this quantity by the central frequency of the pump field. We then obtain the mean phase delay, *τ*_*ϕ*_, and the associated standard deviation of the mean over repeated measurements as:

$${\tau }_{\phi }=\frac{\sum _{i}{\tau }_{i}}{N},\,{\sigma }_{{\tau }_{\phi }}=\frac{\sqrt{\sum _{i}{[{\tau }_{i}-{\tau }_{\phi }]}^{2}}}{N},$$

in which *i* = 1,…, *N* runs over three different measurements performed on this sample geometry.

By performing a gated phase delay analysis, it is also possible to calculate a time-dependent phase difference, *ϕ*(*t*), which indicates the evolution of the scattering processes throughout the duration of the pulse. Here, however, the delay remains relatively stable over the first 100 fs of the driving pulse. We therefore use a single average phase shift, which we associate with a characteristic scattering time. In principle, however, this method could open new avenues for investigating relaxation dynamics across different samples with unprecedented temporal resolution.

### Semiconductor Bloch equations

**Extended Data Fig. 4:**
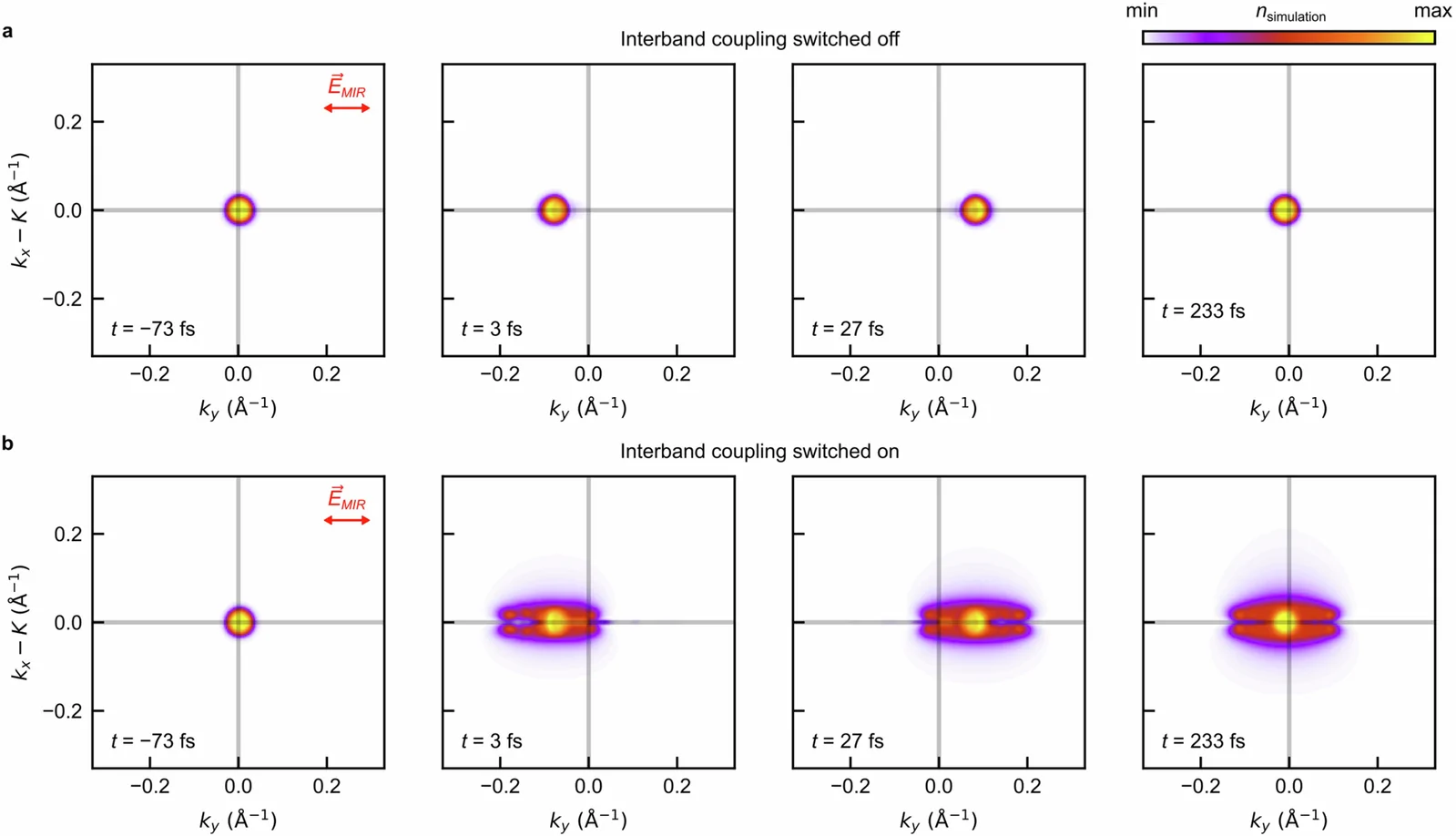
Interband coupling in momentum space. Simulated 2D carrier distribution in the conduction band for selected delay times without scattering. **a**, Interband coupling switched off. **b**, Interband coupling switched on. It causes a large elongation of the carrier distribution along the polarization of the driving light field, filling the gap between the displaced electrons distribution and the K point.

To simulate the time-dependent MIR-driven electron dynamics in graphene, we numerically solve the SBEs^6,55,56^ for a two-band system:

$$\hbar \frac{\partial {f}_{{\rm{e}}}}{\partial t}({\bf{k}},t)=2\hbar {\bf{E}}(t)\cdot {\mathfrak{I}}\{{{\bf{d}}}_{{\rm{h}}{\rm{e}}}({\bf{k}}){p}_{{\rm{e}}{\rm{h}}}({\bf{k}},t)\}-e{\bf{E}}(t)\cdot {\nabla }_{{\bf{k}}}{f}_{{\rm{e}}}({\bf{k}},t)+{\varGamma }_{{\bf{k}}}^{{\rm{e}}}$$

$$\hbar \frac{\partial {f}_{{\rm{h}}}}{\partial t}({\bf{k}},t)=-2\hbar {\bf{E}}(t)\cdot {\mathfrak{I}}\{{{\bf{d}}}_{{\rm{h}}{\rm{e}}}({\bf{k}}){p}_{{\rm{e}}{\rm{h}}}({\bf{k}},t)\}-e{\bf{E}}(t)\cdot {\nabla }_{{\bf{k}}}{f}_{{\rm{h}}}({\bf{k}},t)+{\varGamma }_{{\bf{k}}}^{{\rm{h}}}$$

$$\begin{array}{l}i\hbar \frac{\partial {p}_{{\rm{e}}{\rm{h}}}}{\partial t}({\bf{k}},t)=\left[{\varepsilon }_{{\rm{e}}}({\bf{k}})-{\varepsilon }_{{\rm{h}}}({\bf{k}})-\frac{i\hbar }{{T}_{2}}\right]{p}_{{\rm{e}}{\rm{h}}}({\bf{k}},t)\\ \,+\,{\bf{E}}(t)\cdot {{\bf{d}}}_{{\rm{e}}{\rm{h}}}({\bf{k}})[{f}_{{\rm{e}}}({\bf{k}},t)-{f}_{{\rm{h}}}({\bf{k}},t)]+{\bf{E}}(t)\cdot [{{\bf{d}}}_{{\rm{h}}{\rm{h}}}({\bf{k}})\\ \,-\,{{\bf{d}}}_{{\rm{e}}{\rm{e}}}({\bf{k}})]{p}_{{\rm{e}}{\rm{h}}}({\bf{k}},t)-ie{\bf{E}}(t)\cdot {\nabla }_{{\bf{k}}}{p}_{{\rm{e}}{\rm{h}}}({\bf{k}},t)\end{array}$$

in which **k** is the crystal momentum, *t* is the time, *ħ* the reduced Planck constant and *e* the elementary charge. *f*_e_(**k**, *t*) and *f*_h_(**k**, *t*) are the electron distributions in the conduction and valence bands, respectively, and *p*_eh_(**k**, *t*) is the microscopic interband polarization. In these equations, **E**(*t*) is the MIR electric field, *T*_2_ = 5 fs is the phenomenological dephasing time of the microscopic polarization, *ε*_e_(**k**) and *ε*_h_(**k**) are the energy values of the conduction and valence bands and

$$d({\bf{k}})=\left[\begin{array}{cc}{{\bf{d}}}_{{\rm{e}}{\rm{e}}}({\bf{k}}) & {{\bf{d}}}_{{\rm{e}}{\rm{h}}}({\bf{k}})\\ {{\bf{d}}}_{{\rm{h}}{\rm{e}}}({\bf{k}}) & {{\bf{d}}}_{{\rm{h}}{\rm{h}}}({\bf{k}})\end{array}\right]=e\left[\begin{array}{cc}\langle {\varPsi }_{{\rm{e}}}|i\nabla |{\varPsi }_{{\rm{e}}}\rangle  & \langle {\varPsi }_{{\rm{h}}}|i\nabla |{\varPsi }_{{\rm{e}}}\rangle \\ \langle {\varPsi }_{{\rm{e}}}|i\nabla |{\varPsi }_{{\rm{h}}}\rangle  & \langle {\varPsi }_{{\rm{h}}}|i\nabla |{\varPsi }_{{\rm{h}}}\rangle \end{array}\right]$$

are the dipole moments. ${\varGamma }_{{\bf{k}}}^{{\rm{e}}}$ and ${\varGamma }_{{\bf{k}}}^{{\rm{h}}}$ are the time-dependent and momentum-dependent scattering rates for electrons and holes, respectively, and will be discussed in the next section. In simulations without interband coupling (Fig. 4 and Extended Data Fig. 4), we set ${{\bf{d}}}_{{\rm{eh}}}({\bf{k}})={{\bf{d}}}_{{\rm{he}}}^{\dagger }({\bf{k}})=0$.

In the specific case of graphene, the Hamiltonian of a two-level system can be written as^27^:

$$H({\bf{k}})=\left[\begin{array}{cc}0 & \gamma h({\bf{k}})\\ \gamma {h}^{\ast }({\bf{k}}) & 0\end{array}\right],$$

with

$$h({\bf{k}})=|h({\bf{k}})|{{\rm{e}}}^{{\rm{i}}{\varphi }_{{\bf{k}}}}={{\rm{e}}}^{{\rm{i}}\frac{a{k}_{x}}{\sqrt{3}}}+2{{\rm{e}}}^{-{\rm{i}}\frac{a{k}_{x}}{2\sqrt{3}}}\cos \left(\frac{a{k}_{y}}{2}\right)$$

and *a* = −2.46 Å is the nearest-neighbour lattice distance, *v*_F_ = 1.072 × 10^6^ m s^−1^ is the experimental value of the Fermi velocity and $\gamma =-2\hbar {v}_{{\rm{F}}}/(\sqrt{3}{\rm{a}})$ is the hopping integral. From the Hamiltonian, it is then possible to compute the equilibrium eigenenergies and eigenfunctions:

$$\begin{array}{cc}{\varepsilon }_{{\rm{e}},{\rm{h}}}({\bf{k}})=\pm \gamma |h({\bf{k}})|, & {\varPsi }_{{\rm{e}},{\rm{h}}}({\bf{k}})=\frac{1}{\sqrt{2}}\left[\begin{array}{c}{{\rm{e}}}^{{\rm{i}}{\varphi }_{{\bf{k}}}}\\ \pm 1\end{array}\right].\end{array}$$

The out-of-equilibrium dynamics shown in Extended Data Figs. 4 and 9 were simulated on a 250 × 250 2D square (*k*_*x*_, *k*_*y*_) grid in momentum space. The grid is centred on one of the K points with coordinates *k*_*x*,K_ = 1.703 Å^−1^ and *k*_*y*,K_ = 0 and it has a side length of 0.73 Å^−1^. The simulated time axis ranges from −100 fs to 300 fs, with a step size of 2 fs. The electric field, **E**(*t*), is parallel to *k*_*y*_ and its temporal profile is analytically fitted to the experimentally measured field. At the initial time instant (*t*_0_ = −100 fs), we assume *p*_eh_(**k**, *t*_0_) = 0, whereas the electron distribution corresponds to a Fermi–Dirac distribution. The position of the Fermi level compared with the Dirac crossing is set to *ε*_F_ = 0.2 eV to match the experiment. Simulations comparing the dynamics at other driving frequencies, varying field strengths or different Fermi levels sometimes used analytically modelled waveforms and smaller grid sizes to keep up with increased demands for spatial or temporal resolution. 

### Modelling of the scattering rates

In simulations including scattering, the grid was adjusted to 50 × 50 while maintaining the size in momentum space, to save resources. To reproduce the time-dependent electron distribution observed in momentum space, it is imperative to realistically model the scattering rates:

$$\begin{array}{cc}{\varGamma }_{{\bf{k}}}^{{\rm{e}}}={\frac{\partial {f}_{{\rm{e}}}({\bf{k}},t)}{\partial t}|}_{{\rm{c}}{\rm{o}}{\rm{l}}{\rm{l}}}, & {\varGamma }_{{\bf{k}}}^{{\rm{h}}}={\frac{\partial {f}_{{\rm{h}}}({\bf{k}},t)}{\partial t}|}_{{\rm{c}}{\rm{o}}{\rm{l}}{\rm{l}}}.\end{array}$$

On these timescales, two physical phenomena mainly contribute to the scattering rates:

$${\varGamma }_{{\bf{k}}}^{\alpha }={\varGamma }_{{\bf{k}},{\rm{e}}{\rm{p}}}^{\alpha }+{\varGamma }_{{\bf{k}},{\rm{e}}{\rm{e}}}^{\alpha },\,\alpha \in \{{\rm{e}},{\rm{h}}\},$$

in which ${\varGamma }_{{\bf{k}},{\rm{e}}{\rm{p}}}^{\alpha }$ and ${\varGamma }_{{\bf{k}},{\rm{e}}{\rm{e}}}^{\alpha }$ are, respectively, the electron–phonon and electron–electron scattering rates.

Starting from the electron–phonon interaction, the scattering rate can be written as^57^:

$${\varGamma }_{{\bf{k}},{\rm{e}}{\rm{p}}}^{\alpha }=\sum _{v,{\alpha }^{{\prime} },{{\bf{k}}}^{{\prime} }}\{{S}_{{\alpha }^{{\prime} }\alpha }^{v}({{\bf{k}}}^{{\prime} },{\bf{k}}){f}_{{\alpha }^{{\prime} }}({{\bf{k}}}^{{\prime} },t)[1-{f}_{\alpha }({\bf{k}},t)]-{S}_{\alpha {\alpha }^{{\prime} }}^{v}({\bf{k}},{{\bf{k}}}^{{\prime} }){f}_{\alpha }({\bf{k}},t)[1-{f}_{{\alpha }^{{\prime} }}({{\bf{k}}}^{{\prime} },t)]\},$$

in which *v* is the phonon mode considered and *α*, *α*′ ∈ {e, h} are the band indexes. The first term thus describes transitions from an occupied (**k**′,* α*′) state to an empty (**k**, *α*) state owing to the interaction with a *v* phonon, whereas the second term describes the opposite process. Whereas in the former the state (**k**, *α*) is empty and becomes occupied (thus, the rate is positive), in the latter, the rate must be negative. The probability of these transitions is associated with the electron–phonon transition rate:

$$\begin{array}{c}{S}_{{\alpha }^{{\prime} }\alpha }^{v}({{\bf{k}}}^{{\prime} },{\bf{k}})=\frac{2{\rm{\pi }}}{\hbar }{|{G}_{{\alpha }^{{\prime} }\alpha }^{v}({{\bf{k}}}^{{\prime} },{\bf{k}})|}^{2}\\ \cdot \{({n}_{{\bf{q}}}^{v}+1)\delta [{\varepsilon }_{\alpha }({\bf{k}})-{\varepsilon }_{{\alpha }^{{\prime} }}({{\bf{k}}}^{{\prime} })+\hbar {\omega }_{{\bf{q}}}^{v}]{\delta }_{{{\bf{k}}}^{{\prime} }={\bf{k}}-{\bf{q}}}+{n}_{{\bf{q}}}^{v}\delta [{\varepsilon }_{\alpha }({\bf{k}})-{\varepsilon }_{{\alpha }^{{\prime} }}({{\bf{k}}}^{{\prime} })-\hbar {\omega }_{{\bf{q}}}^{v}]{\delta }_{{{\bf{k}}}^{{\prime} }={\bf{k}}+{\bf{q}}}\}\end{array},$$

in which ${G}_{{\alpha }^{{\prime} }\alpha }^{v}({{\bf{k}}}^{{\prime} },{\bf{k}})$ is the electron–phonon coupling constant, *δ* is the Kronecker delta imposing energy and momentum conservation, **q** and ${\omega }_{{\bf{q}}}^{v}$ are the momentum and angular frequency of a *v* phonon, respectively, and ${n}_{{\bf{q}}}^{v}$ is the number of *v* phonons with a given frequency and momentum, which is described by the Bose–Einstein distribution.

To simplify our description, we assume that: (1) Umklapp processes are negligible and (2) the number of phonons ${n}_{{\bf{q}}}^{v}$ is constant and equal to its value at equilibrium (phonon bath). Moreover, previous works explained the dominating contribution of LO and TO phonons^34,47^. We will thus limit our model to LO and TO phonons and, because they have similar phonon energies (${\omega }_{{\bf{q}}}^{{\rm{LO}}}\simeq {\omega }_{{\bf{q}}}^{{\rm{TO}}}\simeq 164.6\,{\hbar }^{-1}\,{\rm{meV}}$) and matrix elements but their angular dependencies compensate each other, we will treat them together with an effective electron–optical phonon coupling constant^57^:

$${|{G}_{{\alpha }^{{\prime} }\alpha }^{{\rm{LO}}}({{\bf{k}}}^{{\prime} },{\bf{k}})+{G}_{{\alpha }^{{\prime} }\alpha }^{{\rm{TO}}}({{\bf{k}}}^{{\prime} },{\bf{k}})|}^{2}={|{G}_{{\alpha }^{{\prime} }\alpha }^{{\rm{OP}}}|}^{2}=1.542\,{{\rm{eV}}}^{2}.$$

The effective matrix element is constant in momentum space and its value is adjusted to reproduce the experimental data. Owing to the isotropic angular dependence of this term, electron–optical phonon scattering will lead to a rapid spreading of the electron distribution around the Dirac cone.

In the same spirit, the scattering rate for electron–electron interaction can be written as^57^:

$${\varGamma }_{{\bf{k}},{\rm{e}}{\rm{e}}}^{\alpha }=\sum _{{{\bf{k}}}_{\ast },{{\bf{k}}}^{{\prime} },{{\bf{k}}}_{\ast }^{{\prime} }}\sum _{{\alpha }^{{\prime} },\beta ,{\beta }^{{\prime} }}\{{S}_{{\rm{e}}{\rm{e}}}({{\bf{k}}}^{{\prime} },{{\bf{k}}}_{\ast }^{{\prime} },{\bf{k}},{{\bf{k}}}_{\ast }){f}_{{\alpha }^{{\prime} }}({{\bf{k}}}^{{\prime} }){f}_{{\beta }^{{\prime} }}({{\bf{k}}}_{\ast }^{{\prime} })[1-{f}_{\alpha }({\bf{k}})][1-{f}_{\beta }({{\bf{k}}}_{\ast })]-{S}_{{\rm{e}}{\rm{e}}}({\bf{k}},{{\bf{k}}}_{\ast },{{\bf{k}}}^{{\prime} },{{\bf{k}}}_{\ast }^{{\prime} }){f}_{\alpha }({\bf{k}}){f}_{\beta }({{\bf{k}}}_{\ast })[1-{f}_{{\alpha }^{{\prime} }}({{\bf{k}}}^{{\prime} })][1-{f}_{{\beta }^{{\prime} }}({{\bf{k}}}_{\ast }^{{\prime} })]\}{\delta }_{{\bf{k}}+{{\bf{k}}}_{\ast }={{\bf{k}}}^{{\prime} }+{{\bf{k}}}_{\ast }^{{\prime} }},$$

in which $\alpha ,{\alpha }^{{\prime} },\beta ,{\beta }^{{\prime} }\in \{{\rm{e}},{\rm{h}}\}$ are the band indices. The first term, therefore, describes the scattering between a pair of particles in the initial states (*α*′, **k**′) and $({\beta }^{{\prime} },{{\bf{k}}}_{\ast }^{{\prime} })$ to the final states (*α*, **k**) and $(\beta ,{{\bf{k}}}_{\ast })$, and the second term refers to the opposite process. The associated transition rate can thus be written as^57^:

$${S}_{{\rm{e}}{\rm{e}}}({\bf{k}},{{\bf{k}}}_{\ast },{{\bf{k}}}^{{\prime} },{{\bf{k}}}_{\ast }^{{\prime} })={\left(\frac{2{\rm{\pi }}}{\hbar }\right)}^{2}{|M|}^{2}\delta [{\varepsilon }_{{\beta }^{{\prime} }}({{\bf{k}}}_{\ast }^{{\prime} })+{\varepsilon }_{{\alpha }^{{\prime} }}({{\bf{k}}}^{{\prime} })-{\varepsilon }_{\beta }({{\bf{k}}}_{\ast })-{\varepsilon }_{\alpha }({\bf{k}})],$$

in which the matrix element is:

$${|M|}^{2}=\frac{1}{2}[{|V(q)|}^{2}+{|V({q}^{{\prime} })|}^{2}-V(q)V({q}^{{\prime} })]$$

and *q* = |**k** − **k**′| and ${q}^{{\prime} }=|{\bf{k}}-{{\bf{k}}}_{\ast }^{{\prime} }|$. The Coulomb potential between electron pairs is:

$$V(q)=\frac{2{\rm{\pi }}{e}^{2}{V}_{{\rm{e}}{\rm{f}}{\rm{f}}}}{{\epsilon }(q)qA}\frac{1+\cos {\phi }_{{\bf{k}}{{\bf{k}}}^{{\prime} }}}{2}\frac{1+\cos {\phi }_{{{\bf{k}}}_{\ast }{{\bf{k}}}_{\ast }^{{\prime} }}}{2}$$

$$V(q{\prime} )=\frac{2{\rm{\pi }}{e}^{2}{V}_{{\rm{eff}}}}{{\epsilon }({q}^{{\prime} }){q}^{{\prime} }A}\frac{1+\cos {\phi }_{{\bf{k}}{{\bf{k}}}_{\ast }^{{\prime} }}}{2}\frac{1+\cos {\phi }_{{{\bf{k}}}_{\ast }{{\bf{k}}}^{{\prime} }}}{2}$$

with *A* = 4π^2^/Δ*k*^2^. The screening of the Coulomb interaction is given by the dielectric function ${\epsilon }(q)=1+{v}_{{\rm{c}}}(q)D({\varepsilon }_{{\rm{F}}})\mathop{\Pi }\limits^{ \sim }(q)$^57,58^, in which:

$${v}_{{\rm{c}}}(q)=\frac{2{\rm{\pi }}{e}^{2}}{\kappa q},\,\kappa =\frac{{e}^{2}}{{r}_{{\rm{S}}}\hbar {v}_{{\rm{F}}}},\,{r}_{{\rm{S}}}=\frac{\hbar {v}_{{\rm{F}}}}{{\varepsilon }_{{\rm{F}}}},\,D(\varepsilon )=\frac{|\varepsilon |}{2{\rm{\pi }}{\hbar }^{2}{v}_{{\rm{F}}}^{2}}$$

$$\mathop{\Pi }\limits^{ \sim }(q)=\left\{\begin{array}{cc}1, & q < 2{k}_{{\rm{F}}}\\ 1+\frac{{\rm{\pi }}q}{8{k}_{{\rm{F}}}}-\frac{\sqrt{{q}^{2}-4{k}_{{\rm{F}}}^{2}}}{2q}-\frac{q}{4{k}_{{\rm{F}}}}\arcsin \left(\frac{2{k}_{{\rm{F}}}}{q}\right), & {\rm{o}}{\rm{t}}{\rm{h}}{\rm{e}}{\rm{r}}{\rm{w}}{\rm{i}}{\rm{s}}{\rm{e}}\end{array}\right.$$

and *r*_S_ is the Wigner–Seitz radius of graphene, *k*_F_ = *ε*_F_/(*ħv*_F_) the Fermi wavevector and *D*(*ε*) the density of states close to the Dirac point. This approximation holds for *n* ≥ *n*_th_ = 10^12^ cm^−2^, thus if *ε*_F_ ≥ 37.14 meV, as in our case.

In this form, the scattering rate for electron–electron interaction is computationally extremely expensive. Therefore, we assume that the electron distribution for the second particle can be approximated by the equilibrium Fermi–Dirac distribution of the carriers:

$${f}_{\beta }({{\bf{k}}}_{\ast })\simeq {f}_{\beta }^{(0)}({{\bf{k}}}_{\ast }),\,{f}_{{\beta }^{{\prime} }}({{\bf{k}}}_{\ast }^{{\prime} })\simeq {f}_{{\beta }^{{\prime} }}^{(0)}({{\bf{k}}}_{\ast }^{{\prime} }).$$

This allows us to write a simplified expression for the electron–electron scattering rate:

$$\begin{array}{c}{\varGamma }_{{\bf{k}},{\rm{e}}{\rm{e}}}^{\alpha }\simeq \sum _{{{\bf{k}}}^{{\prime} }}\sum _{{\alpha }^{{\prime} },\beta ,{\beta }^{{\prime} }}\{{S}_{{\rm{e}}{\rm{e}},\beta {\beta }^{{\prime} }}^{{\rm{e}}{\rm{f}}{\rm{f}},1}({\bf{k}},{{\bf{k}}}^{{\prime} }){f}_{{\alpha }^{{\prime} }}({{\bf{k}}}^{{\prime} })[1-{f}_{\alpha }({\bf{k}})]\\ \,-\,{S}_{{\rm{e}}{\rm{e}},\beta {\beta }^{{\prime} }}^{{\rm{e}}{\rm{f}}{\rm{f}},2}({\bf{k}},{{\bf{k}}}^{{\prime} }){f}_{\alpha }({\bf{k}})[1-{f}_{{\alpha }^{{\prime} }}({{\bf{k}}}^{{\prime} })]\},\end{array}$$

in which we use that ${S}_{{\rm{ee}}}({\bf{k}},{{\bf{k}}}_{\ast },{{\bf{k}}}^{{\prime} },{{\bf{k}}}_{\ast }^{{\prime} })={S}_{{\rm{ee}}}({{\bf{k}}}^{{\prime} },{{\bf{k}}}_{\ast }^{{\prime} },{\bf{k}},{{\bf{k}}}_{\ast })$ and define:

$${S}_{{\rm{ee}},\beta {\beta }^{{\prime} }}^{{\rm{eff}},1}({\bf{k}},{{\bf{k}}}^{{\prime} })=\sum _{{{\bf{k}}}_{\ast },{{\bf{k}}}_{\ast }^{{\prime} }}{S}_{{\rm{ee}}}({\bf{k}},{{\bf{k}}}_{\ast },{{\bf{k}}}^{{\prime} },{{\bf{k}}}_{\ast }^{{\prime} }){f}_{{\beta }^{{\prime} }}^{(0)}({{\bf{k}}}_{\ast }^{{\prime} })[1-{f}_{\beta }^{(0)}({{\bf{k}}}_{\ast })]{\delta }_{{\bf{k}}+{{\bf{k}}}_{\ast }={{\bf{k}}}^{{\prime} }+{{\bf{k}}}_{\ast }^{{\prime} }}$$

$${S}_{{\rm{ee}},\beta {\beta }^{{\prime} }}^{{\rm{eff}},2}({\bf{k}},{{\bf{k}}}^{{\prime} })=\sum _{{{\bf{k}}}_{\ast },{{\bf{k}}}_{\ast }^{{\prime} }}{S}_{{\rm{ee}}}({\bf{k}},{{\bf{k}}}_{\ast },{{\bf{k}}}^{{\prime} },{{\bf{k}}}_{\ast }^{{\prime} }){f}_{\beta }^{(0)}({{\bf{k}}}_{\ast })[1-{f}_{{\beta }^{{\prime} }}^{(0)}({{\bf{k}}}_{\ast }^{{\prime} })]{\delta }_{{\bf{k}}+{{\bf{k}}}_{\ast }={{\bf{k}}}^{{\prime} }+{{\bf{k}}}_{\ast }^{{\prime} }}.$$

In our simulations, we only focus on a small momentum space region around one of the six equivalent K points. In doing so, we neglect intervalley electron–phonon and electron–electron scattering processes, which are not expected to contribute notably in these conditions^34^. However, for electron–electron scattering, it is possible that an electron scatters from one state to another within the same valley by interacting with a charge carrier in a different one. For this reason, we effectively account for these processes by multiplying ${S}_{{\rm{ee}},\beta \beta {\rm{{\prime} }}}^{{\rm{eff}},1}$ and ${S}_{{\rm{ee}},\beta \beta {\rm{{\prime} }}}^{{\rm{eff}},2}$ by the number of equivalent K valleys, that is, six. To reproduce the experimental data, we assumed *V*_eff_ = 4.386. It is important to notice that the dependence on the relative angle between **k**, **k**′, ${{\bf{k}}}_{\ast }$ and ${{\bf{k}}}_{\ast }^{{\prime} }$ determines the characteristic angular dependence of electron–electron scattering in graphene^47^, also termed collinear scattering^41^. Notably, at variance with the relaxation time approximation, our approach does not enforce any specific electron distribution at any time instant, while matching our experimental results.

### Relaxation time approximation

To reduce the computational costs when relating the phase delay to an effective scattering time, a customary approach when solving the SBEs is to invoke the relaxation time approximation^6,20,55,56^. In this context, the scattering rate ${\varGamma }_{{\bf{k}}}^{\alpha }$ is assumed to be inversely proportional to a time constant, *T*_1_, associated with the mean free time between scattering events. Defining ${f}_{\alpha }^{(0)}({\bf{k}})$ as the equilibrium electron distribution in the *α* band, we obtain:

$${\varGamma }_{{\bf{k}}}^{\alpha }={\frac{\partial {f}_{\alpha }({\bf{k}},t)}{\partial t}|}_{{\rm{c}}{\rm{o}}{\rm{l}}{\rm{l}}}=-\frac{{f}_{\alpha }({\bf{k}},t)-{f}_{\alpha }^{(0)}({\bf{k}})}{{T}_{1}}.$$

It is important to notice that, even though this approach allows us to extract an effective time constant, it is not enough to reproduce the experimental data. In fact, in this approximation, the scattering rate at a given point **k** in momentum space will only depend on the instantaneous electron distribution at that point and not on any other point in momentum space. This makes it impossible to reproduce any carrier redistribution dynamics in momentum space owing to, for example, the interaction with optical phonons.

### LZM transition region

The original works of Landau^22^, Zener^23^, Majorana^24^ and Stückelberg^36^ considered a two-state quantum system with an energy separation Δ*ε* = *ε*_1_(*t*) − *ε*_2_(*t*) = *αt* that changes linearly with time and undergoes an avoided crossing with a half gap energy *δ*. The diabatic transition probability is then given by^26^

$${P}_{{\rm{L}}{\rm{Z}}{\rm{M}}}=\exp (-2{\rm{\pi }}\varGamma )=\exp \left(-2{\rm{\pi }}\frac{{\delta }^{2}}{\hbar \alpha }\right)$$

with the diabaticity parameter $\varGamma =\frac{{\delta }^{2}}{\hbar \alpha }$. This simple formula can be used to estimate the region in momentum space in which transitions occur in our experiment. The model is applicable under the condition that the characteristic sweep time through the transition, *τ* = 2*δ*/*ε*, is short compared with the oscillation period *T* = 2π/*ω* of the driving electric field. In this case, the field can be considered quasistatic during the transition and we simply have $\alpha =2{v}_{{\rm{F}}}\hbar |{k}_{x}-K|=2{v}_{{\rm{F}}}|e|{E}_{0}$. With *δ* = *v*_F_*ħ*|*k*_*x*_ − *K*|, we obtain $\tau =\frac{\hbar | {k}_{x}-K|}{|e|{E}_{0}}=\frac{\gamma }{\omega }$. Because *γ* ≈ 0.1, the crossing indeed occurs very fast (*τ* < 1 fs). For the peak electric field *E*_0_ = 1.57 MV cm^−1^, the diabaticity parameter $\varGamma =\frac{\hslash {v}_{{\rm{F}}}}{2|e|{E}_{0}}{|{k}_{x}-K|}^{2}\,\cong$ $210\,{{\rm{\mathring{\mathrm{A}} }}}^{2}{|{k}_{x}-K|}^{2}$ becomes smaller than 1/π for |*k*_*x*_ − *K*| < 0.04 Å^−2^. In this region around the Dirac point, 1 − e^−2^ = 86% of the transitions from the valence band to the conductions band are expected to take place as long as Pauli blocking can be neglected. This region roughly coincides with the momentum area in which the conduction band of our doped graphene samples is occupied. This shows that transitions can only take place after the electrons have been accelerated away from the Dirac point. After several driving cycles, the observed dynamics of the doped and a hypothetical undoped sample are expected to be similar (Extended Data Fig. 8).

### Injection time analysis

**Extended Data Fig. 5:**
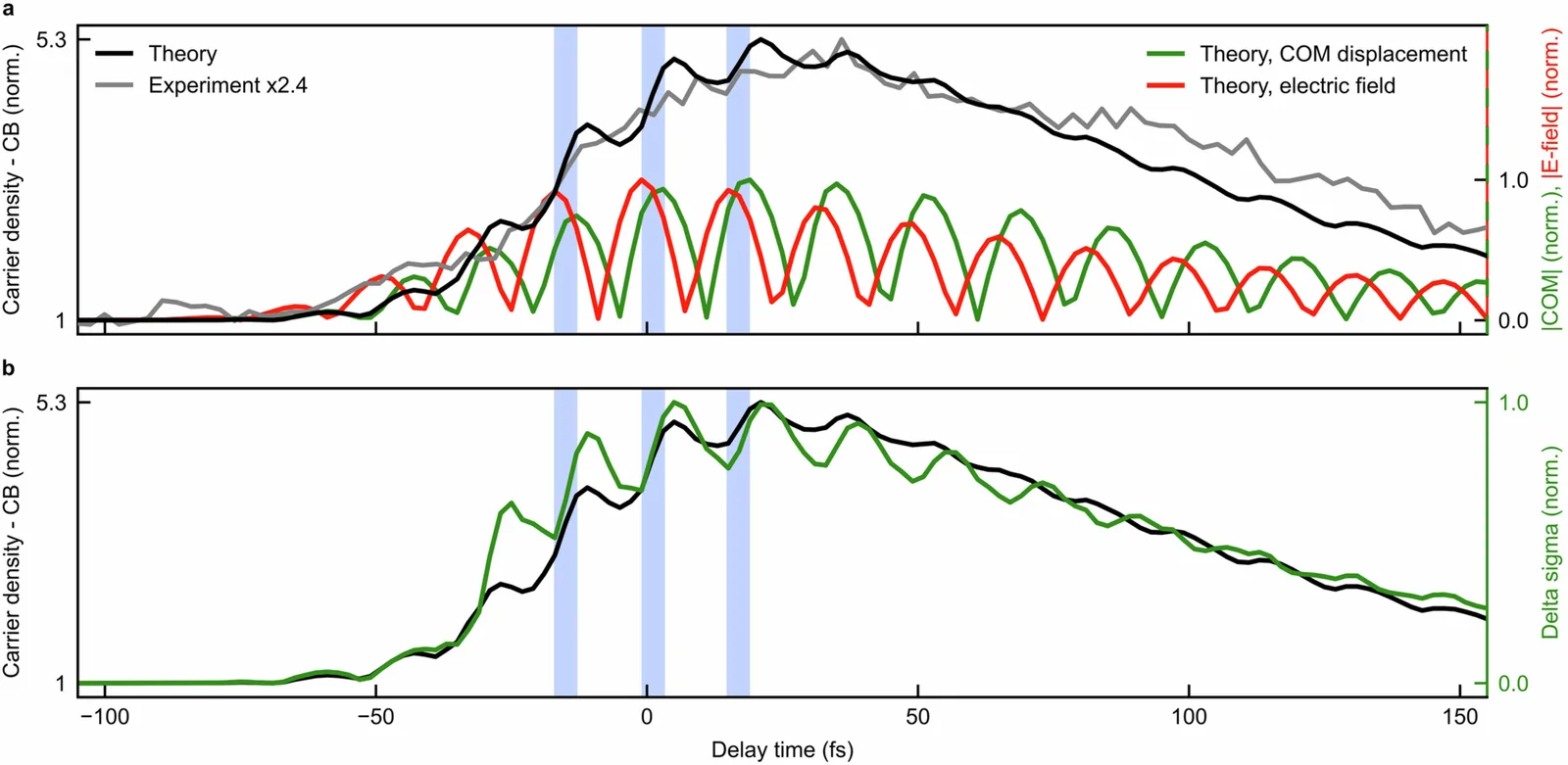
Population dynamics in the conduction band. **a**, Normalized carrier density in the conduction band extracted from our theory model with electron–phonon and electron–electron scattering (black curve) and from experimental data (grey curve). The observed difference in the number of extra carriers between theory and experiment is to be expected, as the theory does not account for inherent constraints of the measurement itself, such as matrix element effects. The population rises fastest during the time window between a maximum of the field (red curve) and the COM displacement (green curve). **b**, During the first few cycles, the evolution of the width of the electron distribution (green curve) closely follows the population dynamics (black curve).

**Extended Data Fig. 6:**
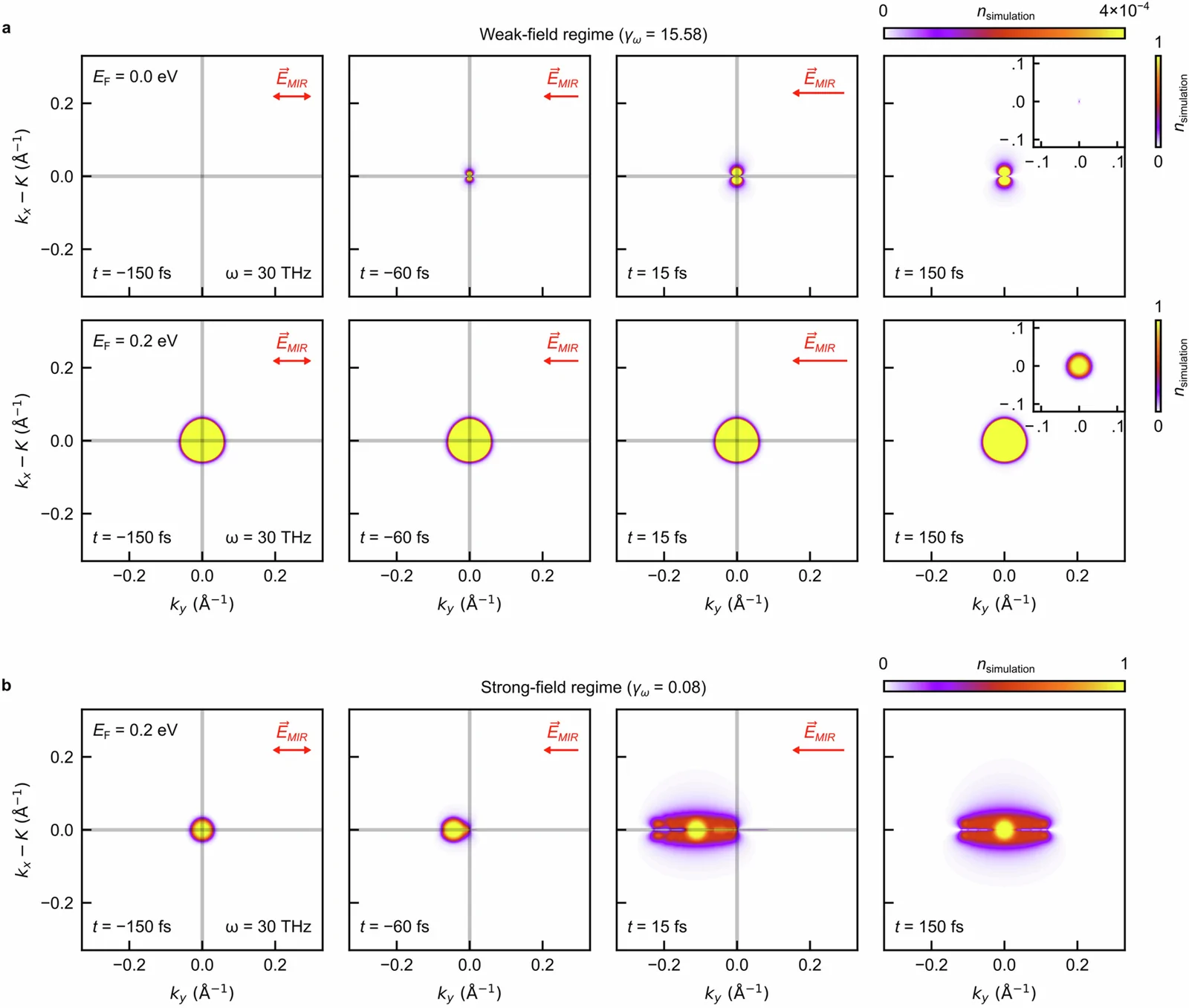
Weak-field and strong-field regimes in momentum space. Simulated 2D carrier distribution in the conduction band for selected delay times without scattering in the weak-field and strong-field regimes. The interaction regime is defined by means of the Keldysh parameter for an energy gap corresponding to single-photon absorption. **a**, The weak-field regime is reached by using fields that are 200 times weaker than in the experiment. Here the interaction is dominated by perturbative absorption, which is strongly affected by the selection rules owing to the pseudospin texture of graphene. By removing the doping, the first row highlights how this leads to a population increase in regions perpendicular to the polarization of the light pulse. The second row depicts the situation with the same doping level as in the experiment. Here no change in the population can be resolved, even when exaggerating the colour map by a factor of 2,500. **b**, In the strong-field regime, the interaction with light is characterized by a unidirectional elongation of the electron distribution parallel to the electric field as well as a displacement of the Fermi surface.

**Extended Data Fig. 7:**
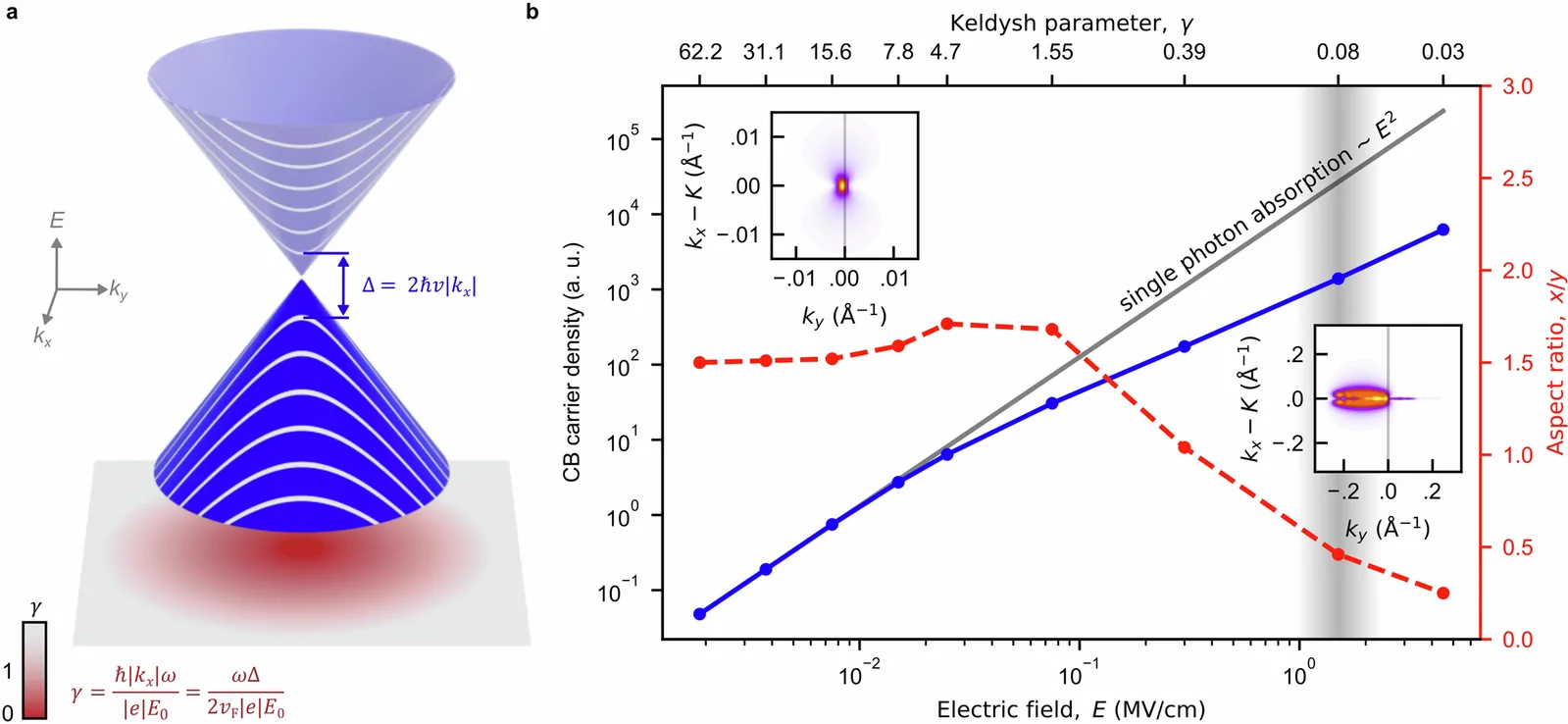
Population-transfer scaling in the weak-field and strong-field regimes. **a**, Illustration of the distribution of bandgaps Δ and the varying Keldysh parameter *γ* in graphene. **b**, Simulated 2D change of the population in the conduction band (blue curve) and aspect ratio of the carrier distribution (red curve) after interacting with the electric field for different field strengths in an undoped sample without scattering. For Keldysh parameters *γ* ≫ 1, the number of excited carriers scales quadratically with the field (grey curve), indicative of single carrier absorption. At the same time, the aspect ratio of the carrier distribution shows that the distribution is broader along the momentum direction perpendicular to the light field (left inset). In the strong-field regime (*γ* ≪ 1), the number of excited carriers is trending towards saturation, indicative of light-field-induced tunnelling as the transition mechanism. Simultaneously, the aspect ratio of the carrier distribution inverts, resulting in the elliptical distribution observed in the experiment with broadening in the direction parallel to the applied field (right inset). The extracted sub-quadratic scaling of the excited carrier density for the field strength used in the experiment (grey shaded area) indicates that perturbative single-photon or multiphoton absorption does not play a substantial role in the interaction.

**Extended Data Fig. 8:**
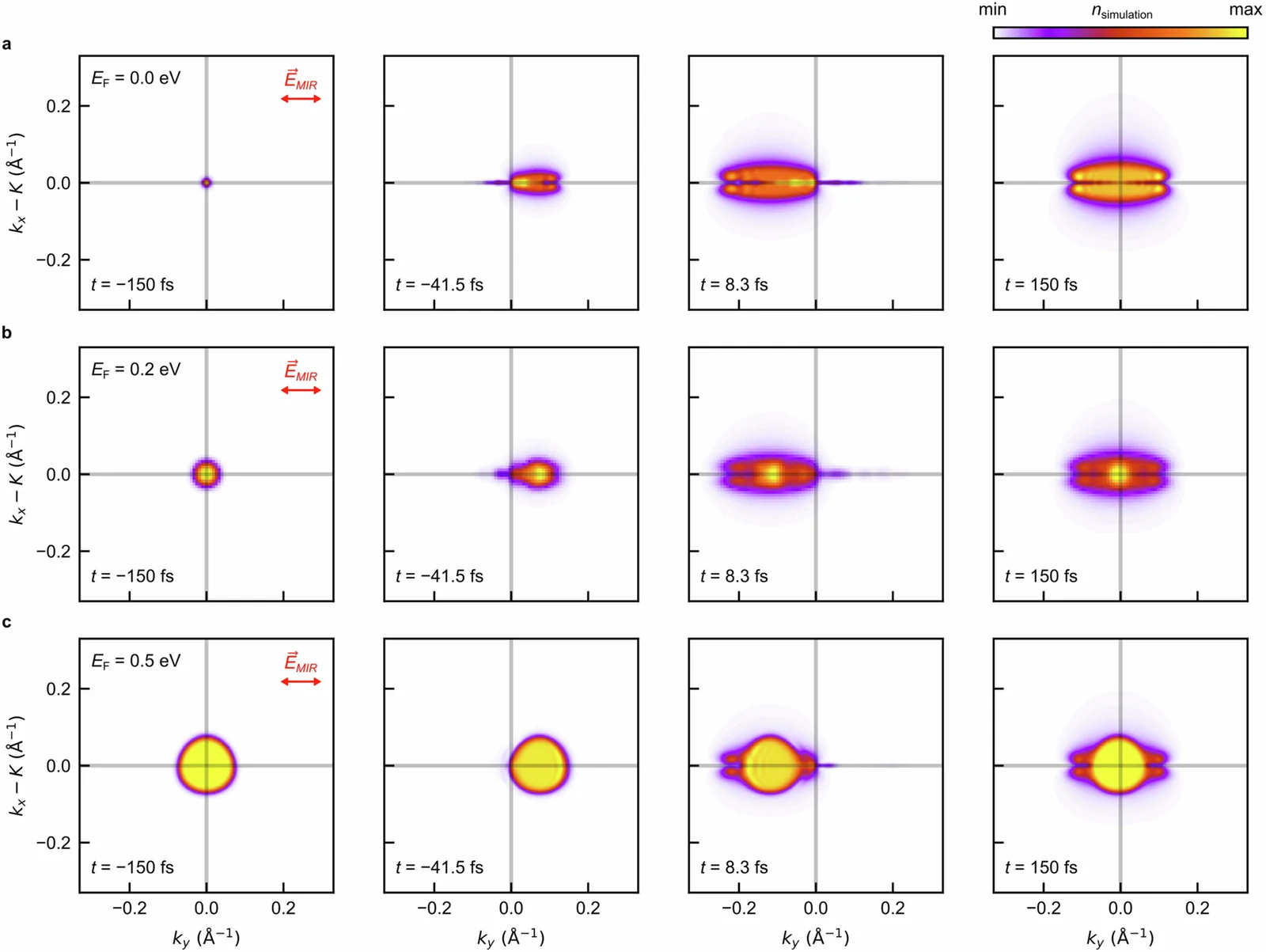
Effect of doping in momentum space. Simulated 2D carrier distribution in the conduction band for selected delay times without scattering and different doping levels. **a**, No doping. **b**, Moderate doping. **c**, High doping. Intrinsic (**a**) and the moderately doped graphene (**b**, Fermi level 200 meV) closely resemble each other. Higher doping levels (**c**) reveal the difference between the intraband and interband signatures.

**Extended Data Fig. 9:**
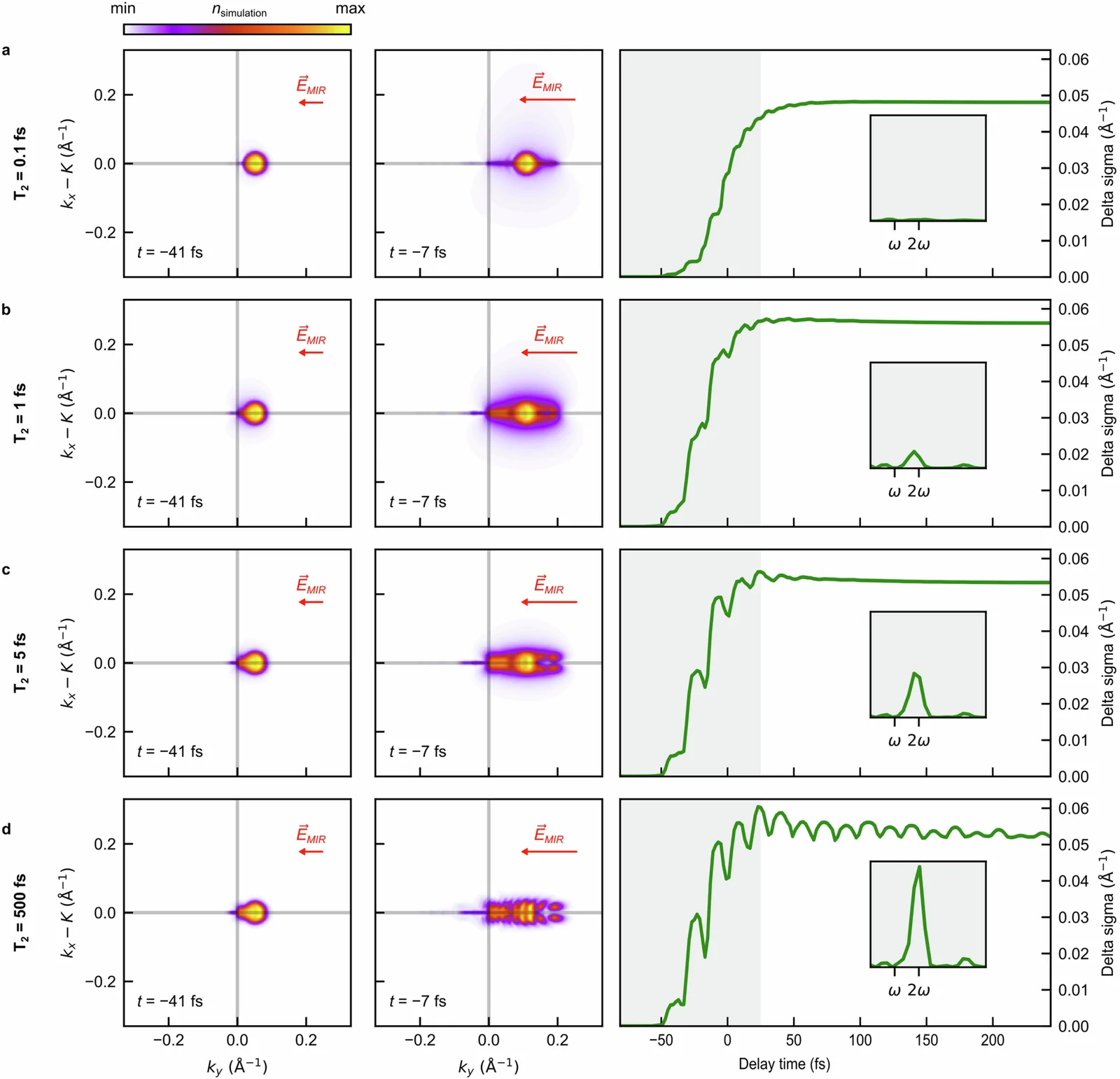
Effect of dephasing in momentum space. Simulated 2D carrier distribution in the conduction band for different dephasing times. **a**, *T*_2_ = 0.1 fs. **b**, *T*_2_ = 1 fs **c**, *T*_2_ = 5 fs. **d**, *T*_2_ = 500 fs. The 2D carrier distribution is shown after the first (left panel) and second (middle panel) cycles. For very fast dephasing, the efficiency for LZM transitions decreases substantially, manifesting in a lack of additional carriers in the second panel of **a**. For very slow dephasing, signatures of LZSM interference emerge after the second cycle (fishbone pattern in the second panel of **d**). They manifest in a modulation at the second harmonic of the driving field in the elongation of the electron distribution along the direction parallel to the electric field (right panel), as highlighted in the inset by its Fourier transform during the first half-cycles of the driving field (grey area). The scale of the vertical axis is fixed across the insets.

**Extended Data Fig. 10:**
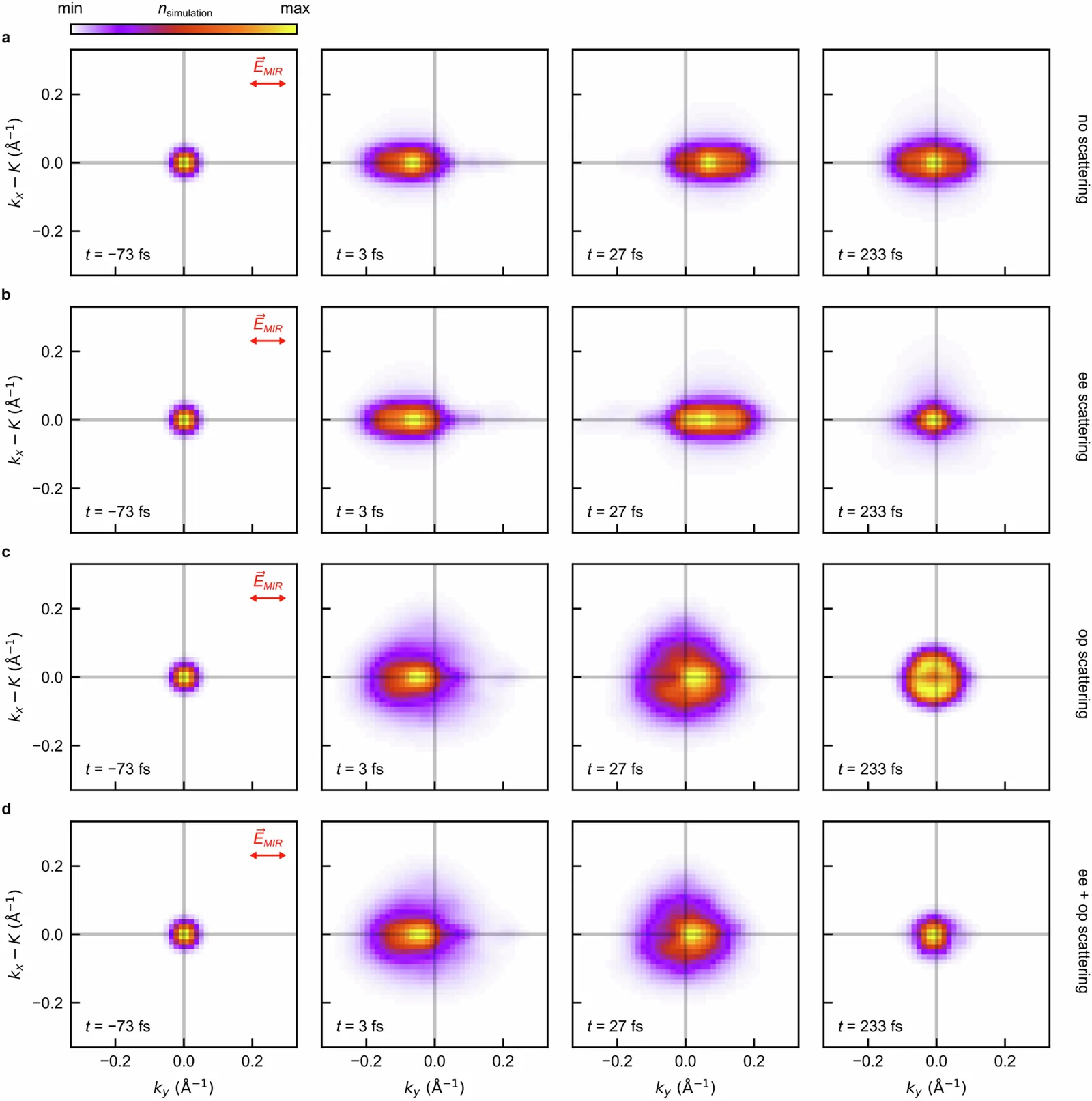
Scattering channels in momentum space. Simulated 2D carrier distribution in the conduction band for selected delay times with different scattering contributions. **a**, No scattering. **b**, Only electron–electron scattering. **c**, Only scattering with optical phonons. **d**, Combination of electron–electron scattering and scattering with optical phonons.

For an empty upper Dirac cone, we expect the carrier injection rate owing to LZM tunnelling to closely follow the magnitude of the electric field and the electron population to increase stepwise, with the highest injection rates occurring at the largest absolute field values. In an n-doped system and for high injection rates, as in the present experiment, Pauli blocking modifies this simple picture (Extended Data Fig. 5a). Electrons can only be injected when there is sufficient empty space in the conduction band near the K point. Consequently, intraband transport plays a decisive role in determining the injection timing. Carrier injection happens most efficiently during the intervals between the extrema of the electric field and the carrier displacement. Our simulations clearly show that this results in a phase delay between the population jumps and the field, which grows as more population has been transferred. Beyond the effects of Pauli blocking and scattering, the injection dynamics can be strongly influenced by quantum interference^59^. Here we find that they can cause a deviation of a step-like increase in the conduction band population (or electron distribution width) by transforming the plateau regions, following a strong injection phase, into local minima through destructive interference of electron wavepackets (Extended Data Fig. 9). Although the signal-to-noise ratio of the measured conduction band population does not allow us to resolve the jumps in the population during carrier injection experimentally, the dynamics of the increase of the carrier density can be well reproduced with the theory.

The simulations also show that the elongation of the conduction band electron distribution in momentum space is closely tied to the carrier population (Extended Data Fig. 5b). Notably, the weak oscillations in the width of the electron distribution, which are superimposed on the stepwise increase, become more pronounced with scattering (Fig. 4c). This effect is probably caused by a shortening of the effective injection time window. In the absence of scattering, the electron displacement follows the vector potential and is therefore phase-shifted by π/2 relative to the electric field. With scattering, the phase shift between the electric field and the displacement of the electrons shrinks, such that periods of rapid population increase occur only a few femtoseconds before an extremum in the displacement (Extended Data Fig. 5). Experimentally, the oscillation of the width of the electron distribution at twice the frequency of the driving field is clearly visible during the first three cycles of the electric field, that is, at times when there is still substantial carrier injection (Fig. 3c). The observed relative phase with respect to the electric field transient and the COM displacement agrees well with the simulations that include scattering. The data thus support the picture of a well that is periodically driven by the electric field and opened and closed by the oscillating carriers in the conduction band.

### Data processing

The new experimental platform presented here was developed to give direct access to strongly driven electron dynamics without relying heavily on theory or models. This philosophy also translates to data processing: our conclusions only rely on features present in the raw data. The previously described streaking compensation is the first and only step in the data analysis pipeline that is required to ensure that the observed dynamics can be attributed to processes that are happening in the solid and not the vacuum. Following that, the general strategy is that filtering is only used to remove technical noise and signals related to the matrix elements before the data are analysed or to enhance visibility in the figures. To ensure full transparency, the following contains a summary of all notable data processing steps for the presented figures.

The raw data from the delay line detector are binned with a spacing of 0.084 eV × 0.022 Å^−1^ × 0.022 Å^−1^ for the data in Fig. 1 and with a spacing of 0.084 eV × 0.011 Å^−1^ × 0.011 Å^−1^ for all other photoemission data.

The 3D data in Fig. 1c are symmetrized to remove the decrease in photoemission intensity caused by the angle of incidence of the XUV pulse.

The 2D momentum maps in Fig. 2a are obtained by integrating the 3D data within an energy window of 0.33 eV around distinct energy levels (0.25 eV, −0.25 eV, −0.75 eV, −1.25 eV, −1.75 eV). Before plotting, the data are further processed to enhance the visibility in the figure. First, a 1D simple moving average along both momentum axes (window size: 0.08 Å^−1^) is applied to the data four times. The filtered data are further processed by a 2D curvature filter described by equation (11) of ref. ^60^ with *C*_0_ = 0.2. Finally, a 2D median filter with window size 0.045 eV × 0.045 Å^−1^ is applied three times to clean up numerical noise.

The band structure cuts in Fig. 2b are obtained by integrating the 3D data within a momentum window of 0.08 Å^−1^ around *k*_*x*_ − *K* = 0. Before plotting, the data are further processed to enhance the visibility in the figure. First, a 1D simple moving average along the energy and momentum axes with window sizes of 0.59 eV and 0.08 Å^−1^, respectively, is applied four times. The filtered data are subject to a 2D curvature filter with *C*_0_ = 0.2. Finally, a 2D median filter with window size 0.34 eV × 0.045 Å^−1^ is applied three times. The raw data are shown in Extended Data Fig. 2b.

The applied field transient and current in Fig. 2c are extracted from the raw data as laid out in Methods sections ‘Electric field waveform reconstruction and streaking compensation’ and ‘Current extraction and Fourier analysis of the phase delay’. Before plotting, a 4-THz high-pass filter is applied to the data to remove slowly varying offsets. The data showing the increase of the carrier population in the conduction band were acquired under almost identical experimental conditions in which special care has been taken to align the MIR polarization such that the electric field exhibits no perpendicular component leading to coherent oscillations of the integrated photoemission intensity.

The 2D momentum maps in Fig. 3a are obtained by integrating the 3D data for energies higher than 170 meV. This energy level is chosen to minimize contributions of the valence band caused by the limited energy resolution of the setup. Before extracting the COM displacement and width, a Gaussian filter with a small window size 0.02 Å^−1^ × 0.02 Å^−1^ has been applied to the 2D data. We applied the same filter to the experimental 2D data for a better comparison between experiment and theory. For the theory data, this is necessary to reduce numerical or binning artefacts. Owing to its small window size, the filter does not change or remove any of the features retrieved for data analysis.

To extract the COM motion and change of the width of the carrier distribution shown in Fig. 3b,c, the plotted 2D maps are integrated along *k*_*x*_ and *k*_*y*_. For the data along *k*_*y*_, a Gaussian distribution with a constant offset (accounting for the background) is fitted to the integrated distribution. For the data along *k*_*x*_, the previously constant offset is modified with a linear decrease for growing *k*_*x*_ values to account for the decrease in background caused by the photoemission matrix element. The mean of the extracted fit for the *k*_*y*_ distribution is used to characterize the COM motion. Before plotting, a 15-THz high-pass filter is applied to these data to remove a slowly varying offset. The change of the standard deviation, Δσ, of the extracted fits is used to characterize the change of the width of the carrier distribution along *k*_*x*_ and *k*_*y*_.

The 2D momentum maps in Fig. 4a are obtained by integrating the simulated population of the conduction band and applying a Gaussian filter with a small window size of 0.02 Å^−1^ × 0.02 Å^−1^ to the 2D data, as described above for the data shown in Fig. 3a.

The extraction of the simulated current density and change of the width of the carrier distribution shown in Fig. 4b,c follows the procedures outlined for the experimental data.

## Online content

Any methods, additional references, Nature Portfolio reporting summaries, source data, extended data, supplementary information, acknowledgements, peer review information; details of author contributions and competing interests; and statements of data and code availability are available at https://doi.org/10.1038/s41586-026-11214-y.

## Supplementary information

Supplementary Video 1 Lightwave-driven dynamics in graphene. The video shows the subcycle dynamics of lightwave-driven graphene in the vicinity of the K point. On the left side, the electron population in the conduction band is shown, whereas the right panel shows a cut through the band structure at the K point. The field-induced asymmetry in the number of carriers with positive or negative momenta testifies to a macroscopic lightwave-driven current. Meanwhile, the emerging elliptical shape of the electron distribution in momentum space highlights the presence of LZM tunnelling into the conduction band. For late delay times, the electron distribution becomes circular again and relaxes back to its original shape by scattering.

Peer Review File

## Data Availability

All raw experimental data and computational data used in this study are publicly available from the Jülich DATA repository (https://doi.org/10.26165/JUELICH-DATA/HZFKTH)^61^.

## Code availability

Custom code used in this study is publicly available from the Jülich DATA repository (https://doi.org/10.26165/JUELICH-DATA/HZFKTH)^61^.
