# Supplementary material for: Subcycle videography of Landau–Zener–Majorana transitions in graphene
Source: Nature. 2026 Oct 28;659(8139):63–8. doi: 10.1038/s41586-026-11214-y (PMC13736222; doi:10.1038/s41586-026-11214-y)
Supplement: Supplementary file 1 [file 41586_2026_11214_MOESM2_ESM.pdf]

nature portfolio

Peer Review File

Subcycle videography of Landau-Zener-Majorana transitions in graphene

Corresponding Author: Professor Ulrich Höfer

Any redactions in this file are there to maintain patient confidentiality, the confidentiality of unpublished data, or to remove third-party material.

This file contains all reviewer reports in order by version, followed by all author rebuttals in order by version.

Version 1:

Reviewer comments:

Referee #1

(Remarks to the Author)
This manuscript demonstrates sub-cycle photoelectron momentum microscopy of lightwave-driven electrons in graphene with attosecond precision. The work reports intriguing laser-field–driven phenomena in which the electron distribution exhibits non-thermal characteristics attributed to Landau–Zener–Majorana transitions. Using their powerful experimental technique, the authors analyze the center-of-mass displacement and width dynamics of the electron distribution, which oscillate at frequencies of ω and 2ω relative to the driving laser field. Through full quantum-mechanical simulations, they further characterize coherent electronic dynamics and scattering processes in graphene under strong laser excitation. The reported observations represent a significant and timely advance in the field. While the results are compelling and important, several key aspects require clarification before the manuscript can be considered for publication.

- The authors claim that the observed response originates from a strong-field regime. Could the authors provide measurements (or videography data) showing the dependence on the laser-field magnitude? Previous studies have shown that photoexcitation in the weak-field regime is typically dominated by multiphoton transitions. Do the authors observe carrier distributions that can be attributed to such multiphoton processes?

- In Extended Data Fig. 6, the authors attempt to compare the weak-field and strong-field regimes. However, the weak-field case is simulated with ω=600 THz, whereas the strong-field case uses ω=30 THz, which does not constitute a fair comparison. To clearly distinguish the two regimes, the authors should use the same laser frequency (e.g., ω=30 THz) while varying only the field strength to illustrate the qualitative differences between weak-field and strong-field responses.

- In Extended Data Fig. 6b, the simulation shows a nodal-line feature along k_x−K=0. Such a nodal-line structure may arise from the peculiar pseudospin texture in graphene, which gives rise to a characteristic transition dipole-moment distribution in momentum space. In this case, the observed feature may not necessarily originate from Landau–Zener–Majorana transitions, but could instead result from conventional one- or multiphoton excitation processes. The authors should clarify whether the nodal-line structure can be uniquely attributed to Landau–Zener–Majorana transitions and discuss the possible contribution of these more trivial excitation mechanisms.

- In Fig. 2c, the authors present the profiles of the electric field and current density using red and blue symbols, respectively. According to the figure caption and the corresponding description in the main text, the current density is determined from the occupation imbalance in the photoemission map, while the electric field is extracted from momentum streaking (presumably of electrons in graphene). However, the observed occupation imbalance itself appears to arise from the momentum streaking process. It is therefore unclear how these two quantities are independently determined from the data. The authors should clarify how the current density and the electric field are distinguished in their analysis and how each quantity is extracted from the measurements.

Referee #2

(Remarks to the Author)
Eggers et al. present a compelling and technically sophisticated study on sub-cycle control and real-time observation of strong-field-induced electron dynamics near the K-points in graphene. The experimental achievement of combining ultrafast time-resolved momentum-resolved spectroscopy with intense MIR fields is remarkable, and the data quality is outstanding. The manuscript advances the field significantly by demonstrating a unique capability to probe non-equilibrium electron dynamics in momentum space with unprecedented resolution.
A central claim of the work is the first direct observation of Landau-Zener-Majorana (LZM) transitions in momentum space, which the authors argue are revealed through the distinct asymmetry that evolves due to LZM in the coherently displaced electron distribution and the characteristic 2w oscillation of the Gaussian width. The authors further disentangle coherent vs. incoherent processes and adiabatic vs. non-adiabatic dynamics, offering a rich framework for interpreting the results. The chosen system, graphene on a SiC substrate, provides an ideal platform for such studies due to its well-defined Dirac cone and strong light-matter coupling.

However, while the data are extraordinary and the interpretation is plausible, I remain unconvinced that the evidence presented is sufficient to unambiguously establish the observation of LZM transitions. The key concern is that conventional photoexcitation via MIR photon absorption at 124 meV (30 THz) could, in principle, reproduce the observed asymmetries and dynamics, especially given that absorption is energetically allowed in the very same time window where LZM transitions are claimed to appear.
The argument that the observed asymmetry arises from LZM transitions rather than perturbative photoexcitation relies heavily on the expected anisotropy in the excitation matrix elements. However, this argument is not sufficiently supported by a direct comparison with the expected photoexcitation pattern under the actual experimental conditions. In particular, this most important argumentation is moved to an extended figure. Furthermore, Extended Figure 6, which is the primary evidence for the distinction between LZM and absorption, shows simulations for 600 THz (5 µm, 2.5 eV), which is completely irrelevant to the experimental setup (30 THz, 10 µm, 124 meV). This raises a critical question: What does the photoexcitation pattern look like at 30 THz, under realistic conditions of the given energy resolution, momentum resolution, and signal-to-noise ratio? Is such a pattern resolvable? Can it be distinguished from the predicted LZM signature under the same experimental constraints?
Furthermore, the authors invoke the 2w oscillation of the Gaussian width as support for LZM dynamics. However, this feature could equally arise from photoexcitation-induced motion of the electron occupation disc in momentum space. When the disc is sufficiently displaced, Pauli blocking is lifted, allowing excitation to occur. This process would repeat twice per optical cycle, showing the 2w modulation. Thus, unless the authors can rigorously rule out this alternative explanation, the observed oscillation cannot be taken as definitive evidence for LZM transitions in momentum space, which is the primary claim of the manuscript.
In summary, while the authors are likely correct in attributing the dynamics to LZM processes, at least in part, and the data are fully consistent with this interpretation, the current manuscript does not provide conclusive proof that LZM transitions are unambiguously observed. The claim of a "first direct observation" in momentum space requires stronger, more rigorous evidence.

Additional concerns requiring clarification and improvement:
1. Dark corridor and non-equilibrium effects: The argument that the dark corridor is irrelevant around the Dirac point is questionable here. With electrons excited up to ~1 eV, the dark corridor may significantly influence the measured and analyzed asymmetry of the distribution in the conduction band. This must be explicitly addressed in the theoretical modeling and analysis.
2. Figure 1b: Should the light blue area in the VB be the signature of holes and is this correct as plotted? Wouldn't it be better to plot this for the doped graphene system, as is used in the experiment?
3. Unexplained features in Fig. 2b (middle): The presence of unexplained "holes" in the valence band, particularly visible in the positive momentum direction, raises concerns about the reliability of the Gaussian fitting procedure used in the conduction band. Such artifacts could falsify the extracted momentum distributions and must be investigated and explained.
4. Figure 2: The phrase “a similar measurement” is vague and should be clarified.
5. Band alignment in Fig. 2: The experimental band dispersions do not align well with theoretical predictions, especially between panels 2b (middle) and 2b (right). As an idea: this discrepancy might be corrected if substrate-induced band modifications are considered. The authors might try to incorporate known effects from graphene on SiC (e.g., as reported by Zhou et al., Nature Materials 6, 770 (2007)) into their analysis.
6. Figure 3a: It is unclear whether the curvature filtering was applied to the raw data or to a pre-processed dataset. The analysis procedures, throughout the manuscript, must be explicitly described, including details on data integration in energy and momentum space, background subtraction, and the treatment of curvature.
7. Visualization and interpretation: Color bars could be added to all relevant figures to clearly indicate intensity scales and the spectra where curvature filtering was applied. This would greatly improve interpretability and transparency.
8. Analysis methodology: The authors must provide a detailed description of how momentum widths and other fitting parameters were extracted: e.g., whether from raw data, integrated regions, or filtered data. The integration areas, fitting windows, and error estimation procedures should be clearly documented.
9. Data availability: The raw experimental data underlying the presented results have undergone extensive processing, including corrections of momentum and energy shifts, background subtraction, curvature filtering, integration over different momentum and energy windows, and Gaussian fitting. As currently presented, the analysis pipeline is not transparent, and the lack of access to the original, unprocessed data and the used analysis scripts severely limits the ability of readers to verify, interpret, or reproduce the key findings. Given the extraordinary claims made in the manuscript, particularly the first direct observation of LZM transitions in momentum space, full reproducibility is essential.
In my opinion, the authors should make the raw data, along with detailed documentation of all analysis steps, including integration regions, filtering procedures, correction algorithms, fitting algorithms, and parameter choices, publicly available.

This would greatly enhance the scientific rigor and transparency of the work, aligning with best practices for data sharing in modern physics research.

As a final note, I would like to express growing concern about our increasing tendency in scientific manuscripts to prioritize narrative flair. Phrases such as “notoriously elusive” (is this really the case for LZM transitions?), “interband quantum leaps” (instead of “interband transitions”), “videography” (used in place of “electronic structure dynamics” in the title), or the suggestion that momentum distributions “conspire” to produce a certain pattern risk undermining the rigor and objectivity expected in high-impact research. While vivid language can enhance communication, its overuse can detract from scientific clarity and credibility.
It is the authors’ decision how to phrase the manuscript, but they should be aware that such stylistic choices may increasingly annoy readers, especially when they appear to prioritize rhetorical effect over precision.

Conclusion:
The experimental results are groundbreaking and represent a major step forward in ultrafast momentum-resolved spectroscopy. The potential for observing LZM transitions in momentum space is highly significant and worthy of publication in Nature. However, the current manuscript falls short of providing the rigorous, unambiguous evidence required to support such a bold claim.
I strongly encourage the authors to address the above concerns, particularly by simulating and comparing the expected photoexcitation patterns at 30 THz under realistic experimental conditions, and by rigorously ruling out alternative explanations. If these points are satisfactorily resolved, I would be fully supportive of a resubmission to Nature.

Referee #3

(Remarks to the Author)
The work by V. Eggers et al. demonstrates sub-cycle mapping of k-resolved electron population dynamics under strong fields in graphene using time-resolved ARPES, with unprecedented access to the full Brillouin zone. In particular, the paper focuses on visualizing light-driven carrier acceleration and non-adiabatic Landau-Zener-Majorana transitions with sub-cycle resolution.

The paper shows direct observation of light-driven acceleration of carriers along the polarization direction of the field (ky), reversing with each half-cycle, from which the intraband current is extracted. This current is shown to be phase shifted relative to the driving field by less than pi/2, which is the value expected from purely coherent acceleration of carriers by the field. In addition, it is shown that the shape of the k-resolved light-driven electron distribution is elliptical, with the widths along the kx and ky directions changing periodically, and gradually evolves to an almost circular shape approaching (but not reaching) the equilibrium value for 233fs.

These experimental observations are reproduced and explained by solving the semiconductor Bloch equations of a two-band tight binding graphene model that includes a phenomenological dephasing T2, and approximate electron-electron scattering and electron-phonon scattering rates. These calculations identify that (i) interband coupling is responsible for the elongation of the electronic distribution along the field polarization direction, (ii) electron-electron and electron-phonon coupling are responsible for the thermalization of the distribution in a circular shape, and the latter is responsible for the deviation of the current phase shift from that of fully coherent dynamics.

The work represents a clear experimental advance by extending sub-cycle ARPES to access the full Brillouin zone and provides a compelling visualization and picture of strong-field dynamics in graphene. The results are clearly presented and both experimental and theoretical methodologies are of high quality and convincing (e.g., appropriate compensation of streaking in the continuum, obtention of e-e and e-ph scattering rates).

One issue is that despite the main experimental advance being the full BZ access (+- 2.14 Angstroms^-1), all analyzed dynamics are confined near the K point (|k - K| < 0.16 Angstroms^-1). The physics explored (Dirac cone + LZM tunnelling) is inherently two-band (as the theoretical approach demonstrates) and already accessible with the momentum range of previous experiments (e.g., S. Ito et al. Nature 616 (2023)). It seems that the extended momentum range does not play an essential role in the present study, and its potential to explore multi-band physics away from K (e.g., near Gamma) is unused. Before reaching a decision, I would like the authors to address the following comments.

1. It is unclear why a single Fourier-averaged phase shift of the current is extracted. Since this phase shift is attributed to scattering, it should depend on time, carrier density and k-space distribution. Therefore, this should not lead to a constant phase shift, but rather to a chirp. Indeed, in Figure 2c one can observe that the phase shift is reduced in the trailing edge compared to the peak of the field. Do the simulations reproduce this time-dependent phase shift? Perhaps a local time-frequency analysis would provide a more meaningful characterization of the dynamics.

2. In the theory, relaxation processes are effectively described as driving the system towards the field-free equilibrium distribution. While this is a common approximation, it is a still open question whether under such strong-field conditions and fast timescales the system indeed relaxes towards the initial equilibrium state, rather than towards a transient hot-carrier distribution. Do the authors have access to the experimental population distributions at longer times (> 250 fs)? If so, are they different than the initial, field-free equilibrium distributions? And, if so, do the dynamics predicted by theory change in some way by using these final "transient" electron distributions as the target for relaxation rather than the initial field-free

equilibrium populations? More generally, given the limited energy resolution, it is not entirely clear whether the observed isotropic contraction should be interpreted as true thermalization to a Fermi-Dirac distribution, rather than as a relaxation towards a more isotropic but still non-equilibrium transient state.

3. While k-resolved scattering is implicit in the theory, it is not clear how it plays a role in the dynamics, especially when the work restricts dynamics to a narrow region around the K point and interprets the results with a single effective scattering time. Given the momentum-resolved nature of the experiment, it would be very valuable to clarify to what extent momentum-dependent scattering plays a role in the observables; the technique presented here seems ideally suited to probe such k-dependent scattering mechanisms. Could the authors comment on whether this could be accessed experimentally, for example by varying the driving field amplitude or frequency (to explore different k-space regions), or by performing measurement at lower field strengths to probe near-equilibrium scattering? This would give a more direct characterization of strong-field-driven scattering processes.

4. The extracted current is based on population asymmetry and therefore corresponds to the intraband contribution. I assume that the theory neglects the interband current contribution (not the coupling), but it would be useful to clarify if the current in the theory is extracted in the same way as in the experiment.

5. The polarization of the driving field is chosen along ky, where the two branches of the Dirac cone have the same photoemission intensity. I assume this choice is made in order to minimize the effect of different matrix elements in the measured ARPES signal. Is this correct? Assuming the photoemission matrix elements are well characterized, are there any limitations apart from this one to explore the dependence of the dynamics on the driving field polarization?

Version 2:

Reviewer comments:

Referee #1

(Remarks to the Author)
The authors have substantially addressed my concerns. In particular, the revised manuscript now includes simulations at the experimental frequency of 30 THz for the weaker field, making the comparison more consistent. Although the term "directly observe LZM transitions" could still be debated under a strict standard, I recognize that such a standard may be too restrictive in this context. In summary, I consider that my main concerns have been resolved, and I have no further major concerns regarding the manuscript.

Referee #2

(Remarks to the Author)
I have examined the manuscript together with the referee’s reports and the authors’ comprehensive replies. The revised version is markedly clearer: the distinctions between perturbative absorption and genuine strong-field processes are now explicitly emphasized, eliminating any ambiguity for readers. Consequently, the discussion offers more guidance to the photoemission community (one of the paper’s principal audiences) usually working in the perturbative regime, so that concerns about linear or multiphoton absorption influencing the results are dispelled. The addition of the Keldysh parameter for graphene and the more detailed description of data processing further strengthen the work. On this basis, I fully recommend publication in Nature.

Referee #3

(Remarks to the Author)
The authors have substantially improved the clarity of the manuscript and addressed my questions.

1. My concern was about the actual use of the novel experimental capabilities in the presented work. The authors have clarified that , while the present study does not make use of the ability to track dynamics across the full Brillouin zone, it does rely on both the access to the K points at the BZ edge and 2D momentum mapping with sub-cycle temporal resolution.

Previous works have demonstrated XUV tr-ARPES of carrier dynamics at the graphene K point (I. Gierz et al., Phys. Rev. Lett. 115 086803 (2015)) and 2D momentum mapping covering the full BZ (e.g., S. Dong et al., Nature Comm. 14 5057 (2023)). Yet, the present work advances these techniques by reaching sub-cycle resolution with the use of mid-IR pulses. This sub-cycle resolution is indeed central to support the observation of LZ transitions, and is thus a key advance.

2. I thank the authors for performing the time-dependent phase analysis.

3. I thank the authors for the clarification.

4. I thank the authors for the clarification. Just a small note that I'm certain the authors are aware of but might help the readers not getting confused: strictly speaking, the measured population asymmetry is not itself and intraband current, since the current also requires to multiply the populations by the band velocity. Perhaps there are some instances in the paper where intraband can be changed or clarified. I am happy with the term “instantaneous asymmetric electron distribution in momentum space […] associated with […] a

microscopic intraband current” that the authors use.

5. I thank the authors for the explanation.

Regarding the other Referee's remarks, I found that they were properly addressed, although I found the answer regarding multi-photon/tunnelling dynamics somewhat confusing. The authors use the "Keldysh" parameter used by Higuchi et al., which is reasonable, but this parameter is momentum-dependent; it is not a universal parameter, rather it shows that different parts of the BZ of graphene will have different "Keldysh" parameters. The authors choose precisely the momentum point with gap resonant to the one-photon transition to obtain a parameter much less than one, and use it to justify the tunnelling picture. First, it is somewhat misleading to choose a one-photon resonant region to justify that excitation occurs through tunnelling and second, even if that is the case, that only implies that the "Keldysh" parameter is less than one for that particular momentum. For larger momenta, the "Keldysh" parameter might be comparable to 1. A stronger argument would be therefore to show that all momenta relevant for the LZ dynamics show a "Keldysh" parameter much smaller than one. That said, I don't think this is a fundamental issue, since I believe the physics observed by the authors do show evidence of LZ transitions: the CB population increase and the elongated shape of the population distribution. I do not believe the authors should try to justify that dynamics are governed solely by tunnelling. In that sense, the statement of Extended Data Figure 7: "[...] marks the absence of perturbative single- or multi-photon absorption" is too strong, but also not needed. Residual photonic contributions would not invalidate the central point.

In light of the above, I would be in favour of publication in Nature.

Open Access This Peer Review File is licensed under a Creative Commons Attribution 4.0 International License, which permits use, sharing, adaptation, distribution and reproduction in any medium or format, as long as you give appropriate credit to the original author(s) and the source, provide a link to the Creative Commons license, and indicate if changes were made.
In cases where reviewers are anonymous, credit should be given to 'Anonymous Referee' and the source.
The images or other third party material in this Peer Review File are included in the article’s Creative Commons license, unless indicated otherwise in a credit line to the material. If material is not included in the article’s Creative Commons license and your intended use is not permitted by statutory regulation or exceeds the permitted use, you will need to obtain permission directly from the copyright holder.
To view a copy of this license, visit https://creativecommons.org/licenses/by/4.0/

Replies to Referee #1

Referee #1: *This manuscript demonstrates sub-cycle photoelectron momentum microscopy of lightwave-driven electrons in graphene with attosecond precision. The work reports intriguing laser-field–driven phenomena in which the electron distribution exhibits non-thermal characteristics attributed to Landau–Zener–Majorana transitions. Using their powerful experimental technique, the authors analyze the center-of-mass displacement and width dynamics of the electron distribution, which oscillate at frequencies of ω and 2ω relative to the driving laser field. Through full quantum-mechanical simulations, they further characterize coherent electronic dynamics and scattering processes in graphene under strong laser excitation. The reported observations represent a significant and timely advance in the field. While the results are compelling and important, several key aspects require clarification before the manuscript can be considered for publication.*

Reply 1.1: We thank the Reviewer for taking the time to carefully review our manuscript, for the insightful comments, the helpful suggestions, and the recognition of the importance of our work.

Referee #1: *- The authors claim that the observed response originates from a strong-field regime. Could the authors provide measurements (or videography data) showing the dependence on the laser-field magnitude? Previous studies have shown that photoexcitation in the weak-field regime is typically dominated by multiphoton transitions. Do the authors observe carrier distributions that can be attributed to such multiphoton processes?*

Reply 1.2: Indeed, the observed response can only originate from strong-field light-matter interaction because the electron distribution significantly changes on time scales shorter than a cycle of the driving field (see e.g. subcycle intraband currents in Fig. 2 and subcycle inter- and intraband dynamics in Fig. 3). Perturbation theory, in contrast, works only as long as single- or multi-photon transition probabilities per oscillation cycle are very low. Perturbative nonlinearities can, thus, unfold only on multi-cycle time scales. This is not what we observe in the experiment.

The Keldysh parameter γ, which is commonly used to distinguish between the weak-field and strong-field regimes of light-matter interaction, confirms this picture. In case of graphene, γ can be denoted as $\gamma = \frac{\omega\Delta}{2v_F|e|E_0}$ [Higuchi *et al.*, Nature **550**, 224 (2017)], where Δ is the transition energy, ω the light frequency and E_0 the electric field

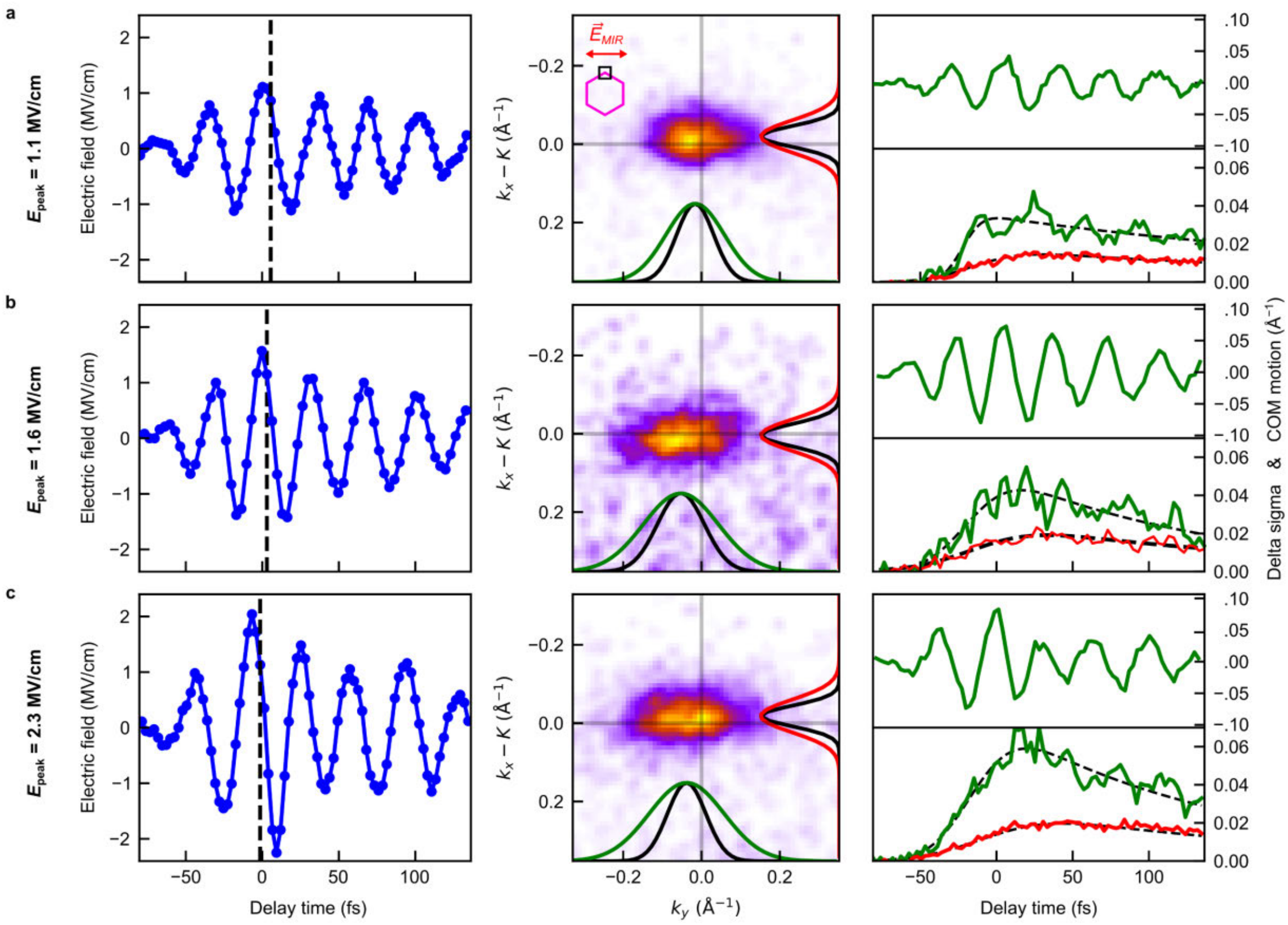

Figure R1 | Field-dependent electron dynamics in 2D momentum space.

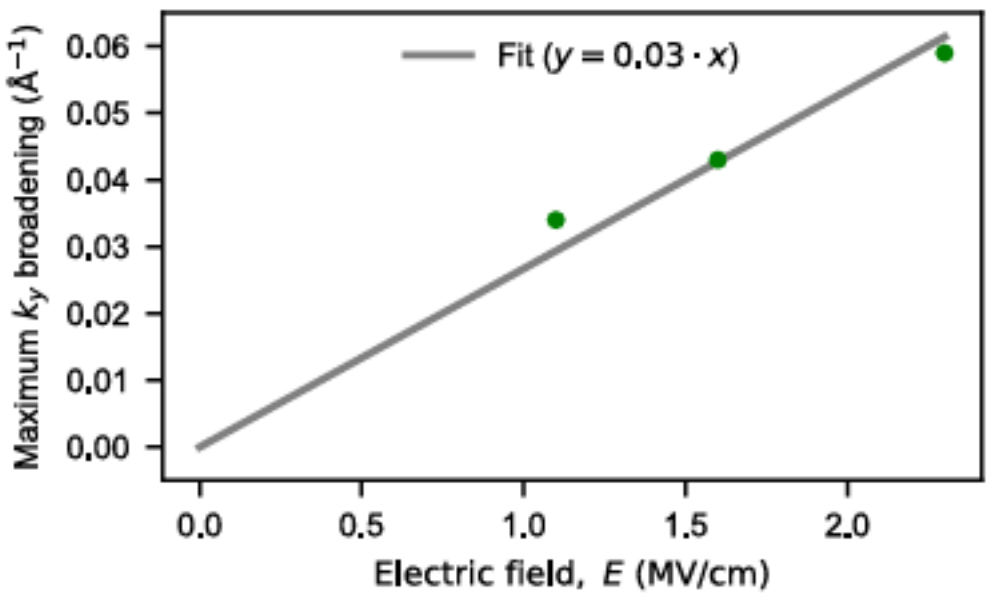

Figure R2. Field-dependent broadening along k_y.

amplitude. If we identify Δ with the MIR photon energy of 124 meV, γ becomes 0.075. This clearly indicates that our measurement explores the strong-field regime which is usually already reached at $\gamma \leq 1$. If one could switch off intraband acceleration, the relevant transition energy from the valence band to the unperturbed conduction band would increase to two times the doping energy of 200 meV, i.e. to Δ = 400 meV. Even then, $\gamma = 0.24$ would comfortably place our measurements in the strong-field regime.

Given these numbers, the MIR intensity would have to be reduced by 2-3 orders of magnitude to reach the perturbative regime. The anticipated dynamical changes of the electron distribution then become so tiny that they would require a high signal-to-noise ratio for subcycle detection that cannot readily be achieved with the maximum count rate of roughly 20 kHz (integrated total over all electron energies and momenta) of our time-of-flight momentum microscope. Realistically, the experimental dependence of subcycle photoemission on the laser-field magnitude can thus only provide a consistency check for the expected strong field behaviour.

We have performed such measurements at three distinct field strengths in the vicinity of the value reported in the manuscript, namely at 1.1 MV/cm (1.6 GW/cm^2), 1.6 MV/cm (3.4 GW/cm^2) and 2.3 MV/cm (7.0 GW/cm^2) (Fig. R1). They show how the broadening of the electron distribution parallel to the field increases linearly with the field amplitude (Fig. R2), while the broadening along the direction perpendicular to the field remains largely unchanged. This underscores that we are in the strong-field regime, since the maximum broadening that can be achieved through LZM transitions is connected to the maximum displacement of the electron distribution (because we always feed in more electrons mainly at the K point) and the displacement scales linearly with the field in the field-driven regime according to Bloch's acceleration theorem. Absorption-driven processes should scale with the intensity, for single-photon absorption, and powers of the intensity, for multi-photon absorption. The n-doping in our experiment placing the Fermi level 200 meV above the Dirac point prevents single- and two-photon absorption by Pauli blocking; the first available multi-photon transition would involve 2 × 200 meV / 124 meV > 3 photons. Related scaling laws cannot be observed in the data (Fig. R2).

It would of course be interesting to bridge the transition from perturbative regime to the strong-field regime and perform corresponding band-structure videography measurements as suggested by the Referee. In our opinion, a promising approach in that direction would be to perform sub-cycle measurements at photon energies approaching the visible range while keeping the field strength high. Unfortunately, such measurements are currently not possible with our experimental setup.

We now clearly state in the manuscript that the observed extreme carrier redistribution on subcycle timescales is a hallmark of non-perturbative (strong-field) dynamics (change #3). We added a new Methods section, which follows the above discussion to derive the Keldysh parameter in graphene (change #13), and made additions to the manuscript to highlight the role of the Keldysh parameter in the experiment (change #25). We also added a discussion connecting the shape of the observed carrier distribution to the original formula for diabatic LZM transitions (change #8).

Referee #1: *- In Extended Data Fig. 6, the authors attempt to compare the weak-field and strong-field regimes. However, the weak-field case is simulated with ω=600 THz, whereas the strong-field case uses ω=30 THz, which does not constitute a fair comparison. To clearly distinguish the two regimes, the authors should use the same laser frequency (e.g., ω=30 THz) while varying only the field strength to illustrate the qualitative differences between weak-field and strong-field responses.*

Reply 1.3: In the case of the linearly dispersing Dirac cone of graphene, the transition from the weak-field to the strong-field regime can equivalently be explored by varying the electric field strength or the frequency of the excitation because $\gamma \sim \omega / E_0$. We opted for higher frequencies when simulating the weak-field regime for better comparability with existing experiments. The simulation reproduces the typical hot carrier distributions observed with previous ARPES studies. In particular, the pseudospin texture of graphene results in transitions featuring finite momenta perpendicular to the polarization of the incoming light. This distribution contrasts the field-induced acceleration and tunnelling in the direction parallel to the field, characteristic of the strong-field regime.

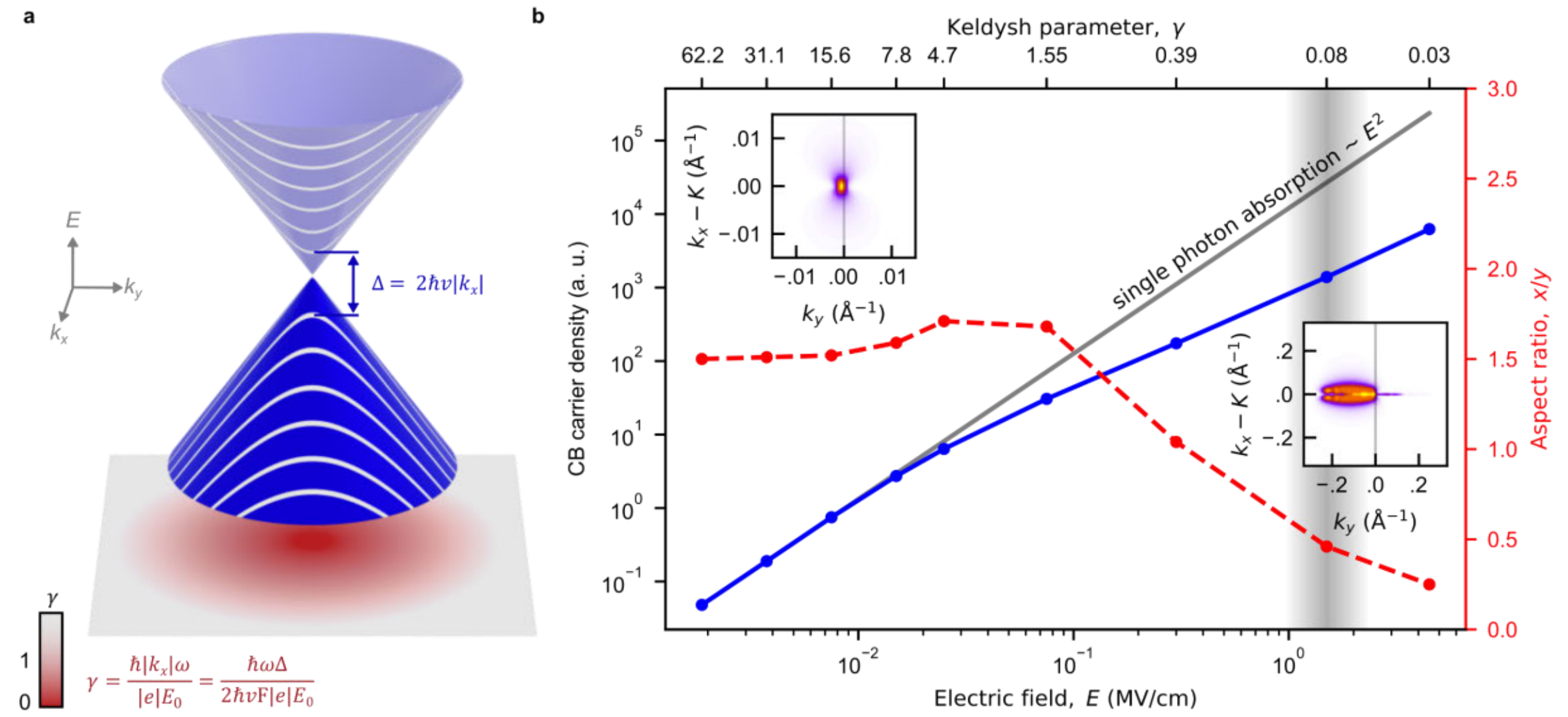

Figure R3. Population-transfer scaling in the weak- and strong-field regime. **a,** Illustration of the varying Keldysh parameter in graphene. In **b**, the Keldysh parameter is evaluated for a gap corresponding to single-photon absorption. **b,** Simulated 2D change of the population in the conduction band (blue curve) and aspect ratio of the carrier distribution (red curve) after interacting with the field for different field strengths in an undoped sample without scattering.

We realize that this comparison is misleading if not properly motivated. Following the Reviewer's advice we therefore simulated the dynamics again, varying the field strength at a constant frequency of 30 THz. Assuming an ideal, undoped Dirac cone, the number of excited carriers in the conduction band and the position and aspect ratio of the carrier distribution in momentum space immediately after interaction with the light pulse reveal unambiguous signatures of the different interaction regimes (Fig. R3). For Keldysh parameters $\gamma \gg 1$, i.e. in the perturbative regime, the number of carriers scales quadratically with the field, indicating single-photon absorption. Meanwhile, in the strong-field regime ($\gamma \ll 1$), the number of excited carriers is trending towards saturation, indicative of light-field-induced tunnelling as the transition mechanism [Schultze *et al.*, Science **346**, 1348 (2014)].

The most striking distinction between the two regimes lies in the deflection of the centre of mass and the aspect ratio of the carrier distribution. In the weak-field regime (Fig. R3, left inset) it is always centred about the Dirac point and broader along the momentum direction perpendicular to the light field. It inverts in the strong-field regime, resulting in the strongly elliptical distribution observed in the experiment with large elongation and excursion in the direction parallel to the applied field (Fig. R3, right inset). The basic physics for this transition is easy to understand. In the perturbative regime, the electric field is far too weak to cause a noticeable displacement of the electrons on the subcycle time scale. The distribution is solely determined by the matrix elements for photoexcitation. For sufficient field strengths, however, the electrons can be accelerated substantially over one half cycle. The momentum distribution is then governed by the interplay of intraband acceleration along the direction of the electric field and the carrier transfer from the valence band to the conduction band in the vicinity of the Dirac point. In perfect agreement with the experiment, it becomes more elliptical with increasing field amplitude.

The grey shaded area in the plot marks the experimentally explored field strength range which is comfortably placed in the strong-field regime. The Keldysh parameter γ, the deflection and the aspect ratio *x/y* make it obvious that our experiment explores strong-field phenomena more than an order of magnitude beyond the transition to the perturbative regime.

We have modified Extended Data Fig. 6 to show the evolution of the carrier distribution at 30 THz and lower fields (change #23), and included Fig. R3 as Extended Data Figure 7 in the manuscript (change #24). We also complemented the main text to clarify this point (change #7).

Referee #1: *- In Extended Data Fig. 6b, the simulation shows a nodal-line feature along k_x−K=0. Such a nodal-line structure may arise from the peculiar pseudospin texture in graphene, which gives rise to a characteristic transition dipole-moment distribution in momentum space. In this case, the observed feature may not necessarily originate from Landau–Zener–Majorana transitions, but could instead result from conventional one- or multiphoton excitation processes. The authors should clarify whether the nodal-line structure can be uniquely*

attributed to Landau–Zener–Majorana transitions and discuss the possible contribution of these more trivial excitation mechanisms.

Reply 1.4: We agree with the Reviewer that the pseudo spin of graphene is expected to lead to a nodal-line feature in the k_y direction in perturbative absorption, as well as in non-perturbative LZM transition. Therefore, we do not attribute this feature uniquely to LZM transitions. In addition, the nodal line is very hard to resolve experimentally with our energy resolution. On the other hand, the main experimental evidence for LZM transitions lies in the observed elongated shape and the COM motion of the CB momentum distribution, which are both clearly observable.

However, in the strong-field regime, there are even more effects that can affect the structure of the electron distribution. Experimentally, we also observe an additional intriguing feature in the 2ω oscillation in the k_y width of the electron distribution, which we try to understand in Extended Data Fig. 9. Our simulations reveal that features along the k_y directions near $k_x - K = 0$ sensitively depend on the choice of the dephasing time T_2. The electron distribution lacks any fine structure for very fast dephasing times ($T_2 = 0.1$ fs and $T_2 = 1$ fs) when any phase relation between wave packets subsequently injected is lost. Here, the evolution of the width of the distribution can best be described by a step-like increase. For longer T_2 values, where quantum interference effects from repeated LZM transitions are possible, a time-dependent fine-structure emerges in the electron distribution, which leads to pronounced 2ω oscillations. This points to Landau-Zener-*Stückelberg*-Majorana interferometry as the origin of this feature. Since we do not directly resolve the fine structure experimentally, we did not make a major claim about it in the manuscript.

In the revised manuscript, we now highlight the absence of perturbative one- or multi-photon absorption in the observed low-frequency strong-field regime (change #5) and we clarify our discussion of the LZ*S*M features (change #9).

Referee #1*:* *- In Fig. 2c, the authors present the profiles of the electric field and current density using red and blue symbols, respectively. According to the figure caption and the corresponding description in the main text, the current density is determined from the occupation imbalance in the photoemission map, while the electric field is extracted from momentum streaking (presumably of electrons in graphene). However, the observed occupation imbalance itself appears to arise from the momentum streaking process. It is therefore unclear how these two quantities are independently determined from the data. The authors should clarify how the current density and the electric field are distinguished in their analysis and how each quantity is extracted from the measurements.*

Reply 1.5: We thank the Reviewer for spotting this lack of clarity. In fact, the driving electric field and the microscopic current density can be determined from the measured photoemission data independently [Reimann *et al.*, Nature **562**, 396-400 (2018)]. To extract the electric field, we first evaluate the momentum shift, $\Delta k_{||}(t)$, of the *entire* photoemission signal (including the occupied valence bands), for every delay time *t*. This shift originates from the acceleration of free electrons in vacuum by the parallel component $\boldsymbol{E}_{||}$ in front of the sample. The field is then simply given by

$$\Delta \boldsymbol{p}_{||}(t) = -e \int_t^{\infty} \boldsymbol{E}_{||}\left(t'\right)\, dt' \;\rightarrow\; \boldsymbol{E}_{||}(t) = \frac{\hbar}{e}\frac{\mathrm{d}}{\mathrm{d}t}\Delta \boldsymbol{k}_{||}(t).$$

The momentum shift is accompanied by a well-defined energy shift, Δε, which can also be calculated by using the free-electron dispersion:

$$\Delta\varepsilon\left(k_{||}\right) = \varepsilon(\boldsymbol{k} + \Delta\boldsymbol{k}) - \varepsilon(\boldsymbol{k}) = \frac{\hbar^2}{m} k_{||}\Delta k_{||} + \frac{\hbar^2}{2m}\Delta k_{||}^2$$

The measured photoemission spectra are finally shifted along the momentum and energy directions to compensate for the streaking-induced rigid movements of the entire band structure.

After this streaking compensation, the remaining dynamics unfold *within* the band structure. Consequently, we can independently determine the microscopic current density, $j(t)$, by comparing photoemission intensities above the Dirac point with negative and positive momenta for every delay time.

We have modified the Methods section to elucidate this point (change #12). In the main text, we clarify that not only the data shown in Fig. 2b but also those in Fig. 3a are already compensated for streaking effects (change #6).

Replies to Referee #2

Referee #2: *Eggers et al. present a compelling and technically sophisticated study on sub-cycle control and real-time observation of strong-field-induced electron dynamics near the K-points in graphene. The experimental achievement of combining ultrafast time-resolved momentum-resolved spectroscopy with intense MIR fields is remarkable, and the data quality is outstanding. The manuscript advances the field significantly by demonstrating a unique capability to probe non-equilibrium electron dynamics in momentum space with unprecedented resolution.*

A central claim of the work is the first direct observation of Landau-Zener-Majorana (LZM) transitions in momentum space, which the authors argue are revealed through the distinct asymmetry that evolves due to LZM in the coherently displaced electron distribution and the characteristic 2w oscillation of the Gaussian width. The authors further disentangle coherent vs. incoherent processes and adiabatic vs. non-adiabatic dynamics, offering a rich framework for interpreting the results. The chosen system, graphene on a SiC substrate, provides an ideal platform for such studies due to its well-defined Dirac cone and strong light-matter coupling.

Reply 2.1: We thank the Reviewer for taking the time to carefully review our manuscript, for the insightful comments, the helpful suggestions, and the high praises of our work.

Referee #2: *However, while the data are extraordinary and the interpretation is plausible, I remain unconvinced that the evidence presented is sufficient to unambiguously establish the observation of LZM transitions. The key concern is that conventional photoexcitation via MIR photon absorption at 124 meV (30 THz) could, in principle, reproduce the observed asymmetries and dynamics, especially given that absorption is energetically allowed in the very same time window where LZM transitions are claimed to appear.*

The argument that the observed asymmetry arises from LZM transitions rather than perturbative photoexcitation relies heavily on the expected anisotropy in the excitation matrix elements. However, this argument is not sufficiently supported by a direct comparison with the expected photoexcitation pattern under the actual experimental conditions. In particular, this most important argumentation is moved to an extended figure. Furthermore, Extended Figure 6, which is the primary evidence for the distinction between LZM and absorption, shows simulations for 600 THz (5 µm, 2.5 eV), which is completely irrelevant to the experimental setup (30 THz, 10 µm, 124 meV). This raises a critical question: What does the photoexcitation pattern look like at 30 THz, under realistic conditions of the given energy resolution, momentum resolution, and signal-to-noise ratio? Is such a pattern resolvable? Can it be distinguished from the predicted LZM signature under the same experimental constraints?

Reply 2.2: We thank the Reviewer for their encouraging words about our experimental achievement and the positive assessment of the importance of the results. We hope that we can in the following dispel their concerns about a possible influence of perturbative photoexcitation. The excitation pattern in momentum space is one argument why we exclude a significant contribution of perturbative photoabsorption. The other one is that one cannot expect perturbative photoexcitation to be competitive with electron tunnelling at the low frequencies and high field strengths of our excitation pulses. Our data directly show in momentum space that the driving field

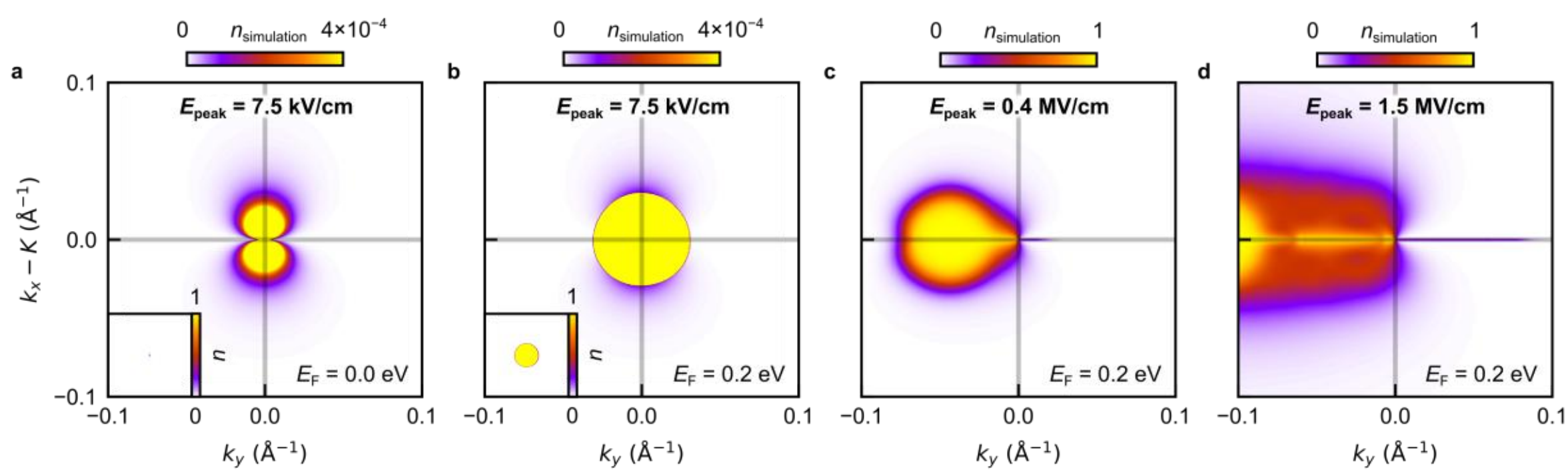

Figure R4. Theoretical conduction band (CB) momentum patterns for a driving field of 30 THz and different strengths of the electric field. In **a**, the Fermi level is adjusted to prevent Pauli blocking. In **a** and **b** the temperature is set to 3 K and the colormap limits are exaggerated to reveal the tiny population increase.

massively displaces the whole conduction band distribution back and forth on subcylce time scales. In this way, they reveal a hallmark of strong-field dynamics in solids.

In Fig. R4 we compare calculated excitation patterns for four different cases, all driven at 30 THz. In the perturbative regime (E_0 = 7.5 kV/cm), the CB population increase occurs mainly in the k_x direction, i.e. perpendicular to the direction of the electric field (Fig. R4a). This is a consequence of optical selection rules due to the pseudo spin texture of graphene. In the strong-field regime, the population increase occurs in a narrow range of k_x values close to the Dirac point with low tunnelling barriers (Fig. R4d). It gets elongated in the k_y direction due to acceleration by the carrier field.

While it appears possible to resolve these different patterns experimentally for a (hypothetical) undoped sample, the situation is different for the actual doped sample. There, for low enough electric fields, perturbative absorption processes can lead to a population increase only for values of k_x-K = 0.04 Å^{-1}. They also result in an increased population in the k_x direction (Fig. R4b). However, the population difference is very small (10^{-4}) and thus difficult to detect experimentally. This can be understood by considering that conventional absorption, according to Fermi's Golden Rule, relies on small perturbations that build up a population over many oscillation cycles. Moreover, the expected asymmetry would be completely washed out by the widening of the Fermi circle at 300 K. At higher fields, we can consider the case where the applied electric field is strong enough to shift the conduction band population away from the Dirac point and lift Pauli blocking. At 30 THz, such a shift requires a minimum field strength of 0.4 MV/cm (Fig. R4c). Under this condition, the calculated pattern clearly does not show a signature of perturbative excitation anymore (Fig R4c). Rather the pattern closely resembles the one obtained for the field strength of the experiment (1.5 MV/cm, Fig. R4d). As for the reliability of this theoretical prediction, it is important to note that the semiconductor Bloch equations (Methods, "Semiconductor Bloch equations") treat photonic transitions and LZM tunnelling on equal footing. There is no parameter that would favour strong-field tunnelling over the perturbative regime, except for the electric field strength *E(t)* itself.

For an intuitive understanding why electron tunnelling dominates over photoexcitation under our conditions, it is instructive to consider the mean kinetic energy of a quasi-relativistic electron oscillating with a Fermi velocity of v_F under the influence of a periodic drive, $\langle \varepsilon_\mathrm{kin}(t)\rangle = \langle p(t) v_\mathrm{F}\rangle$. With $\dot{p}\,(t) = -e\,E_0\cos(\omega t)$ we arrive at $\langle \varepsilon_\mathrm{kin}(t)\rangle = |e|E_0 v_\mathrm{F}/\omega\,\langle \sin(\omega t)\rangle = (2/\pi)|e|E_0 v_\mathrm{F}/\omega$. For the parameters of our experiment (E_0 = 1.57 MV/cm, $\omega = 2\pi \times 30$ THz, $v_\mathrm{F} = 10^8$ cm/s) we obtain a pondermotive potential of $U_p = \langle \varepsilon_\mathrm{kin}(t)\rangle$ = 530 meV. Since this value exceeds the energy gain of an electron by photon absorption ($\hbar\omega$ = 124 meV) one expects electric field driven processes to dominate. This is essentially the reasoning behind the introduction of the Keldysh parameter γ to distinguish between weak-field and strong-field regime of light-matter interaction. In case of graphene, the Keldysh parameter is commonly defined as $\gamma = \frac{\omega\,\Delta}{2 v_\mathrm{F}|e|E_0}$ with Δ denoting the transition energy [Higuchi *et al.*, Nature **550**, 224 (2017)]. The parameter scales proportionally with the light frequency ω and inversely proportional with the electric field amplitude E_0. If we identify Δ with the MIR photon energy of 124 meV, γ becomes 0.075.

γ can also be interpreted as the ratio of the tunnelling time of an electron to the oscillation period of the electric field [Keldysh, Soviet Physics JETP **20**, 1307 (1965)]. Only for $\gamma \gg 1$ can perturbative transitions, which occur over several cycles, play the dominant role. This clearly indicates that our measurement explores light-matter interaction far in the strong field regime. Moreover, due to the strong deflection of the Fermi circle, the energy gap Δ that is relevant for field-driven interband transitions can even approach zero.

We have modified Extended Data Fig. 6 to show the evolution of the carrier distribution at 30 THz and lower fields (change #23), and added a discussion to the main text to clarify this point (change #7). In the revised manuscript, we clearly state that the observed extreme carrier redistribution on subcycle timescales is a hallmark of non-perturbative (strong-field) dynamics (change #3). Moreover, we have added a new Methods section, which follows the above discussion to derive the Keldysh parameter in graphene (change #13). The role of the Keldysh parameter in the experiment is now also highlighted in the text (change #25). Finally, we added a discussion connecting the shape of the observed carrier distribution to the original formular for diabatic LZM transitions (change #8).

Referee #2: *Furthermore, the authors invoke the 2w oscillation of the Gaussian width as support for LZM dynamics. However, this feature could equally arise from photoexcitation-induced motion of the electron occupation disc in momentum space. When the disc is sufficiently displaced, Pauli blocking is lifted, allowing excitation to occur. This process would repeat twice per optical cycle, showing the 2w modulation. Thus, unless the authors can rigorously rule out this alternative explanation, the observed oscillation cannot be taken as definitive evidence for LZM transitions in momentum space, which is the primary claim of the manuscript.*

Reply 2.3: We can indeed rigorously rule out the scenario described by the Reviewer for the following reasons: (i) As described above, perturbative single- and multi-photon absorption proceeds on a multi-cycle time scale, not on the subcycle scale observed in our experiment. (ii) Perturbative photoexcitation does not cause a displacement of the electron distribution and can thus not lift Pauli blocking. (iii) Perturbative photoexcitation follows the pseudo-spin selection rules described in Fig. R4 which lead to a different momentum space distribution than observed in our experiment. On a more fundamental level, the suggested scenario would imply that the strong-field-regime of field-driven subcycle intraband currents can coexist with perturbative (weak-field) interband photon absorption. Yet, such a cross-over regime cannot exist.

The main evidence for LZM transitions lies in the combination of a subcycle increase of the population in the conduction band and the elongated shape of the electron distribution, which is the result of intraband motion and simultaneous strong-field-driven tunnelling. The 2ω oscillations in the width of the electron distribution along k_y even represent a hint towards Landau-Zener-*Stückelberg*-Majorana (LZ*S*M) interferometry, i.e. interference of coherently driven electrons due to their accumulated phase between two (or more) individual tunnelling events. This is highlighted in Extended Data Fig. 9, which investigates the structure of the carrier distribution for different dephasing times. It shows how the evolution of the width parallel to the field changes from a simple step-like increase for very short dephasing to developing a more pronounced 2ω oscillation caused by the interference.

We have added corresponding discussions to the manuscript to show the absence of perturbative one- or multi-photon absorption in the observed low-frequency strong-field regime (change #5) and the possibility of LZ*S*M features (change #9).

Referee #2: *In summary, while the authors are likely correct in attributing the dynamics to LZM processes, at least in part, and the data are fully consistent with this interpretation, the current manuscript does not provide conclusive proof that LZM transitions are unambiguously observed. The claim of a "first direct observation" in momentum space requires stronger, more rigorous evidence.*

Reply 2.4: We hope that we have eliminated the gaps in our reasoning in Replies 2.2 and 2.3.

Referee #2: *Additional concerns requiring clarification and improvement:*

1. Dark corridor and non-equilibrium effects: The argument that the dark corridor is irrelevant around the Dirac point is questionable here. With electrons excited up to ~1 eV, the dark corridor may significantly influence the measured and analyzed asymmetry of the distribution in the conduction band. This must be explicitly addressed in the theoretical modeling and analysis.

Reply 2.5: With our statement "The influence of the dark corridor ... is suppressed for energies close to the Dirac point..." we did not what to imply that it is *irrelevant*. On the contrary, we carefully chose the geometry in a way that the dark corridor does not affect the electrons driven along the direction of the electric field where their energy gain is maximal (~1 eV). In the perpendicular direction, the maximal observed energy is less (~ 0.5 eV). Photoemission matrix element effects leading to the dark corridor far away from the Dirac point would lead to a small suppression of intensity in negative k_x direction versus positive k_x direction. Such a small asymmetry (~10%) could in principle be present in the 2D momentum maps like the ones shown in Fig. 3. Compared to strikingly different width variations ($\Delta\sigma$) in k_y versus the k_x direction, this effect is small, and we neglect it here.

In principle, since the azimuthal dependence of the matrix element is well understood [Kern *et al.*, Phys. Rev. Research **5**, 033075 (2023)], one could of course take it into account for a full quantitative experiment-theory comparison. This could be done either on the theory side or by renormalizing the experimental data (see e. g. [Reimann *et al.*, Scientific Reports **13**, 5796 (2023)]). Given the qualitative discussion of the theory results we refrained from doing this here.

We have expanded the discussions regarding the matrix element in the manuscript (change #11).

Referee #2: *2. Figure 1b: Should the light blue area in the VB be the signature of holes and is this correct as plotted? Wouldn't it be better to plot this for the doped graphene system, as is used in the experiment?*

Reply 2.6: Yes, the light blue area represents holes in the valence band. The schematic is a simplification of corresponding theory calculations. We chose to schematically show the undoped case, to give the easiest introduction to the concept of LZM transitions for the reader. We have included simulations with different doping levels in Extended Data Fig. 8 for reference (change #26). They show that, within our experimental conditions, the signatures for the undoped and slightly doped case are very similar.

Referee #2: *3. Unexplained features in Fig. 2b (middle): The presence of unexplained "holes" in the valence band, particularly visible in the positive momentum direction, raises concerns about the reliability of the Gaussian fitting*

procedure used in the conduction band. Such artifacts could falsify the extracted momentum distributions and must be investigated and explained.

Reply 2.7: It is certainly correct that the curvature filter based on a second derivative enhances small features and can result in artifacts if applied to noisy data. In the case Fig. 2, curvature filtering has solely been applied to enhance the visibility of the subcycle changes of the raw data plotted in Extended Data Figure 2b. The current has been extracted directly from the raw photoemission data, not from the curvature filtered data. The same applies to all the other data analysis of the manuscript, such as the widths ($\Delta\sigma$) and the centre of mass (COM) displacements shown in Fig. 3. Here, a Gaussian filter with a small window size of 0.02 $Å^{-1} \times 0.02$ $Å^{-1}$ has been applied to the 2D data. Such a filter is necessary to reduce numerical or binning artifacts on the theory side (Fig 4). We simply applied the same filter to the experimental 2D data for the better comparison between experiment and theory. Due its small window size, the filter does not change or remove any of the features retrieved for data analysis.

Referee #2: *4. Figure 2: The phrase "a similar measurement" is vague and should be clarified.*

Reply 2.8: This refers to an additional measurement acquired under almost identical experimental conditions where special care has been taken to align the MIR polarization such that the electric field exhibits no perpendicular component leading to coherent oscillations of the integrated photoemission intensity. Except for these unrelated oscillations the population extracted from the data set of Fig. 2 shows the same overall rise and decay.

We have clarified this point in the revised manuscript (change #21).

Referee #2: *5. Band alignment in Fig. 2: The experimental band dispersions do not align well with theoretical predictions, especially between panels 2b (middle) and 2b (right). As an idea: this discrepancy might be corrected if substrate-induced band modifications are considered. The authors might try to incorporate known effects from graphene on SiC (e.g., as reported by Zhou et al., Nature Materials 6, 770 (2007)) into their analysis.*

Reply 2.9: The slightly transparent grey line in Fig. 2 is a guide to the eye representing the equilibrium Dirac dispersion with a Fermi velocity of 10.7 Å/fs. As the Reviewer recognizes, there might be some small discrepancy between this and the observed bands for delay values where the field is applied, while there are none at equilibrium. Regarding the suggested substrate-induced band modifications, in the suggested paper a gap of ~275 meV is observed for monolayer graphene of 6H-SiC. Our graphene layer is rotated by 30° with respect to this conventional epitaxial graphene [Bocquet *et al.*, Physical Review Letters **125**, 106102 (2020)]. The rotation results in a reduced coupling to the substrate and a band gap of less than 140 meV [Yin *et al.*, Physical Review Materials **9**, 044003 (2025)]. This is not only beyond our energy resolution, it also makes very little difference for the dynamics because, under our experimental conditions, the probability for Landau-Zener Majorana transitions is appreciable for a gap up to 400 meV (change #19). More likely the small deviations (see also Extended Data Fig. 9) are caused by minor residuals from streaking even after compensation. They might also be connected to the energetic broadening in the occupied states that appears once the field is applied and which is not fully understood yet. Both effects could be caused by a small out-of-plane field component which can sometimes be present, as it is very hard to perfectly align the polarization of the field to the sample surface. However, since energy streaking is symmetrical along k this does not affect the analysis of the currents or the shape of the electron distribution.

Referee #2: *6. Figure 3a: It is unclear whether the curvature filtering was applied to the raw data or to a pre-processed dataset. The analysis procedures, throughout the manuscript, must be explicitly described, including details on data integration in energy and momentum space, background subtraction, and the treatment of curvature.*

Reply 2.10: We expanded the discussion of curvature filtering in the Methods to include details about initial binning of the measured data and the data processing (change #20). Our general strategy is to keep the data as raw as possible before any analysis is performed and to use filtering only to improve visibility in the figures.

Referee #2: *7. Visualization and interpretation: Color bars could be added to all relevant figures to clearly indicate intensity scales and the spectra where curvature filtering was applied. This would greatly improve interpretability and transparency.*

Reply 2.11: We have added colour bars to the figures (change #27).

Referee #2: *8. Analysis methodology: The authors must provide a detailed description of how momentum widths and other fitting parameters were extracted: e.g., whether from raw data, integrated regions, or filtered data. The integration areas, fitting windows, and error estimation procedures should be clearly documented.*

Reply 2.12: We clarified and summed up the details on the extraction of different parameters in the previously mentioned section about data processing (change #20).

Referee #2: *9. Data availability: The raw experimental data underlying the presented results have undergone extensive processing, including corrections of momentum and energy shifts, background subtraction, curvature filtering, integration over different momentum and energy windows, and Gaussian fitting. As currently presented, the analysis pipeline is not transparent, and the lack of access to the original, unprocessed data and the used analysis scripts severely limits the ability of readers to verify, interpret, or reproduce the key findings. Given the extraordinary claims made in the manuscript, particularly the first direct observation of LZM transitions in momentum space, full reproducibility is essential.*

In my opinion, the authors should make the raw data, along with detailed documentation of all analysis steps, including integration regions, filtering procedures, correction algorithms, fitting algorithms, and parameter choices, publicly available. This would greatly enhance the scientific rigor and transparency of the work, aligning with best practices for data sharing in modern physics research.

Reply 2.13: While we disagree with the notion that data processing is unusually heavy (as noted above, our aim was to keep the analysis as close to the raw data as possible), we fully agree that transparency is essential in modern scientific work to ensure reproducibility. We acknowledge that a clearer summary of the processing steps (or lack thereof) would benefit the reader and have therefore included a more detailed discussion in the Methods section, as described above (change #20). The data will be made available upon publication.

Referee #2: *As a final note, I would like to express growing concern about our increasing tendency in scientific manuscripts to prioritize narrative flair. Phrases such as "notoriously elusive" (is this really the case for LZM transitions?), "interband quantum leaps" (instead of "interband transitions"), "videography" (used in place of "electronic structure dynamics" in the title), or the suggestion that momentum distributions "conspire" to produce a certain pattern risk undermining the rigor and objectivity expected in high-impact research. While vivid language can enhance communication, its overuse can detract from scientific clarity and credibility.*

It is the authors' decision how to phrase the manuscript, but they should be aware that such stylistic choices may increasingly annoy readers, especially when they appear to prioritize rhetorical effect over precision.

Reply 2.14: We share the Reviewer's point that scientific rigor and linguistic precision must be top priorities in scientific communication. Our choice of terminology tries to follow this principle as we would like to explain for the examples picked by the Reviewer:

- "interband quantum leaps": We are trying to distinguish perturbative quantum jumps that are vertical in momentum space from nonperturbative interband transitions that can occur when an electron follows the band structure while changing its momentum. The generic term 'interband transition' is not quite specific enough to make this distinction. The terms 'quantum jump' and 'quantum leap', in contrast, were already used literally by the founding fathers of quantum mechanics and should therefore be sufficiently established as rigorous scientific terminology. To avoid the false impression that we are attempting to create an unscientific style of language, we replaced 'quantum leap' with the historic term 'quantum jump' (change #2).
- "subcycle band-structure videography": We believe that we are reporting the first slow-motion movies of complete band structures over the entire first Brillouin zone with a time resolution faster than a cycle of light. We would therefore like to reserve the right to name the new concept as accurately as possible. The term 'videography' refers to the process of capturing moving images to create videos, and that is what we wish to convey. In the revised version, we now also use the term 'electronic structural dynamics' suggested by the Reviewer (change #2), but this term describes the object of study rather than our new concept for recording videos of the band structure.
- "notoriously elusive": Ever since strong-field theories started to predict that interactions between inter- and intra-band transitions are responsible for the generation of high harmonics in solids, experiments have sought to identify the characteristic features of both types of transition. The discussion was unnecessarily controversial as no experiment had previously observed these effects directly in the band structure. To describe the long history of futile attempts, we chose the pithy phrase 'notoriously difficult'. However, this term is not essential. In the revised version of the manuscript, we have deleted the word 'notoriously'.
- We replaced the word 'conspire' by 'interact'.

Referee #2: *Conclusion:The experimental results are groundbreaking and represent a major step forward in ultrafast momentum-resolved spectroscopy. The potential for observing LZM transitions in momentum space is*

highly significant and worthy of publication in Nature. However, the current manuscript falls short of providing the rigorous, unambiguous evidence required to support such a bold claim.

I strongly encourage the authors to address the above concerns, particularly by simulating and comparing the expected photoexcitation patterns at 30 THz under realistic experimental conditions, and by rigorously ruling out alternative explanations. If these points are satisfactorily resolved, I would be fully supportive of a resubmission to Nature.

Reply 2.15: We thank the Reviewer for the careful review of our work and for highlighting its impact. We also appreciate the constructive suggestions for improving the presentation and sharpening the narrative.

Replies to Referee #3

Referee #3: *The work by V. Eggers et al. demonstrates sub-cycle mapping of k-resolved electron population dynamics under strong fields in graphene using time-resolved ARPES, with unprecedented access to the full Brillouin zone. In particular, the paper focuses on visualizing light-driven carrier acceleration and non-adiabatic Landau-Zener-Majorana transitions with sub-cycle resolution.*

The paper shows direct observation of light-driven acceleration of carriers along the polarization direction of the field (ky), reversing with each half-cycle, from which the intraband current is extracted. This current is shown to be phase shifted relative to the driving field by less than pi/2, which is the value expected from purely coherent acceleration of carriers by the field. In addition, it is shown that the shape of the k-resolved light-driven electron distribution is elliptical, with the widths along the kx and ky directions changing periodically, and gradually evolves to an almost circular shape approaching (but not reaching) the equilibrium value for 233fs.

These experimental observations are reproduced and explained by solving the semiconductor Bloch equations of a two-band tight binding graphene model that includes a phenomenological dephasing T2, and approximate electron-electron scattering and electron-phonon scattering rates. These calculations identify that (i) interband coupling is responsible for the elongation of the electronic distribution along the field polarization direction, (ii) electron-electron and electron-phonon coupling are responsible for the thermalization of the distribution in a circular shape, and the latter is responsible for the deviation of the current phase shift from that of fully coherent dynamics.

The work represents a clear experimental advance by extending sub-cycle ARPES to access the full Brillouin zone and provides a compelling visualization and picture of strong-field dynamics in graphene. The results are clearly presented and both experimental and theoretical methodologies are of high quality and convincing (e.g., appropriate compensation of streaking in the continuum, obtention of e-e and e-ph scattering rates).

Reply 3.1: We thank the Reviewer for taking the time to review our manuscript and their helpful suggestions. We are grateful for their recognition of the quality of this work.

Referee #3: *One issue is that despite the main experimental advance being the full BZ access (+- 2.14 Angstroms^-1), all analyzed dynamics are confined near the K point (|k - K| < 0.16 Angstroms^-1). The physics explored (Dirac cone + LZM tunnelling) is inherently two-band (as the theoretical approach demonstrates) and already accessible with the momentum range of previous experiments (e.g., S. Ito et al. Nature 616 (2023)). It seems that the extended momentum range does not play an essential role in the present study, and its potential to explore multi-band physics away from K (e.g., near Gamma) is unused. Before reaching a decision, I would like the authors to address the following comments.*

Reply 3.2: While the dynamical relative changes at a given K point of graphene are indeed discussed in an area that only moderately exceeds the accessible momentum range of our previous experiment (roughly 50%), this does not apply to the absolute momentum range. One has to keep in mind that every single K point is located ~1.7 $Å^{-1}$ away from the BZ centre. To photoemit an electron from any of these K points (90° grazing emission) a minimum photon energy of 15.3 eV is necessary.

$$k_{||}\left(Å^{-1}\right) = 0.514\sqrt{E_{\mathrm{kin}}(\mathrm{eV})}\ \sin\theta$$

Obviously, the HHG XUV beamline is thus essential for the present experiment. The other major advance compared to Ito *et al*. lies in the use of a momentum microscope instead of a hemispherical analyser for electron detection. This instrument not only makes electrons detectable that are emitted at grazing angles. It also enables full 2D momentum mapping of the photoelectrons, which is crucial for analysing the shape of the electron distribution in momentum space. Both capabilities have been decisive for the success of the present experiment. The Reviewer is, of course, correct in the observation that our new setup can track electrons moving over the entire BZ and the LZM dynamics have not necessitated us to utilize this possibility for graphene. Yet, we are working on experiments with different materials where we will exploit exactly this capability.

In the revised manuscript we clarify this point (change #1).

Referee #3: *1. It is unclear why a single Fourier-averaged phase shift of the current is extracted. Since this phase shift is attributed to scattering, it should depend on time, carrier density and k-space distribution. Therefore, this should not lead to a constant phase shift, but rather to a chirp. Indeed, in Figure 2c one can observe that the phase shift is reduced in the trailing edge compared to the peak of the field. Do the simulations reproduce this time-*

[redacted]

dependent phase shift? Perhaps a local time-frequency analysis would provide a more meaningful characterization of the dynamics.

[redacted]

Referee #3: *2. In the theory, relaxation processes are effectively described as driving the system towards the field-free equilibrium distribution. While this is a common approximation, it is a still open question whether under such strong-field conditions and fast timescales the system indeed relaxes towards the initial equilibrium state, rather than towards a transient hot-carrier distribution. Do the authors have access to the experimental population distributions at longer times (> 250 fs)? If so, are they different than the initial, field-free equilibrium distributions? And, if so, do the dynamics predicted by theory change in some way by using these final "transient" electron distributions as the target for relaxation rather than the initial field-free equilibrium populations? More generally, given the limited energy resolution, it is not entirely clear whether the observed isotropic contraction should be interpreted as true thermalization to a Fermi-Dirac distribution, rather than as a relaxation towards a more isotropic but still non-equilibrium transient state.*

Reply 3.4: We thank the Reviewer for their comment, which gives us the opportunity to clarify our theoretical analysis of scattering. The relaxation time approximation (Methods, "Relaxation time approximation") was used solely to relate the experimental phase delay to a phenomenological scatting time (Extended Data Fig. 3). For all other parts of the paper, our treatment of scattering processes goes beyond the relaxation time approximation and does not include any "target" for the relaxation process (Methods, "Modelling of the scattering rates"). The scattering rate for electron-phonon interaction (line 422, page 14) does not depend on the electron distribution at equilibrium. In the simplified expression for the electron-electron scattering rate (line 470, page 15) we used the equilibrium distribution only to evaluate the effective transition rate (lines 472-473, page 16). In general, therefore, our theoretical approach does not enforce any specific electron distribution at any time instant and still remarkably matches our experimental results (Fig. 4, Extended Data Fig. 10, and related discussion). We revised the Methods section to explicitly underline this point (changes #17, #18).

With respect to the long-term behaviour, we would like to stress that the main target of the present investigation is the subcycle dynamics of a system driven far out of equilibrium. The high time resolution inevitably goes hand in hand with a limited energy resolution, and we cannot detect small deviations from a Fermi-Dirac distribution as the Reviewer correctly observes. When we wrote "... isotropically contracts towards equilibrium", we did not mean to imply that. In the revised version of the manuscript, we write instead "... contracts to a more equilibrated and isotropic distribution." (change #10). In principle, our experiment can measure energy- and momentum resolved populations distributions over time scales of many picoseconds. This is, however, beyond the scope of this work and should better be done with a setup that is not targeted towards subcycle temporal but meV energy resolution.

Referee #3: *3. While k-resolved scattering is implicit in the theory, it is not clear how it plays a role in the dynamics, especially when the work restricts dynamics to a narrow region around the K point and interprets the results with a single effective scattering time. Given the momentum-resolved nature of the experiment, it would be very valuable to clarify to what extent momentum-dependent scattering plays a role in the observables; the technique presented here seems ideally suited to probe such k-dependent scattering mechanisms. Could the authors comment on whether this could be accessed experimentally, for example by varying the driving field amplitude or frequency (to explore different k-space regions), or by performing measurement at lower field strengths to probe near-equilibrium scattering? This would give a more direct characterization of strong-field-driven scattering processes.*

Reply 3.5: The Reviewer is certainly correct: momentum-resolved scattering dynamics are important for reproducing the experimental data. While momentum-integrated quantities, such as the relative timing of the current density (Fig. 2c), could be reproduced with a single effective scattering time in the relaxation time approximation (Extended Data Fig. 3), only a detailed momentum-resolved analysis is able to match the detailed evolution of the electron occupation in momentum space (Fig. 4). The possibility of experimentally accessing this type of information is clear if we compare the time-dependent electron distribution obtained for different scattering mechanisms (Extended Data Fig. 8). The peculiar angular dependence of electron-electron scattering, often referred to as collinear (see, e.g., Brida *et al.*, "Ultrafast collinear scattering and carrier multiplication in graphene." Nat. Commun. **4**, 1987 (2013), Ref. 40 in the manuscript), allows maintaining an anisotropic electron distribution around the K point (Extended Data Fig. 8b, t = 27 fs). The combined effect of scattering with Γ longitudinal and transverse optical phonons leads instead to an angularly isotropic scattering rate, which quickly deforms the electron distribution to a circular shape (Extended Data Fig. 8c, t = 27 fs). Only by combining both scattering mechanisms can we qualitatively reproduce the experimental data.

As correctly pointed out by the Reviewer, then, carefully tailoring the light-matter interaction process it could be possible to engineer the electron distribution in momentum space and, in turn, directly access individual scattering mechanisms. As an example, the injection of valley-polarized carriers as suggested, e.g., in Gill *et al.*, "Valley polarization of graphene via the saddle point." npj Comput. Mater. **12**, 167 (2026), in combination with momentum microscopy could give access to both inter- and intravalley momentum-dependent scattering dynamics, as demonstrated, e.g., by Wallauer *et al.*, "Intervalley scattering in MoS_2 imaged by two-photon photoemission with a high-harmonic probe." Appl. Phys. Lett. **109**, 162102 (2016), as well as Refs. 12 and 15 of the manuscript. However, these dynamics are beyond the scope of the present work and are left for future investigations.

Referee #3: *4. The extracted current is based on population asymmetry and therefore corresponds to the intraband contribution. I assume that the theory neglects the interband current contribution (not the coupling), but it would be useful to clarify if the current in the theory is extracted in the same way as in the experiment.*

Reply 3.6: We are grateful for the Reviewer's comment, which helps us improving the clarity of our manuscript. The current density is extracted, both in the theory and in the experiment, from the asymmetry in the occupation

of the band structure. As correctly pointed out, therefore, we are exclusively accounting for the intraband contribution to the total current density. To make this point clearer in the manuscript, we now introduce this quantity as an "instantaneous asymmetric electron distribution in momentum space […] associated with […] a microscopic *intraband* current" (change #4). Moreover, we added the specifier "intraband" to describe the term "current density" in multiple instances throughout the manuscript (change #14) and explicitly mention that experimental and theoretical data are treated in the same way to extract intraband current densities (change #15).

Referee #3: *5. The polarization of the driving field is chosen along ky, where the two branches of the Dirac cone have the same photoemission intensity. I assume this choice is made in order to minimize the effect of different matrix elements in the measured ARPES signal. Is this correct? Assuming the photoemission matrix elements are well characterized, are there any limitations apart from this one to explore the dependence of the dynamics on the driving field polarization?*

Reply 3.7: Indeed, the relative orientation of the field polarization is chosen such that photoemission matrix elements affect the two relevant directions ($+k_y$ and $-k_y$) in the same way. Since photoemission matrix elements of graphene are well understood it is principally possible to systematically explore the dependence on the driving field polarization. In the present setup, however, this can only be done by rotating the sample. Therefore, the direction of the incident electric field and the position of the so-called "dark corridor" of graphene cannot be changed independently of each other. This makes the data evaluation difficult and poses experimental challenges in terms of signal-to-noise ratio. Of course, this is not a fundamental restriction of subcycle momentum microscopy, and experiments as the ones the Reviewer has in mind will very well become possible in the future.

List of changes

#	Lines	Change
1	49	Clarified that the experiment accesses electron dynamics at the Brillouin-zone edge, highlighting the importance of the large momentum-space coverage enabled by the setup.
2	56, 72	Added the historic term “quantum jump” to replace “quantum leap”, and the term “electronic structural dynamics” to describe the object of our subcycle band-structure videography study.
3	92-96	Added an explicit statement explaining that the observed subcycle carrier redistribution is a hallmark of non-perturbative strong-field dynamics and explicitly stating the Keldysh parameter for the experiment.
4	97-99	Clarified that the asymmetric electron distribution is associated with a microscopic *intraband* current.
5	114-118	Highlighted the shortcomings of describing dynamics through perturbative absorption.
6	125-126	A “streaking-compensated” to the description of the measured momentum maps.
7	150-157	Expanded the discussion contrasting perturbative photoexcitation with LZM-driven dynamics and linked the interpretation to the new Extended Data Figs. 6 and 7.
8	160-167	Added a discussion that connects the k_x extend of the observed carrier distribution to the original formula for diabatic transitions.
9	173-178	Clarified the interpretation of the observed 2ω width oscillations, emphasizing the possible role of Landau-Zener-Stückelberg-Majorana interference.
10	185	Replaced “equilibrium” with “a more equilibrated and isotropic distribution”.
11	267-272	Expanded the discussion of photoemission matrix-element effects and the dark corridor.
12	294-303	Revised the explanation of the streaking-compensation procedure.
13	322-343	Added a new Methods subsection, “Keldysh parameter in graphene”, deriving the graphene-specific form of the parameter and showing that the experiment operates deeply in the strong-field regime.
14	345	In consistency with change #4, we refer to the microscopic *intraband* current density.
15	359-360	Explicitly stated that experimental and theoretical data are analysed with the same current-extraction procedure.
16	376-381	Added a discussion of time-resolved phase-delay analysis and its potential for resolving scattering dynamics.

17	482-484	Clarified that the scattering model does not enforce relaxation toward a predetermined electron distribution while still reproducing the experimental data.
18	486	Added context explaining that the relaxation-time approximation is used specifically to relate measured phase delays to an effective scattering time.
19	497-516	Added a new Methods subsection, "Landau-Zener-Majorana transition region", introducing the diabaticity parameter and the diabatic transition probability in graphene.
20	548-602	Added a comprehensive Methods section on "Data processing", which documents binning, filtering, integration windows, fitting procedures, and analysis workflows used throughout the manuscript.
21	579-582	Clarified the origin of the conduction-band population data.
22	Extended Data Fig. 2	Revised Extended Data Fig. 2 to include the data from the previous Extended Data Fig. 11, to reduce the overall number of figures.
23	Extended Data Fig. 6	Revised Extended Data Fig. 6 to compare weak- and strong-field behaviour at the experimentally relevant driving frequency.
24	Extended Data Fig. 7	Added new Extended Data Fig. 7 (here shown as Fig. R3), providing field-dependent simulations that distinguish perturbative excitation from strong-field dynamics.
25	873-882	Emphasizing the Keldysh-parameter dependence of carrier excitation and distribution anisotropy.
26	Extended Data Fig. 8	Revised Extended Data Fig. 8 to illustrate the influence of doping and initial occupation on the simulated carrier dynamics.
27	Throughout manuscript	Added colour bars to all figures to improve transparency and quantitative interpretation of the data.
28	Throughout manuscript	Followed suggestion by Reviewer #2 to adjust the language throughout the manuscript.
29	Throughout manuscript	Added terms like "strong-field" and "subcycle" to the describe the dynamics throughout the manuscript to underline the nature of the light-matter interaction.
30	Throughout manuscript	Condensed the main text whilst preserving all relevant details and without altering the claims made in the manuscript.
31	Throughout manuscript	Corrected typos and improved the clarity of the text throughout the manuscript.

Replies to Referee #3

Referee #3.1: *The authors have substantially improved the clarity of the manuscript and addressed my questions. 1. My concern was about the actual use of the novel experimental capabilities in the presented work. The authors have clarified that, while the present study does not make use of the ability to track dynamics across the full Brillouin zone, it does rely on both the access to the K points at the BZ edge and 2D momentum mapping with sub-cycle temporal resolution.*

Previous works have demonstrated XUV tr-ARPES of carrier dynamics at the graphene K point (I. Gierz et al., Phys. Rev. Lett. 115 086803 (2015)) and 2D momentum mapping covering the full BZ (e.g., S. Dong et al., Nature Comm. 14 5057 (2023)). Yet, the present work advances these techniques by reaching sub-cycle resolution with the use of mid-IR pulses. This sub-cycle resolution is indeed central to support the observation of LZ transitions, and is thus a key advance.

Reply 3.1: We added the reference to Dong *et al.* to the list of other references reporting 2D band mapping over the full BZ (change #4). With respect to the work by Gierz *et al.,* we would like to stick to their earlier publication [I. Gierz, et al., Nature Materials 12, 1119 (2013), ref. 42] as it more pioneering and more fundamental in our view than the PRL from 2015. Their 2013 paper is also the one that is most recognized by the community.

Referee #3.4: . *I thank the authors for the clarification. Just a small note that I'm certain the authors are aware of but might help the readers not getting confused: strictly speaking, the measured population asymmetry is not itself and intraband current, since the current also requires to multiply the populations by the band velocity. Perhaps there are some instances in the paper where intraband can be changed or clarified. I am happy with the term "instantaneous asymmetric electron distribution in momentum space […] associated with […] a microscopic intraband current" that the authors use.*

Reply 3.4: The Reviewer is correct. We slightly adapted the Methods to clarify the section about the conversion from intraband occupation imbalance to intraband current (change #3).

Referee #3.other *Regarding the other Referee's remarks, I found that they were properly addressed, although I found the answer regarding multi-photon/tunnelling dynamics somewhat confusing. The authors use the "Keldysh" parameter used by Higuchi et al., which is reasonable, but this parameter is momentum-dependent; it is not a universal parameter, rather it shows that different parts of the BZ of graphene will have different "Keldysh" parameters. The authors choose precisely the momentum point with gap resonant to the one-photon transition to obtain a parameter much less than one, and use it to justify the tunnelling picture. First, it is somewhat misleading to choose a one-photon resonant region to justify that excitation occurs through tunnelling and second, even if that is the case, that only implies that the "Keldysh" parameter is less than one for that particular momentum. For larger momenta, the "Keldysh" parameter might be comparable to 1. A stronger argument would be therefore to show that all momenta relevant for the LZ dynamics show a "Keldysh" parameter much smaller than one. That said, I don't think this is a fundamental issue, since I believe the physics observed by the authors do show evidence of LZ transitions: the CB population increase and the elongated shape of the population distribution.*

I do not believe the authors should try to justify that dynamics are governed solely by tunnelling. In that sense, the statement of Extended Data Figure 7: "[...] marks the absence of perturbative single- or multi-photon absorption" is too strong, but also not needed. Residual photonic contributions would not invalidate the central point.

Reply 3.other: We followed the suggestion of the referee and added a note about the Keldysh parameter in the momentum range where tunnelling is observed (change #2) and slightly toned down the statement about missing perturbative single- or multiphoton absorption (change #6).

List of changes

#	Lines	Change
1	1	Changed the title to "Subcycle videography of Landau-Zener-Majorana transitions in graphene" to fit within the character limit.
2	160-161	Added a remark about the Keldysh parameter in the momentum range where tunnelling is observed.
3	517	Clarified the conversion from intraband occupation imbalance to intraband current.
4	244-245	Expanded the list of reference 12 – 18 with Ref. 19: Dong et al., Observation of ultrafast interfacial Meitner-Auger energy transfer in a Van der Waals heterostructure, Nature Comm. 14 5057 (2023).
5	797-798	Updated the reference of the data repository.
6	891-892	Adapted the statement about missing perturbative single- or multiphoton absorption.
7	Throughout manuscript	Reformatted the manuscript to follow the upload requirements.
